# Comprehensive Splice Pattern Analysis for Previously Reported *OCRL* Splicing Variants and Their Phenotypic Contributions

**DOI:** 10.1016/j.ekir.2025.02.023

**Published:** 2025-03-03

**Authors:** Rini Rossanti, Eri Okada, Nana Sakakibara, Ryota Suzuki, Yuta Inoki, Yuta Ichikawa, Yu Tanaka, Hideaki Kitakado, Chika Ueda, Atsushi Kondo, Yuya Aoto, China Nagano, Tomoko Horinouchi, Tomohiko Yamamura, Shingo Ishimori, Kandai Nozu

**Affiliations:** 1 Department of Pediatrics, Kobe University Graduate School of Medicine, Kobe, Japan; 2 Department of Child Health, Nephrology Division, Dr. Hasan Sadikin General Hospital/Faculty of Medicine, Universitas Padjadjaran, Bandung, Indonesia; 3 Department of Pediatrics, Hokkaido University Graduate School of Medicine, Sapporo, Japan

**Keywords:** Dent disease-2, isoform, Lowe syndrome, minigene system, *OCRL*, Splicing

## Abstract

**Introduction:**

Two distinct phenotypes of Dent disease-2 and Lowe syndrome are caused by *oculocerebrorenal syndrome of Lowe* (*OCRL*) abnormality. Previous genetic studies demonstrated that truncating variants in exons 1 to 7 results in Dent disease-2 and in exons 8 to 24, result in Lowe syndrome. Recently, we successfully identified a functional *OCRL* isoform, whose altered initiation codons (Met187 and Met206) in exon 8 can affect the *OCRL*-truncating variant phenotypes. However, the association between *OCRL* splicing variants and phenotypes is poorly understood.

**Methods:**

We performed a detailed splicing pattern analysis of previously reported 28 *OCRL* splicing variants obtained from the Human Gene Mutation Database. We assessed the variant consequences at the mRNA level using an *in vitro* splicing assay with a minigene system, and examined their compatibility with *in silico* algorithms and correlation with disease phenotypes.

**Results:**

Aberrant splicing was confirmed in all 27 variants, except for 1, in which splicing could not be experimentally confirmed in the minigene system, and therefore could not be concluded with certainty. Splicing variants in *OCRL* exons 1 to 7 resulted in Dent disease-2, and in exons 9 to 24 resulted in Lowe syndrome. In 1 case, c.561-2 A > G in exon 8 demonstrated Dent disease-2.

**Conclusion:**

This study provides significant data on the pathogenicity of *OCRL* splicing variants and genotype-phenotype correlations. In c.561-2 A > G, the latter altered initiation codon of the OCRL isoform (Met206) was preserved, potentially indicating the Dent disease-2 phenotype. This result supports our recent finding regarding the altered initiation codons in exon 8 of the OCRL isoform.

## Graphical abstract

**Figure:**
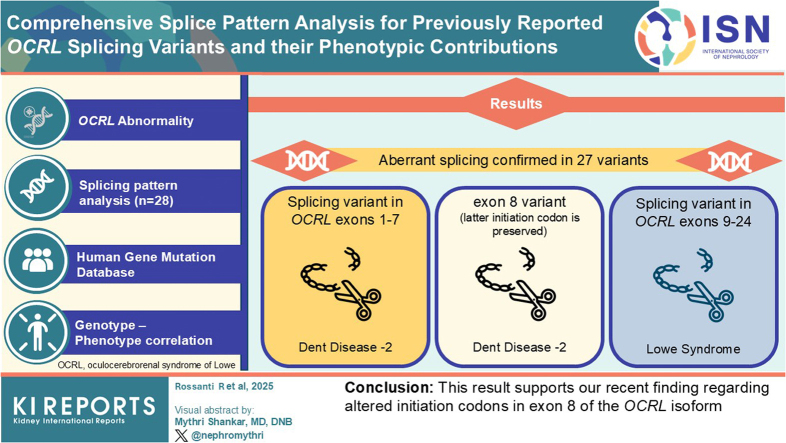

Dent disease-2 and Lowe syndrome are associated with *OCRL* gene variants.^1^ Dent disease-2 (OMIM #300555) is a rare disease characterized by proximal tubular dysfunction with no or mild extrarenal manifestations.^1^^,^^2^ In contrast, Lowe syndrome (OMIM #3009000) is a rare disease exhibiting multisystem involvement, including ocular and central nervous system abnormalities, accompanied by proximal tubular dysfunction in the form of Fanconi syndrome, and it is clinically severer than Dent disease-2.^3^ Although the causative gene for the 2 diseases is the same, the severity of disease differs significantly.

*OCRL* encodes phosphatidylinositol 4,5-diphosphate (PI[4, 5]P_2_) 5-phosphatase, an enzyme that dephosphorylates phosphoinositides^4^^,^^5^ and regulates various cell functions, including membrane transport, cell apoptosis, and cytoskeletal control by regulating phosphoinositide levels.^6^, ^7^, ^8^ *OCRL* contains 24 exons. Previous studies have demonstrated that truncating variants (frameshift and nonsense) associated with Dent disease-2 are concentrated in exons 1 to 7, whereas those associated with Lowe syndrome are concentrated in exons 8 to 24. Therefore, it is hypothesized that an isoform configured with exons 8 to 24 exists and may rescue the systemic malfunction of OCRL in Dent disease-2.^9^^,^^10^ Recently, we successfully cloned novel *OCRL* isoform transcripts configured with exons 6 to 24. In this transcript, functional OCRL isoforms are synthesized from 2 altered translation-initiation codons (Met187 and Met206) in exon 8. Met187 and Met206 are the most likely translation initiation sites followed by a novel 5' UTR region, exon 6 and 7, as indicated by *in silico* analyses.^11^ These findings explain the severity of *OCRL* truncating variants but do not fully explain the severity of splicing variants. This is because splicing variants can cause either frameshift deletion-insertion or in-frame deletion-insertion; moreover, they can cause both normal and aberrant splicing simultaneously. It is necessary to assess how the *OCRL* splicing variant around exon 8 affects the initiation codon of this isoform and determines its phenotype.

The underlying cause of numerous hereditary diseases is presently recognized in altered pre-mRNA splicing.^12^^,^^13^ Various human genetic diseases are caused by aberrant pre-mRNA splicing that accounts for approximately 15% to 60% of all genetic diseases.^14^ Multiple computational (*in silico*) predictive programs have been developed and are commonly used to assess splicing variants.^15^^,^^16^ Although these *in silico* analyses are useful for predicting splicing variants, this is only a prediction, meaning that it is not guaranteed that aberrant splicing actually occurs. Consequently, even if aberrant splicing is predicted by the *in silico* approach, the findings must at least be confirmed by “wet lab” experiments such as the minigene system.^15^^,^^17^ One previous study assessed splicing aberrations and phenotypes in exonic *OCRL* variants. In the study, 13 missense variants and 1 synonymous variant were analyzed and 3 presumed missense variants caused alterations in pre-mRNA splicing. The results highlight the importance of assessing the effects of OCRL exonic mutations at the mRNA level.^18^ However, a comprehensive examination of *OCRL* splicing aberrations has not yet been conducted.

In this study, we performed a comprehensive splicing pattern analysis of *OCRL* splicing variants using a minigene system. We aimed to confirm the contribution of aberrant splicing to disease onset, compare the observed splicing patterns with *in silico* algorithm predictions, and assess the relationship between the *OCRL* splicing variants and their phenotypes.

## Methods

### *OCRL* Variants and Their Phenotype Data

This study included *OCRL* variants reported as splicing substitutions in the Human Gene Mutation Database (HGMD Professional) in September 2021 (Table 1).^2^^,^^9^^,^^10^^,^^19^, ^20^, ^21^, ^22^, ^23^, ^24^, ^25^, ^26^, ^27^, ^28^, ^29^, ^30^, ^31^, ^32^ In HGMD Professional, splicing substitutions are defined as variants with consequences for mRNA splicing. Phenotypes of each variant were determined as described in HGMD Professional. The *OCRL* cDNA reference number is NM_000276.4 (NCBI ID).

**Table 1:** Details of variants analyzed and splice effects observed in minigene system

No.	Variant	Exon number	Disease phenotype	Splice effects observed in minigene system	Protein effect	**References**
1.	c.40-14 A>G	2	Dent-2	No Data	Not evaluable	Ref. 9
2.	c.199+1 G>A	3	Dent-2	exon 3 skipping	p.Leu41Serfs∗8	Ref. 19
3.	c.439+3 A>G	6	Dent-2	exon 6 skipping	p.Ala117_Ser146del	Ref. 20
4.	c.560+1 G>A	7	Dent-2	cryptic exon inclusion loss of exon 7 fragment exon 7 skipping	p.Leu188∗ p.Val184Alafs∗4 p.Leu148Phefs∗15	Ref. 10
5.	c.561-1 G>A	8	Not available	loss of exon 8 fragment	p.Met187Ilefs∗16	Ref. 21
6.	c.561-2 A>G	8	Dent-2	loss of exon 8 fragment exon 8 skipping	p.Met187Ilefs∗4 p.Met187_Arg241delinsIle	Ref. 2
7.	c.723-1 G>A	9	Lowe	exon 9 skipping	p.Phe243_Phe276del	Ref. 22
8.	c.824 G>C	9	Lowe	exon 9 skipping	p.Phe243_Phe276del	Ref. 23
9.	c.825-2 A>G	10	Lowe	inclusion of ivs9 to exon 10 loss of exon 10 fragment	p.Gly275_Phe276insArg∗ p.Phe276Thrfs∗12	Ref. 9
10.	c.939+3 A>C	10	Lowe	loss of exon 10 fragment	p.Leu305Serfs∗24	Ref. 24
11.	c.940-11 G>A	11	Lowe	inclusion of ivs10 to exon 11	p.Lys313_Val314insAsnSer∗	Ref. 25,26
12.	c.940-1 G>A	11	Lowe	loss of exon 11 fragment	p.Val314Phefs∗9	Ref. 27
13.	c.1244+1 G>C	12	Lowe	exon 12 skipping	p.Asn354Cysfs∗10	Ref. 20
14.	c.1466 G>A	14	Lowe	exon 14 skipping	p.Leu453Trpfs∗10	Ref. 23
15.	c.1467-2 A>G	15	Nephrotic syndrome / Lowe	loss of exon 15 fragment exon 15 skipping	p.Gly490Leufs∗3 p.Ser489Argfs∗2	Ref. 28
16.	c.1467-3 C>G	15	Lowe	loss of exon 15 fragment	p.Ser489Argfs∗32	Ref. 9
17.	c.1602+1 G>A	15	Lowe	loss of exon 15 fragment exon 15 skipping	p.Ser528_Val535del p.Ser489Argfs∗2	Ref. 23
18.	c.1603-2 A>C	16	Lowe	loss of exon 16 fragment exon 16 skipping	p.Lys536_537del p.Val535_Glu571del	Ref. 27
19.	c.1714-2 A>G	17	Lowe	exon 17 skipping	p.Phe572Metfs∗17	Ref. 21
20.	c.1879+5 G>A	17	Lowe	exon 17 skipping	p.Phe572Metfs∗17	Ref. 9
21.	c.2256+1 G>A	20	Lowe	exon 20 skipping	p.Lys715_Glu753del	Ref. 29
22	c.2341+1 G>C	21	Lowe	inclusion of ivs 21 to exon 21 and loss of exon 22 fragment (not evaluable)	Not evaluable	Ref. 30
23	c.2469+2 T>G	22	Lowe	exon 22 skipping (not evaluable)	Not evaluable	Ref. 9
24	c.2581 G>A	23	Lowe	exon 23 skipping	p.Val824Leufs∗9	Ref. 31
25	c.2581 G>C	23	Lowe	exon 23 skipping	p.Val824Leufs∗9	Ref. 22
26	c.2581+1 G>A	23	Lowe	exon 23 skipping	p.Val824Leufs∗9	Ref. 30
27	c.2581+1 G>C	23	Lowe	exon 23 skipping	p.Val824Leufs∗9	Ref. 9
28	c.2581+4 A>G	23	Lowe	exon 23 skipping	p.Val824Leufs∗9	Not given

Dent-2, Dent disease-2; Lowe, Lowe syndrome; Ref., Reference.

### *In vitro* Splicing Assay Using Minigene System

#### OCRL Fragment Plasmid Construction

**Figure 1:**
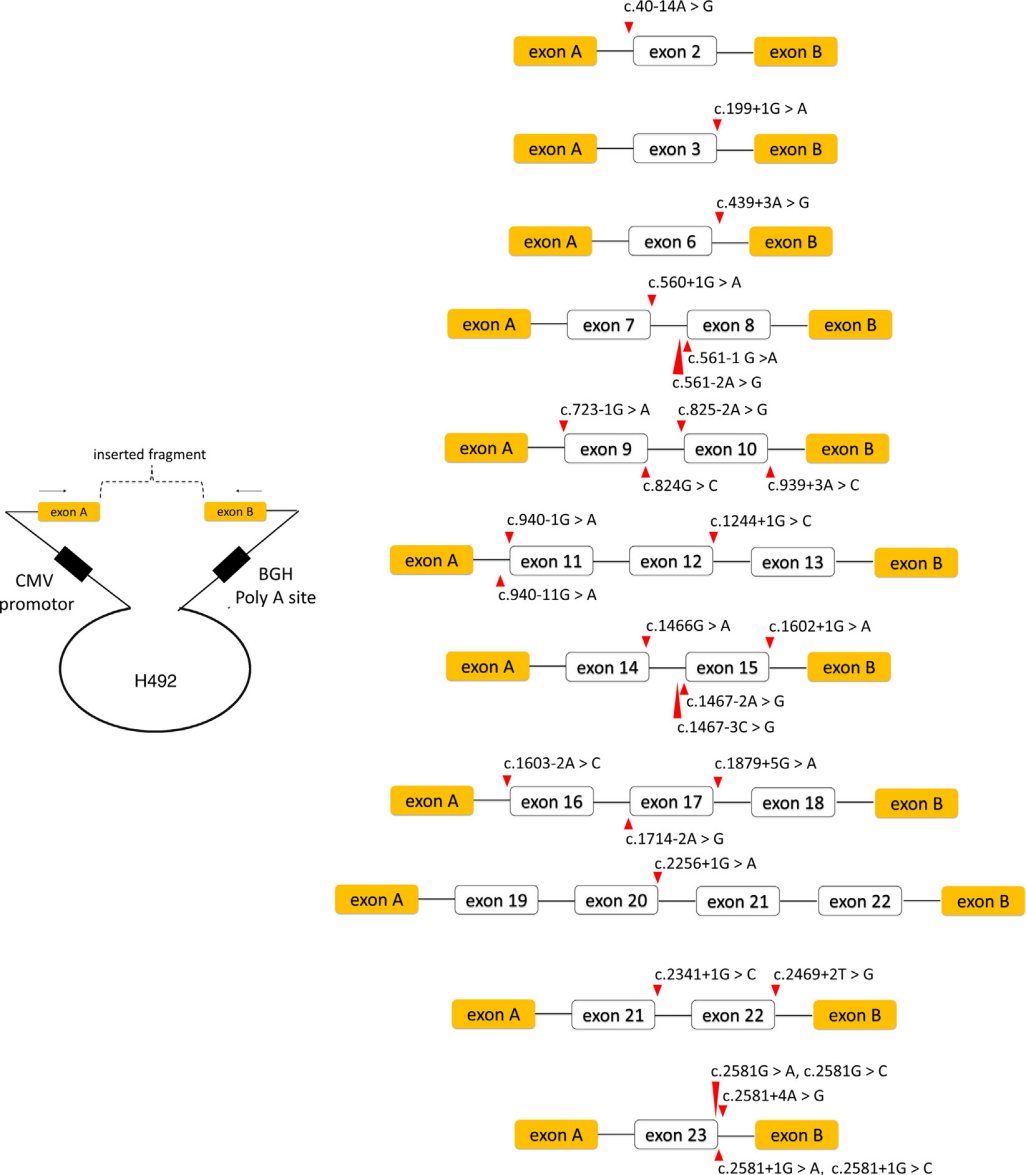
Schematic representation of the minigene constructs. Intron and exon boundaries of the variants are cloned into the H492 vector between exons A and B (yellow box). The white box with the exon number demonstrates *OCRL* wild-type sequences, with the up and downstream introns of the exon (horizontal line). The different variants provided by site-directed mutagenesis in each minigene construction are demonstrated in red arrows. OCRL, oculocerebrorenal syndrome of Lowe.

We used the H492 vector to create hybrid minigene constructs.^33^^,^^34^ We cloned the *OCRL* genomic fragments flanked by upstream and downstream exons obtained from healthy controls between exons A and B of the H492 vector (Figure 1). We used the in-fusion cloning method, following the manufacturer's protocol (Takara Bio, Otsu, Japan). We transformed the *OCRL*-constructed plasmids into *Escherichia coli* HST08 premium competent cells (Takara Bio). Following an overnight incubation, the colony was screened, and the plasmid was purified using the QIAprep Spin Miniprep Kit (QIAGEN, GmbH, Hilden, Germany).

#### Site-Directed Mutagenesis

The *OCRL-*constructed plasmids were converted to variant plasmids using the PrimeSTAR Mutagenesis basal kit (Takara Bio), following the manufacturer’s protocol. Reaction products were subsequently transformed into the *Ecoli* HST08 premium competent cells (Takara Bio). To confirm the presence of desired variants, all constructs were analyzed through directed sequencing using the *OCRL* gene-specific primers. Schematic representation of the minigene constructs is shown in Figure 1.

#### Assessment of Splicing Pattern

*OCRL* variant plasmids were transfected into HEK293T and Hela cells using Lipofectamine 3000 (Thermo Fisher Scientific, Waltham, MA). After 24 hours, total RNA was extracted from the cells using an RNeasy Plus Mini Kit (QIAGEN). Total RNA (1 μg) was subjected to reverse transcription using the RNA to cDNA EcoDry Premix (Double Primed) (Takara Bio). Polymerase chain reaction was performed using a forward primer corresponding to a segment upstream of exon A (YH307: 5`-ATTACTCGCTCAGAAGCTGTGTTGC-3`) and a reverse primer complementary to a segment downstream of exon B (Y308: 5`-CTGCCAGTTGCTAAGTGAGAGACTT). The polymerase chain reaction products were analyzed by electrophoresis on a 1.5 % agarose gel, followed by Sanger sequencing. Capillary electrophoresis was conducted on an Agilent 2100 Bioanalyzer using an Agilent DNA1000 Kit (Agilent Technologies, Santa Clara, CA) when the difference in size of polymerase chain reaction products between wildtype (WT), and variant could not be recognized by normal electrophoresis images.

### *In silico* Splicing Prediction

We assessed the potential effect of splicing variants using multiple algorithms implemented in the software to predict the strength of splice sites. The commonly applied tools, such as GeneSplicer (https://www.cbcb.umd.edu/software/GeneSplicer/gene_spl.shtml),^35^ MaxEntScan (http://hollywood.mit.edu/burgelab/maxent/Xmaxentscan_scoreseq.html),^36^ NNSPLICE(Interactive Biosftware, Rouen, France; http://www.interactive-biosoftware.com),^37^ and SpliceSiteFinder-like(http://www.interactive-biosoftware.com)^38^ were accessed from Alamut Visual (SOPHiA GENETICS, Saint Sulpice, Switzerland). In addition, SpliceAI-lookup, a deep learning-based tool to identify splice variants on a web-based interface (https://spliceailookup.broadinstitute.org/), was used to assess the potential of splicing defects using a Δ Score  >  0.2 as the cutoff.^16^ The variants were computed with 500 bp as the threshold distance between the variant and gained or lost splice sites. Moreover, we assessed the consistency of the minigene system and SpliceAI-lookup. If the minigene system produced exactly 1 splicing pattern identical to that predicted by SpliceAI-lookup, it was described as "perfectly" matched. In contrast, if the minigene system produced multiple splicing patterns with one of them being the same as the one predicted by the SpliceAI-lookup, it was described as "partially" matched. If the splicing patterns differed in both cases, it was described as "not" matched.

## Results

### *In Vitro* Splicing Assay Using Minigene System

We assessed the splicing pattern of 28 *OCRL* splicing variants, and almost all exhibited aberrant splicing in the form of exon skipping, loss of exon fragment, insertion of intron fragment, and inclusion of the cryptic exon (Table 1, Supplementary Figure S1). In addition, certain variants exhibited multiple splicing patterns. Our research newly determined pathogenicity in 3 variants (No.12: c.940-1 G > A, No.15: c.1467-2 A > G, and No.18: c.1603-2 A > C), whose pathogenicity is described as “likely disease-causing but with questionable pathogenicity” in HGMD Professional.

For splicing variant No.1 (c.40-14 A > G), splicing could not be experimentally proven in the minigene system, and therefore, it could not be concluded. Variants No. 22 (c.2341 + 1 G > C) and No. 23 (c.2469 + 2 T > G) exhibited aberrant splicing that was different from the WT; however, the splicing pattern could not be assessed because of unexplained splicing events, such as cryptic exon creation (Supplementary Figure S1).

### *In Silico* Splicing Prediction

The *in silico* splicing prediction algorithms predicted aberrant splicing in all variants, with significant variation scores between the WT and variant. These variants were predicted to disrupt the donor, acceptor, and branch point sites, and create novel donor/acceptor site. The assessment of 25 comparable cases demonstrated consistent splicing patterns between those predicted by the minigene system and SpliceAI-lookup in 16 cases—partially consistent in 4 cases, and inconsistent in 4 cases (Table 2 and Supplementary Figure S1).

**Table 2:** Splice site scores predicted using *in silico* algorithms and consistency between minigene systems and *in silico* prediction

No.	Variant	*In silico* scores using splicing defect prediction tools												Splice effects observed in minigene system	Consistency between minigene systems and SpliceAI-lookup
		GeneSplicer		MaxEntScan		NNsplice		SSF-like		SpliceAI-lookup					
		WT	Var	WT	Var	WT	Var	WT	Var	Acceptor loss	Donnor loss	Acceptor gain	Donnor gain		
1.	c.40-14 A > G	-	-	-	-	-	-	-	-	0.02	0.01	0.02	0.01	NE	NE
2.	c.199+1 G > A	0.67	-	7.52	-	0.85	-	71.19	-	0.83	0.94	0.00	0.00	exon 3 skipping	perfect
3.	c.439+3 A > G	1.23	-	7.64	2.93	0.79	-	71.49	67.13	0.68	0.61	0.00	0.00	exon 6 skipping	perfect
4.	c.560+1 G > A	2.26	-	8.62	-	0.98	-	87.08	-	0.55	0.85	0.00	0.04	cryptic exon inclusion loss of exon 7 fragment exon 7 skipping	partial
5.	c.561-1 G > A	6.15	-	6.45	-	0.92	-	83.40	-	1.00	0.00	0.99	0.00	loss of exon 8 fragment	perfect
6.	c.561-2 A > G	6.15	-	6.45	-	0.92	-	83.40	-	1.00	0.00	0.95	0.00	loss of exon 8 fragment exon 8 skipping	partial
7.	c.723-1 G > A	2.54	-	7.55	-	0.85	-	71.51	-	0.80	0.58	0.10	0.00	exon 9 skipping	perfect
8.	c.824 G > C	-	-	4.20	-	-	-	69.74	56.43	0.74	0.79	0.00	0.01	exon 9 skipping	perfect
9.	c.825-2 A > G	7.86	-	10.42	-	0.99	-	89.45	-	1.00	0.02	0.94	0.09	inclusion of ivs9 to exon 10 loss of exon 10 fragment	partial
10.	c.939+3 A > C	-	-	7.31	-	0.94	-	81.78	71.90	0.00	0.62	0.00	0.78	loss of exon 10 fragment	perfect
11.	c.940-11 G > A	-	-	3.90	-	-	-	66.67	-	0.74	0.00	1.00	0.00	inclusion of ivs10 to exon 11	perfect
12.	c.940-1 G > A	-	-	3.90	-	-	-	66.67	-	0.99	0.00	0.82	0.00	loss of exon 11 fragment	not
13.	c.1244+1 G > C	4.23	-	8.82	-	0.99	-	74.78	-	0.58	0.99	0.00	0.04	exon 12 skipping	perfect
14.	c.1466 G > A	0.56	-	9.5	2.69	0.92	-	82.83	70.69	0.59	0.91	0.00	0.08	exon 14 skipping	perfect
15.	c.1467-2 A > G	7.27	-	7.66	-	0.80	-	91.30	-	0.99	0.18	0.61	0.02	loss of exon 15 fragment exon 15 skipping	partial
16.	c.1467-3 C > G	7.27	-	7.66	-	0.80	-	91.30	80.48	0.99	0.01	0.98	0.00	loss of exon 15 fragment	perfect
17.	c.1602+1 G > A	-	-	2.63	-	0.86	-	70.05	-	0.09	0.99	0.00	0.51	loss of exon 15 fragment exon 15 skipping	not
18.	c.1603-2 A > C	1.03	-	7.29	-	0.91	-	94.13	-	1.00	0.03	0.82	0.01	loss of exon 16 fragment exon 16 skipping	partial
19.	c.1714-2 A > G	6.43	-	8.69	-	0.82	-	83.96	-	1.00	0.12	0.92	0.03	exon 17 skipping	not
20.	c.1879+5 G > A	4.65	-	9.10	1.48	0.97	-	83.50	71.35	0.06	0.92	0.00	0.70	exon 17 skipping	not
21.	c.2256+1 G > A	6.53	-	10.86	-	1.00	-	100.00	-	0.58	1.00	0.01	0.41	exon 20 skipping	perfect
22	c.2341+1 G > C	-	-	-	-	-	-	45.00	-	0.11	0.60	0.00	0.08	inclusion of ivs 21 to exon 21 and loss of exon 22 fragment ?	NE
23	c.2469+2 T > G	2.64	-	8.56	-	0.84	-	85.56	-	0.60	0.96	0.00	0.07	exon 22 skipping ?	NE
24	c.2581 G > A	5.68	1.91	10.53	5.52	0.99	-	81.63	69.49	0.40	0.53	0.00	0.00	exon 23 skipping	perfect
25	c.2581 G > C	5.68	1.99	10.53	6.41	0.99	0.75	81.63	68.32	0.34	0.57	0.00	0.01	exon 23 skipping	perfect
26	c.2581+1 G > A	5.68	-	10.53	-	0.99	-	81.63	-	0.58	0.95	0.00	0.02	exon 23 skipping	perfect
27	c.2581+1 G > C	5.68	-	10.53	-	0.99	-	81.63	-	0.65	0.95	0.00	0.02	exon 23 skipping	perfect
28	c.2581+4 A > G	5.68	4.28	10.53	7.54	0.99	0.40	81.63	71.55	0.20	0.47	0.00	0.00	exon 23 skipping	perfect

NE, not evaluable; Var, variant; WT, wild type.

### Association of Splicing Patterns With Phenotypes

**Figure 2:**
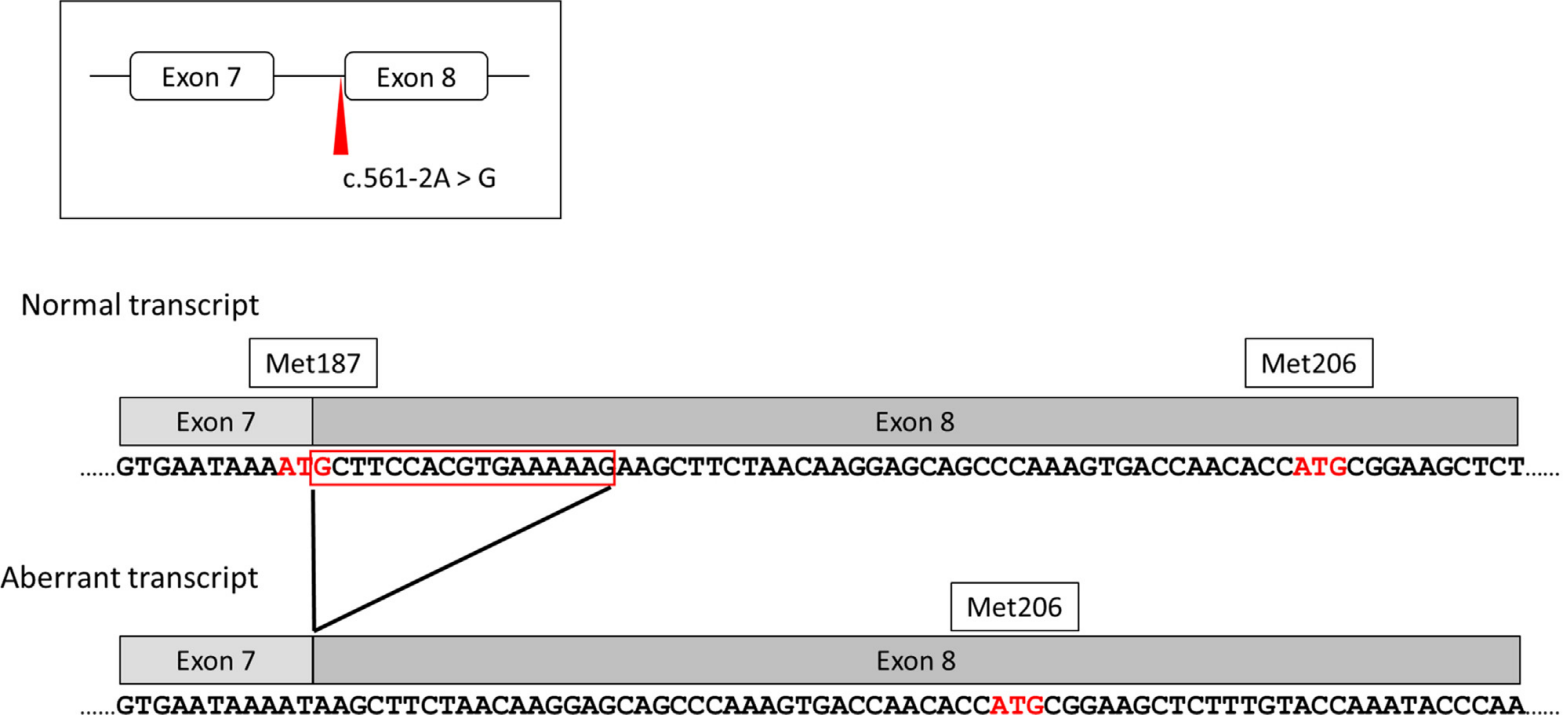
Aberrant splicing and loss of initiation codon observed in c.561-2 A > G. The c. 561-2 A > G variant produced 17 bp deletion, and in this aberrant transcript, the former initiation codon (Met187) is lost. However, the latter initiation codon (Met206) remained, indicating Dent disease-2 phenotype.

According to the HGMD Professional, the phenotype of splicing variants for exons 3, 6, 7, and 8 splice-site was Dent disease-2, and variants for exons 9, 10, 11, 12, 14, 15, 16, 17, 20, 21, 22, and 23 splice-site was Lowe syndrome. Phenotypic data were not available for variant No.5 (c.561-1 G > A). Interestingly, variant No.6 (c.561-2 A > G) in exon 8 exhibited Dent disease-2. Despite the disruption of the former OCRL isoform initiation codon (Met187), the latter OCRL isoform initiation codon (Met206) remained intact, suggesting that isoform rescue was preserved, and the Dent disease-2 phenotype was presented (Figure 2). This result supports our recent discovery of the initiation codons in exon 8.

## Discussion

To the best of our knowledge, this is the first comprehensive study to assess the splicing patterns of *OCRL* splicing variants using the minigene system. In this study, almost all reported splicing variants were confirmed to cause aberrant splicing. In the splicing variant around exon 8, the initiation codon of the OCRL isoform is conserved, leading to Dent disease-2 phenotype. In addition, we compared the minigene results with *in silico* predictions of *OCRL* splicing variants and found that these are quite useful, but not always perfect.

Splicing variants induce splicing errors, which can result in inappropriate intron removal and, in certain cases, null effects. However, confirming the splicing effect requires functional analysis through RNA or protein analysis.^39^ Analysis of mRNA from affected organ tissues is the simplest and most reliable approach to determine the effects of variants on splicing. However, it is difficult to obtain tissue specimens, such as kidneys, to observe tissue-specific gene expression. In addition, this approach is complicated by the instability of mRNAs, specifically aberrant transcripts that are rapidly degraded by nonsense-mediated mRNA decay.^40^ The most straightforward way to determine the pathogenicity of variants at a splice site is through *in silico* analysis. However, it should be noted that these results are only predictions, and the precise effects of variants should be assessed in functional studies.^41^

Splicing assay using a minigene system has been established as an easy and accurate method to identify splicing abnormalities and study their underlying functional mechanisms. When appropriate mRNA sequencing material is unavailable, a minigene system can confirm whether the potential splicing variant causes aberrant splicing. This approach has been assessed in our studies, with results similar to those of *in vivo* and *in vitro* studies.^26^^,^^42^^,^^43^ The *OCRL* variant c. 940-11 G > A demonstrated a similar splicing pattern between the minigene system and mRNA analysis collected from lymphocytes.^26^ Splicing assay using the minigene system can also contribute significantly to the elucidation of pathogenesis. For example, a sibling case with the *OCRL* c.2257-5G > A variant has been reported. The elder brother had an atypical Lowe syndrome with renal symptoms and developmental delay but no ocular involvement, whereas the younger brother had Dent disease-2 with only a renal presentation. This variant causes incomplete alternative splicing, which creates a small amount of WT transcript and a relatively large amount of aberrant splicing transcript with a premature termination codon. This means that these patients exhibited a mild phenotype because of the expression of WT transcript, albeit in low amounts. Surprisingly, aberrant splicing transcript with a premature termination codon could not be detected in the lymphocytes mRNA of patients because of nonsense-mediated mRNA decay mechanism. Indeed, when nonsense-mediated mRNA decay inhibitor was administered to patient lymphocyte mRNA, aberrant splicing transcript with premature termination codon was detected.^44^

Unfortunately, the c.40-14 A > G variant could not be assessed for its pathogenicity using our minigene system. In the original report, this variant demonstrated 2 aberrant splicing events, lacking the 3’ end of exon 1 that contains the initiation codon.^9^ Despite these exceptions, our minigene system is useful as a splicing assay for *OCRL* variants.

In this study, *in silico* analysis predicted that the original splice donor site or acceptor site was destroyed in all cases, except for variant No.1. In addition, in more than half of the cases, SpliceAI-lookup accurately predicted aberrant splicing patterns, including exon skipping, exon loss, and intron inclusion. For example, the minigene system demonstrated that variant No.11 (c.940-11 G > A) resulted in a 9-bp insertion in the intron, which was accurately predicted by the SpliceAI-lookup. In addition, this case demonstrated a similar splicing pattern in patient-derived mRNA.^26^ Although SpliceAI-lookup is a powerful tool for predicting splicing patterns, it has discrepancies with the minigene system in cases, indicating that it cannot yet be solely relied upon.

Cases associated with *OCRL* variants demonstrated that truncating variants in *OCRL* exons 1 to 7 results in Dent disease-2; whereas in 8 to 24, they result in Lowe syndrome, indicating that OCRL isoforms are responsible for differences in disease severity.^9^^,^^10^ In this study, the phenotype of splicing variants for exons 3, 6, 7, and 8 was Dent disease-2; and for exons 9, 10, 11, 12, 14, 15, 16, 17, and 20 was Lowe syndrome. In our recent study, we successfully cloned a novel *OCRL* isoform transcript from exons 6 to 24, with its transcriptional initiation codon in exon 8 (Met187 and Met206), which exhibited a strong function of OCRL protein.^11^ Further, 1 result supported this finding. The variant of the splice site of exon 8, No.6 (c.561-2 A > G), produced an alternative 3' site and exhibited the phenotype of Dent disease -2. In this aberrant splicing pattern, Met187 of OCRL was disrupted, indicating a milder phenotype, such as Dent disease-2. We should focus on the splicing variant around exon 8 and examine the detailed splicing pattern to confirm the effect of 2 initiation codons of the OCRL isoform.

In this study, there were unresolved issues. If the detailed clinical presentation of each case is available, the association between clinical severity and splicing patterns (frameshift or an in-frame variant) should be assessed. In addition, it would be necessary to evaluate the consistency of the splicing analysis using patient-derived samples and minigene system. For variant No.11 (c.940-11 G > A), previous studies have demonstrated identical splicing patterns in the lymphocyte-extracted mRNA and minigene system.^26^ This agreement should be confirmed in multiple cases of *OCRL* variants. Another limitation, which is rather a challenge in the minigene system itself, is that there are rare variants for which splicing cannot be evaluated in the minigene system. In the minigene system, when the consensus (or canonical) sequence at the splice site is noncanonical, exonization may not occur or aberrant exonization may occur. This is exactly the case for No. 1 (c.40-14 A > G) with the variant of the splice site of exon 2; however, the sequence of the splice acceptor site of exon 2 was noncanonical, so that the splicing pattern could not be evaluated.

Despite some unresolved issues, this study offers significant insights into the pathogenicity of *OCRL* splicing variants and the relationship between splicing patterns and phenotypes.

## Disclosure

KN is a member of advisory groups for Kyowa Kirin Co. Ltd., Toa Eiyo Ltd., Zenyaku Kogyo Co. Ltd., and Taisho Pharmaceutical Co. Ltd.; has a patent for developing exon skipping therapy for patients with Alport syndrome; received lecture fee from 10.13039/501100013170Ono Pharmaceutical Co. Ltd., Astellas Pharma Inc., Novo Nordisk Pharmaceuticals Ltd., Alexion Pharmaceuticals, Inc., Sumitomo Pharma Co. Ltd., Sanofi S.A., Otsuka Pharmaceutical Co. Ltd., Daiichi Sankyo Company, Limited, and Miyarisan Pharmaceutical Co. Ltd.; received speakers bureaus grants from Sumitomo Pharma Co. Ltd., Chugai Pharmaceutical Co. Ltd., JCR Pharmaceuticals Co. Ltd., Sanofi S.A., Zenyaku Kogyo Co. Ltd., and Kyowa Kirin Co. Ltd.; and received a grant from Zenyaku Kogyo Co. Ltd. and Torii Pharmaceutical Co. Ltd. All the other authors declared no competing interests.

## Supplementary Material

Supplementary File (PDF) Figure S1. mRNA analysis using the minigene system. The transcriptional result, base sequences, and *in silico* analysis of the *OCRL* splicing variant.

## Footnotes

Supplementary File (PDF)
**Figure S1.** mRNA analysis using the minigene system. The transcriptional result, base sequences, and *in silico* analysis of the *OCRL* splicing variant.
