# Supplementary material for: Comprehensive Splice Pattern Analysis for Previously Reported OCRL Splicing Variants and Their Phenotypic Contributions
Source: Kidney Int Rep. 2025 Mar 3;10(5):1509–17. doi: 10.1016/j.ekir.2025.02.023 (PMC12142645; doi:10.1016/j.ekir.2025.02.023)
Supplement: Supplementary File (PDF) — Figure S1. mRNA analysis using the minigene system. The transcriptional result, base sequences, and in silico analysis of the OCRL splicing variant. [file mmc1.pdf]

Supplementary Figure

1. c.40-14 A > G

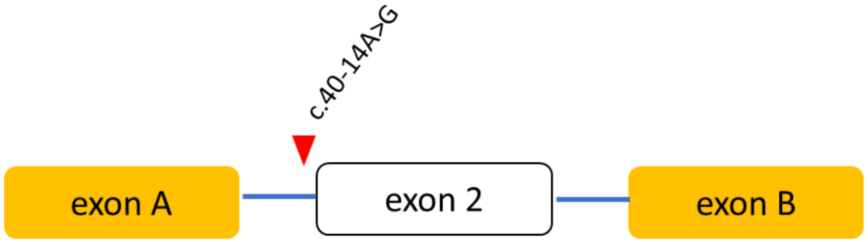

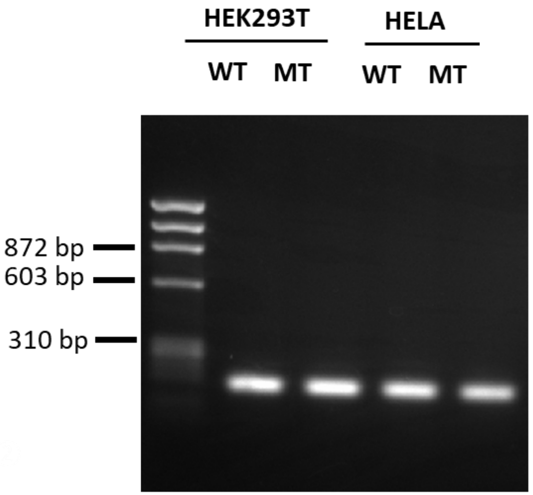

RT-PCR-amplified products of c.40-14A>G hybrid minigene transcripts

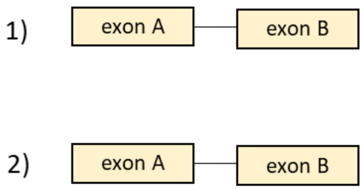

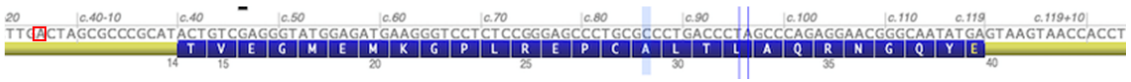

Sequence around *OCRL* Exon 2. Red box shows the site of variant.

c.40-14 A > G band No. 1

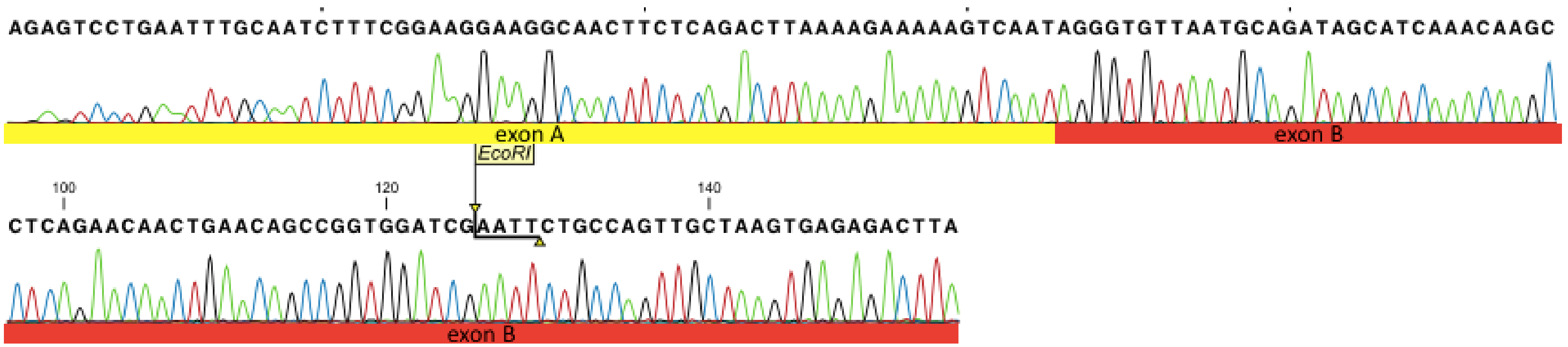

c.40-14 A>G band No. 2

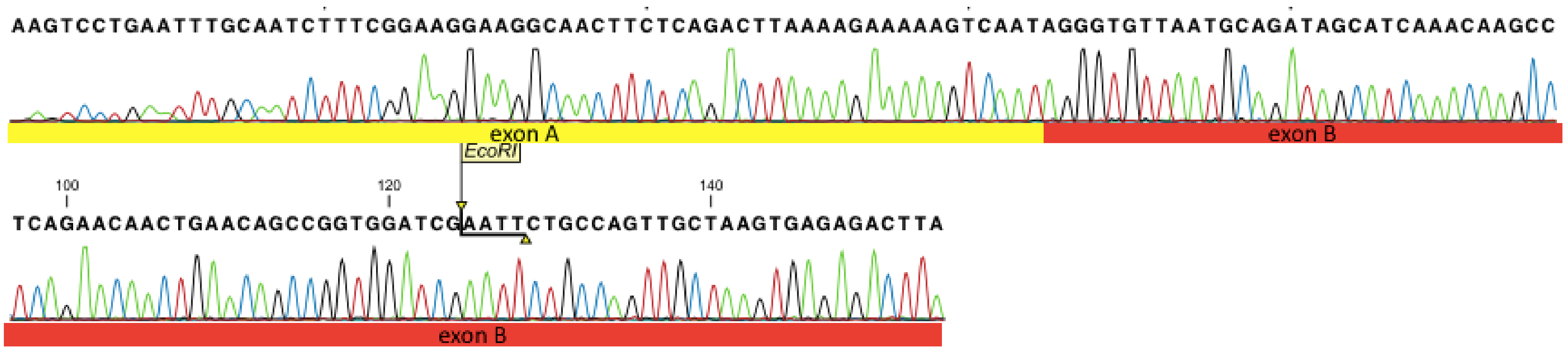

c.40-14 A > G

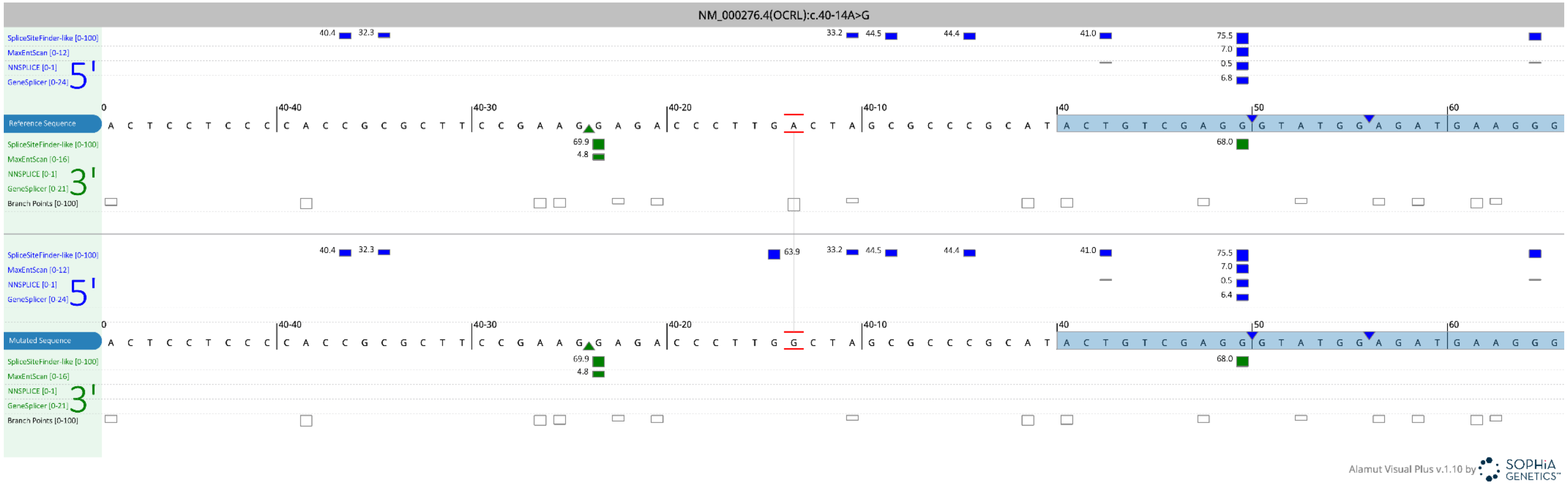

SpliceAI scores: ⓘ

Variant	Gene ☐ = MANE Select transcript ☐ = non-coding transcript	Δ type	Δ score ⓘ	position ⓘ	REF score ⓘ	ALT score ⓘ
NM_000276.4(OCRL):c.40-14A>G<br>⇒ *X:129540730 A>G*<br>UCSC, gnomAD	OCRL<br>(ENSG00000122126.18 / ENST00000371113.9 / NM_000276.4)<br>protein coding MANE Select transcript<br>(plus strand)<br>OMIM, GTEx, gnomAD, ClinGen, Ensembl, Decipher, GeneCards	Acceptor Loss	0.02	-41 bp	0.13	0.11
		Donor Loss	0.01	93 bp	0.91	0.89
		Acceptor Gain	0.02	-10 bp	0.20	0.22
		Donor Gain	0.01	-252 bp	0.52	0.52

2. c.199+1 G > A

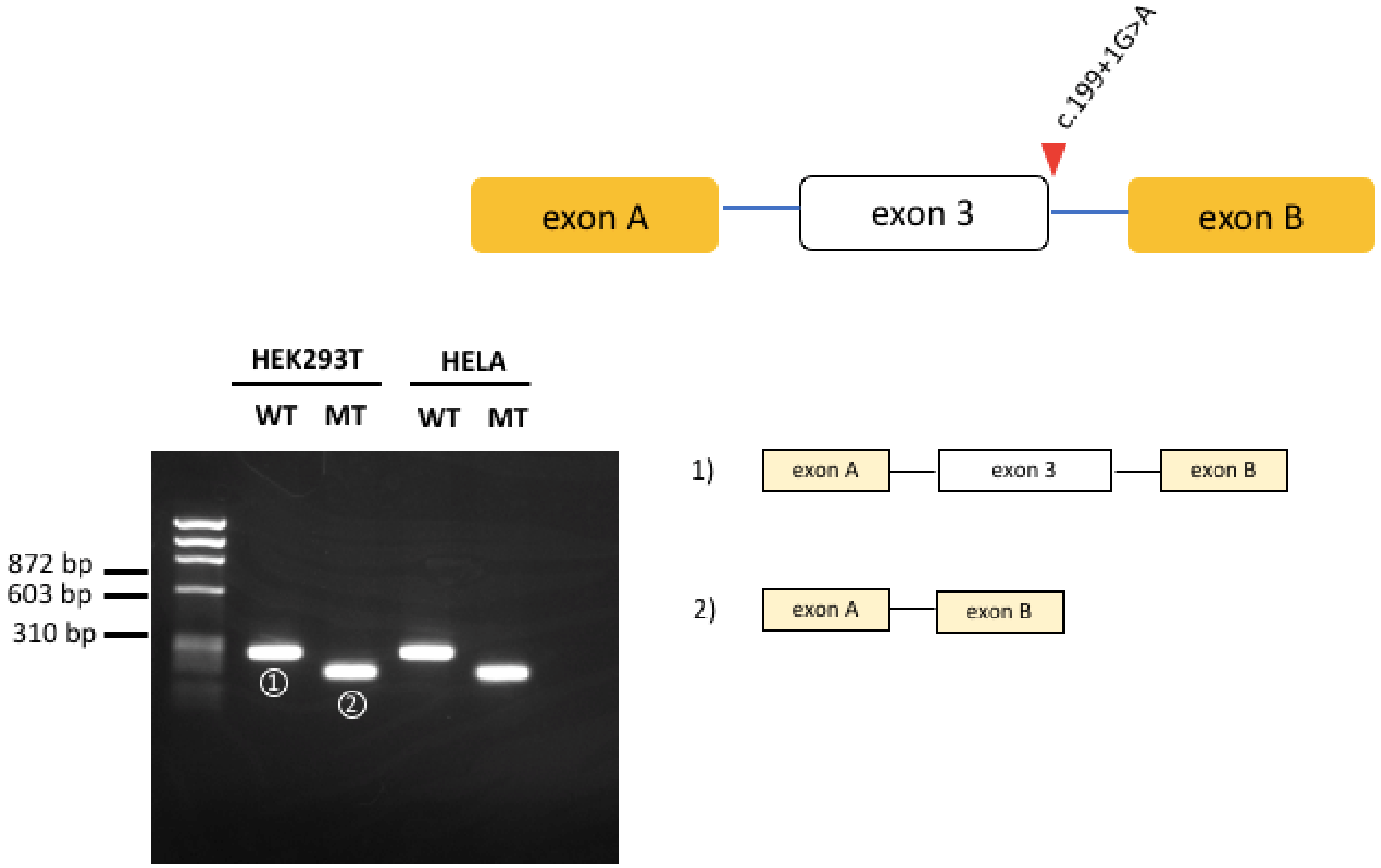

RT-PCR-amplified products of c.199+1G>A hybrid minigene transcripts

c.199+1 G > A band No. 1

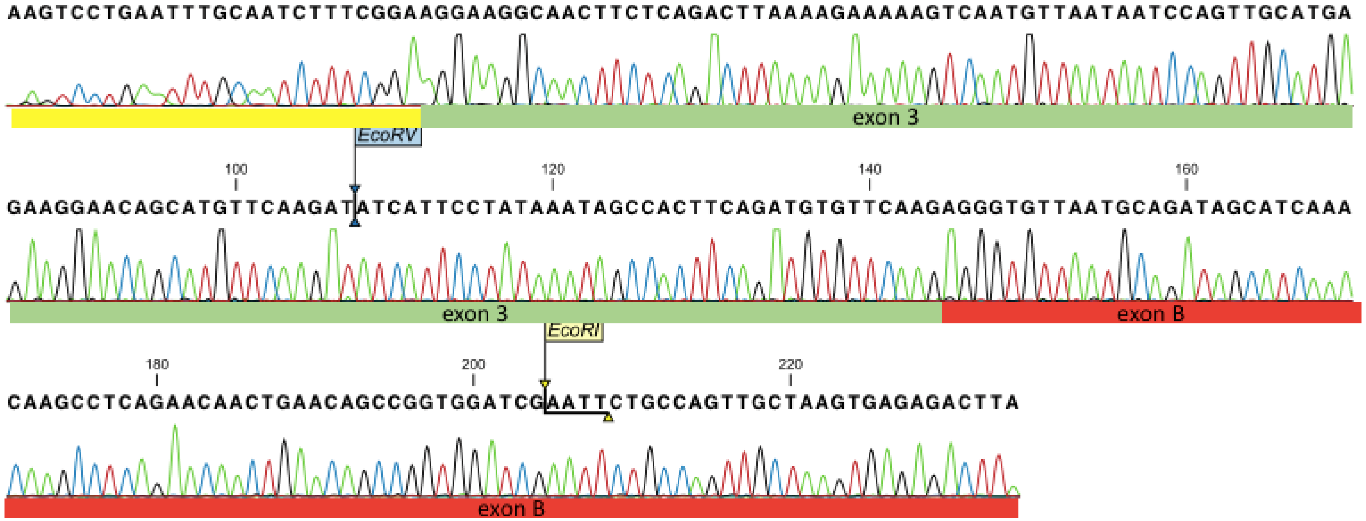

c.199+1 G > A band No. 2

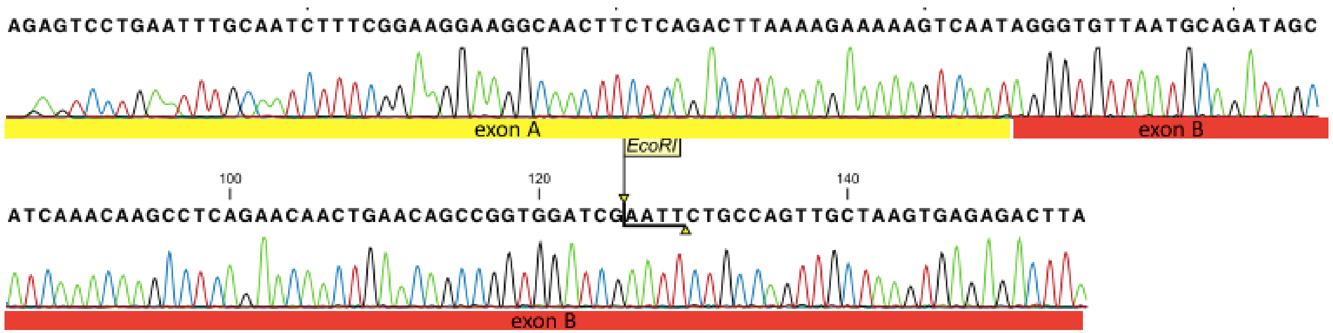

c.199+1 G >A

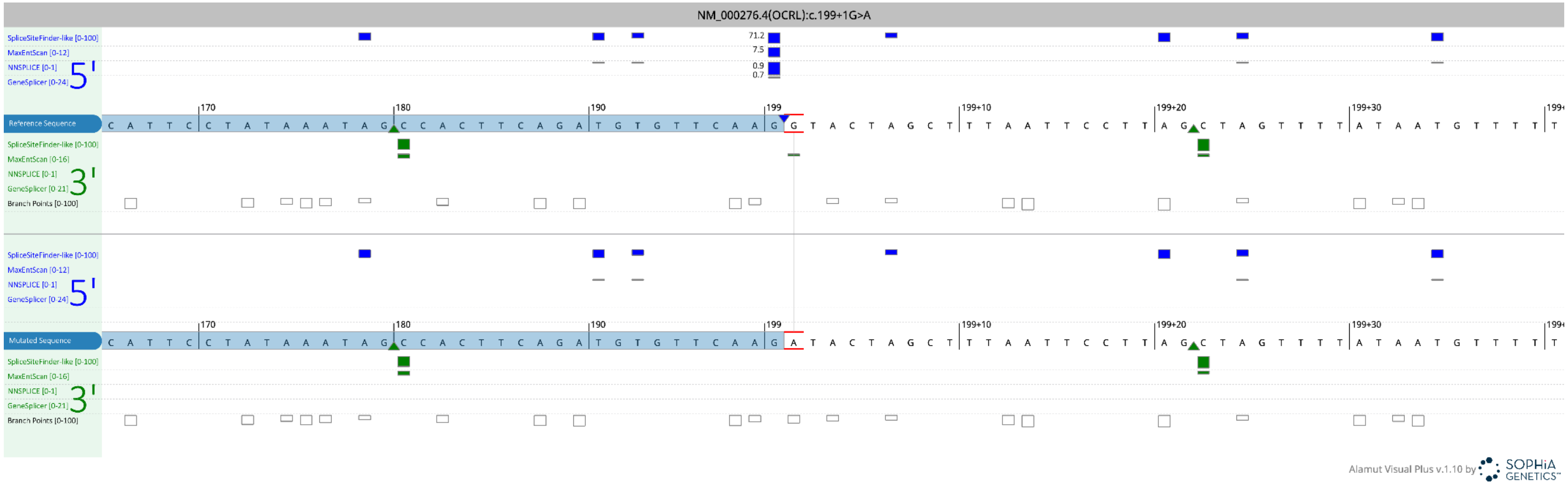

SpliceAI scores:

Variant	Gene ☐ = MANE Select transcript ☐ = non-coding transcript	Δ type	Δ score	position	REF score	ALT score
NM_000276.4(OCRL):c.199+1G>A<br>⇒ *X:129545038 G>A*<br>UCSC, gnomAD	OCRL<br>(ENSG00000122126.18 / ENST00000371113.9 / NM_000276.4)<br>protein coding MANE Select transcript (plus strand)<br>OMIM, GTEx, gnomAD, ClinGen, Ensembl, Decipher, GeneCards	Acceptor Loss	0.83	-80 bp	0.85	0.02
		Donor Loss	0.94	-1 bp	0.94	0.00
		Acceptor Gain	0.00		0.00	0.00
		Donor Gain	0.00		0.00	0.00

3. c.439+3 A > G

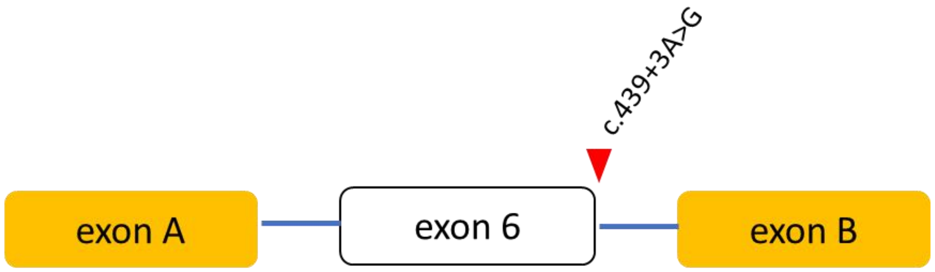

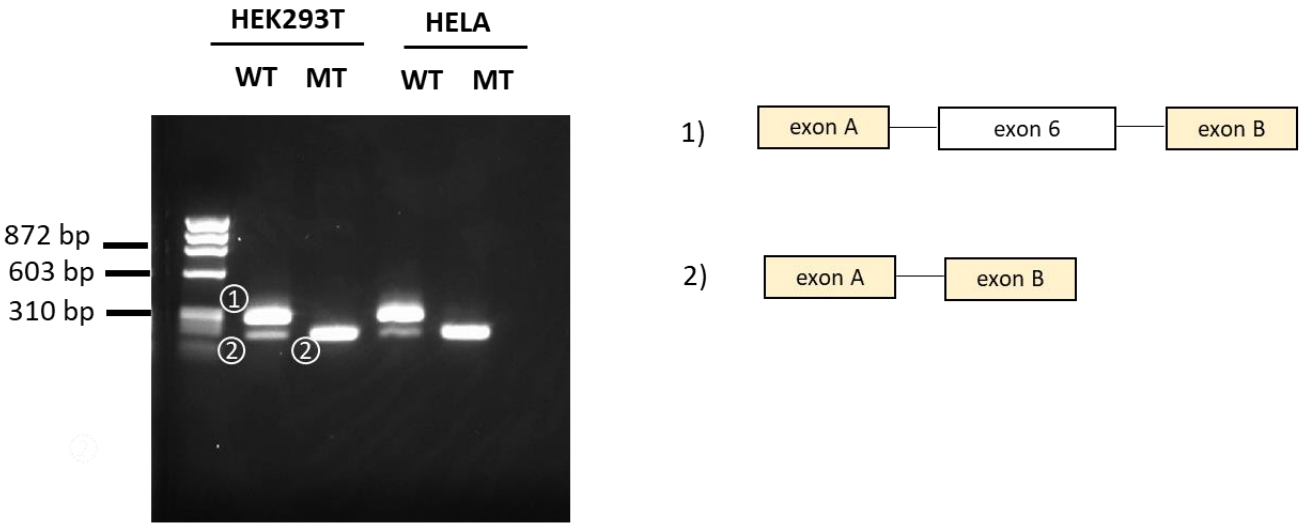

RT-PCR-amplified products of c.439+3A>G hybrid minigene transcripts

c.439+3 A > G band No. 1

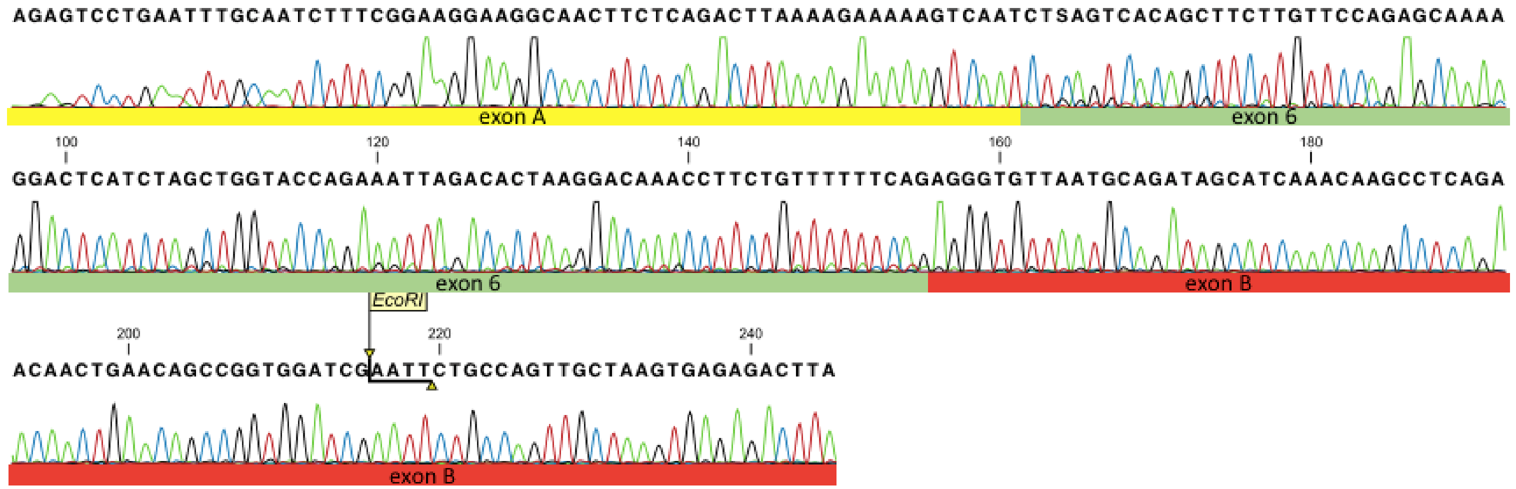

c.439+3 A > G band No. 2

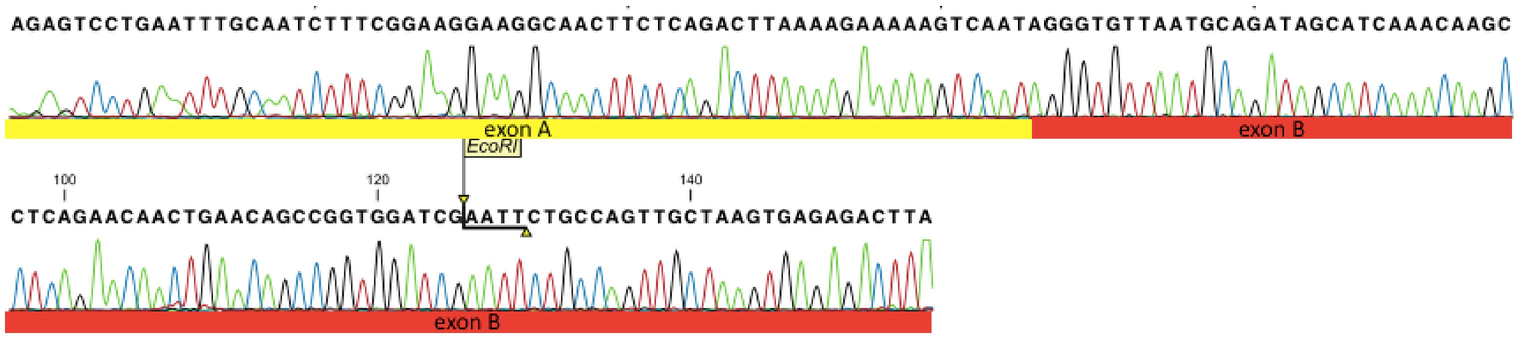

c.439+3 A>G band No. 3

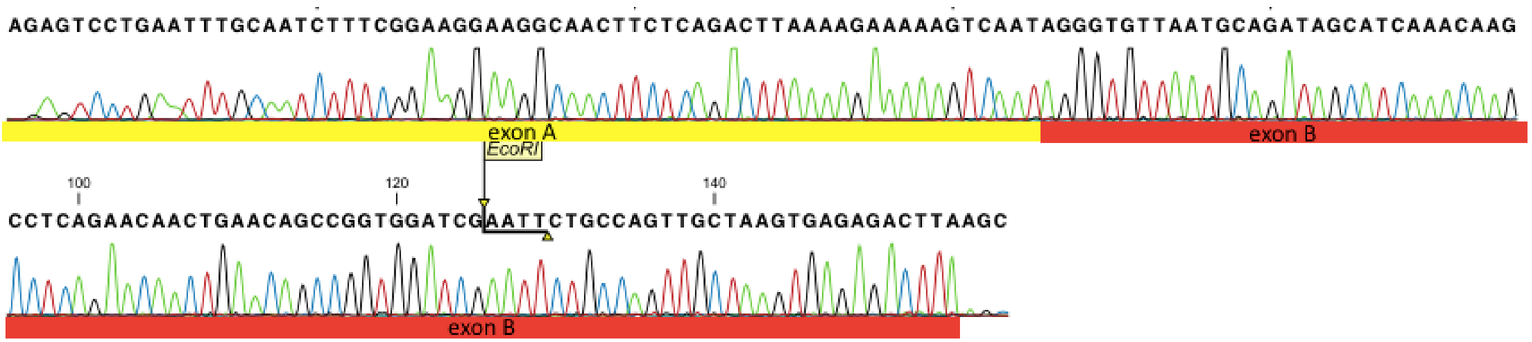

c.439+3 A>G

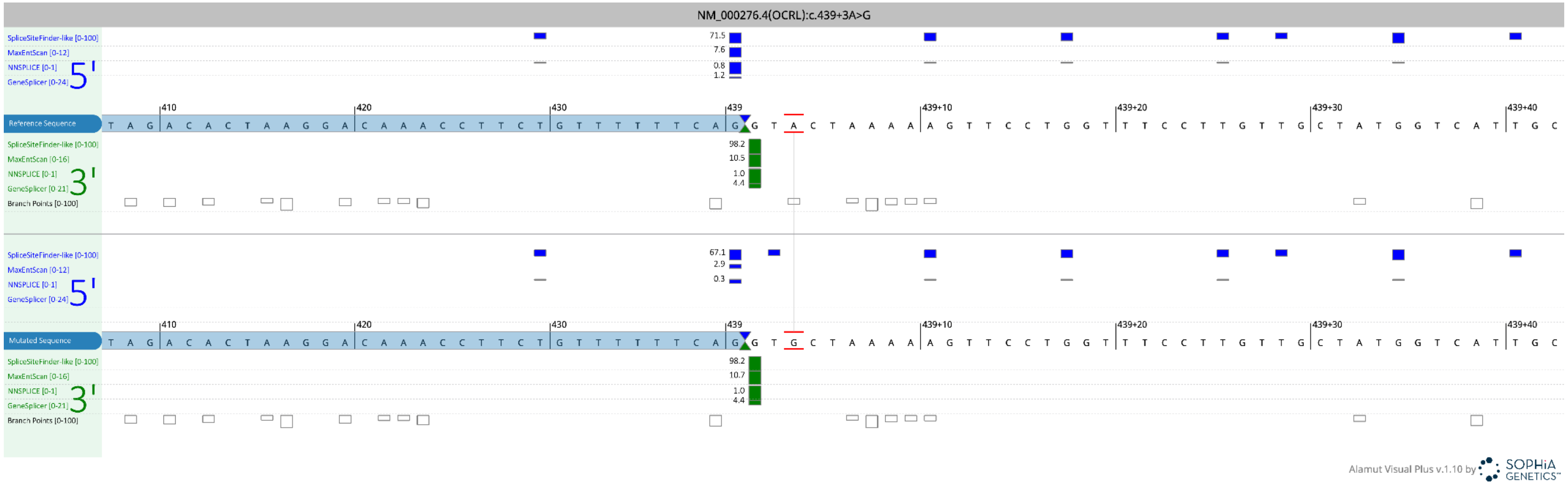

SpliceAI scores:

Variant	Gene (= MANE Select transcript; = non-coding transcript)	Δ type	Δ score	position	REF score	ALT score
NM_000276.4(OCRL):c.439+3A>G<br>⇒ X:129557953 A>G<br>UCSC, gnomAD	OCRL<br>(ENSG00000122126.18 / ENST00000371113.9 / NM_000276.4)<br>protein coding MANE Select transcript (plus strand)<br>OMIM, GTEx, gnomAD, ClinGen, Ensembl, Decipher, GeneCards	Acceptor Loss	0.68	-92 bp	0.74	0.06
		Donor Loss	0.61	-3 bp	0.79	0.18
		Acceptor Gain	0.00		0.00	0.00
		Donor Gain	0.00		0.00	0.00

4. c.560+1 G > A

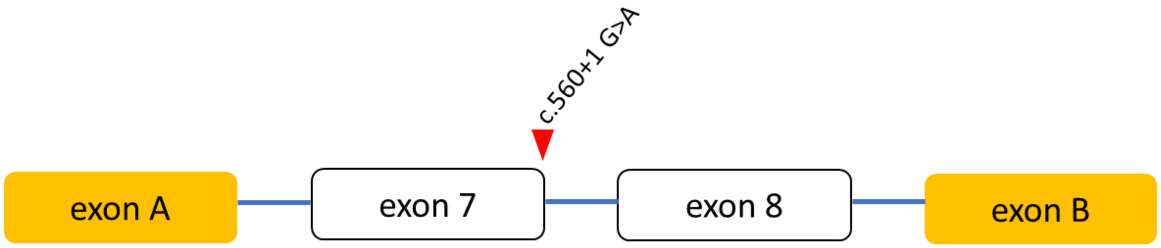

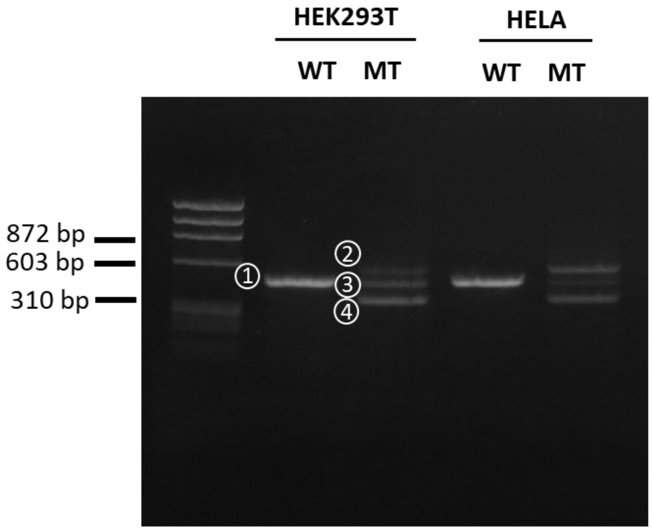

RT-PCR-amplified products of c.560+1G>A hybrid minigene transcripts

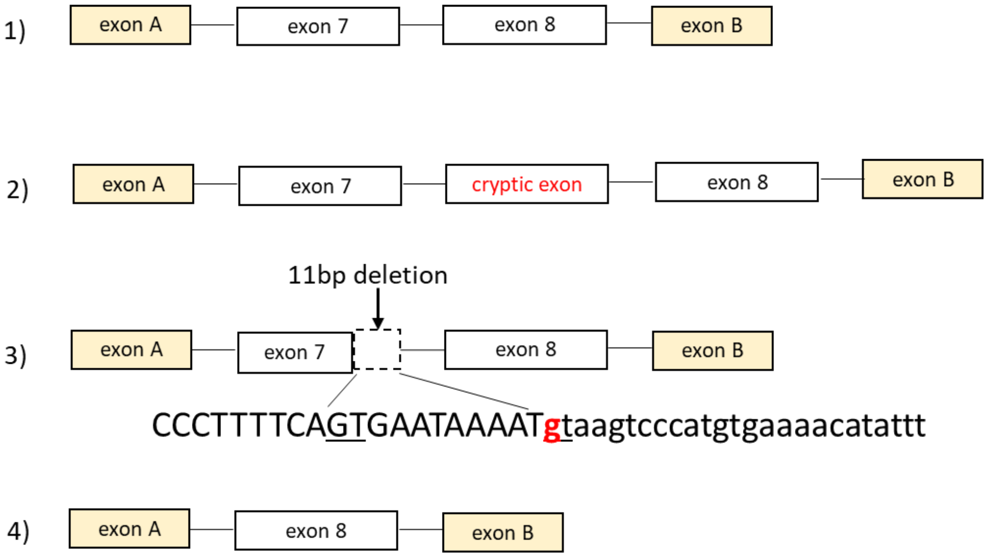

c.560+1 G > A band No. 1

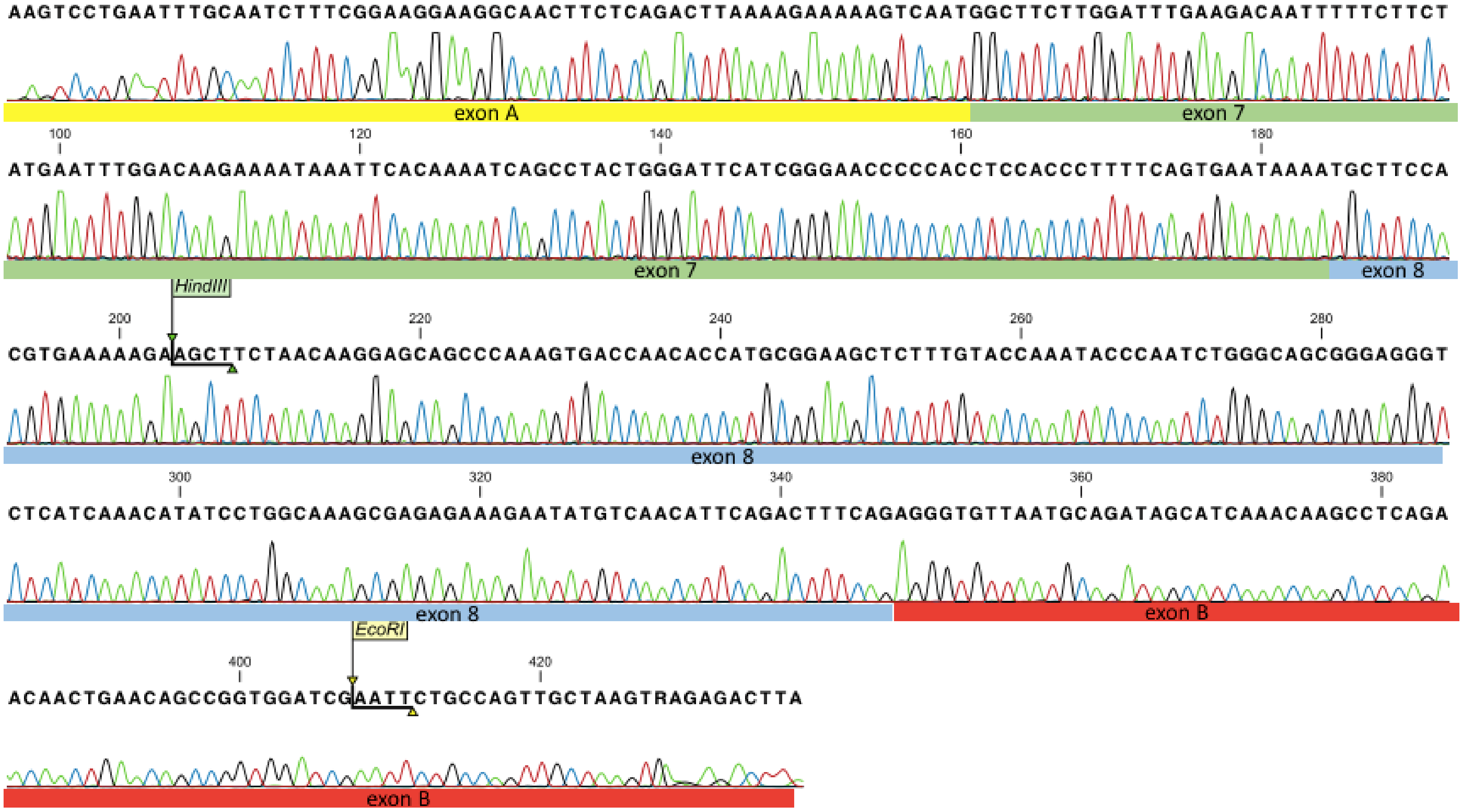

c.560+1 G > A band No. 2

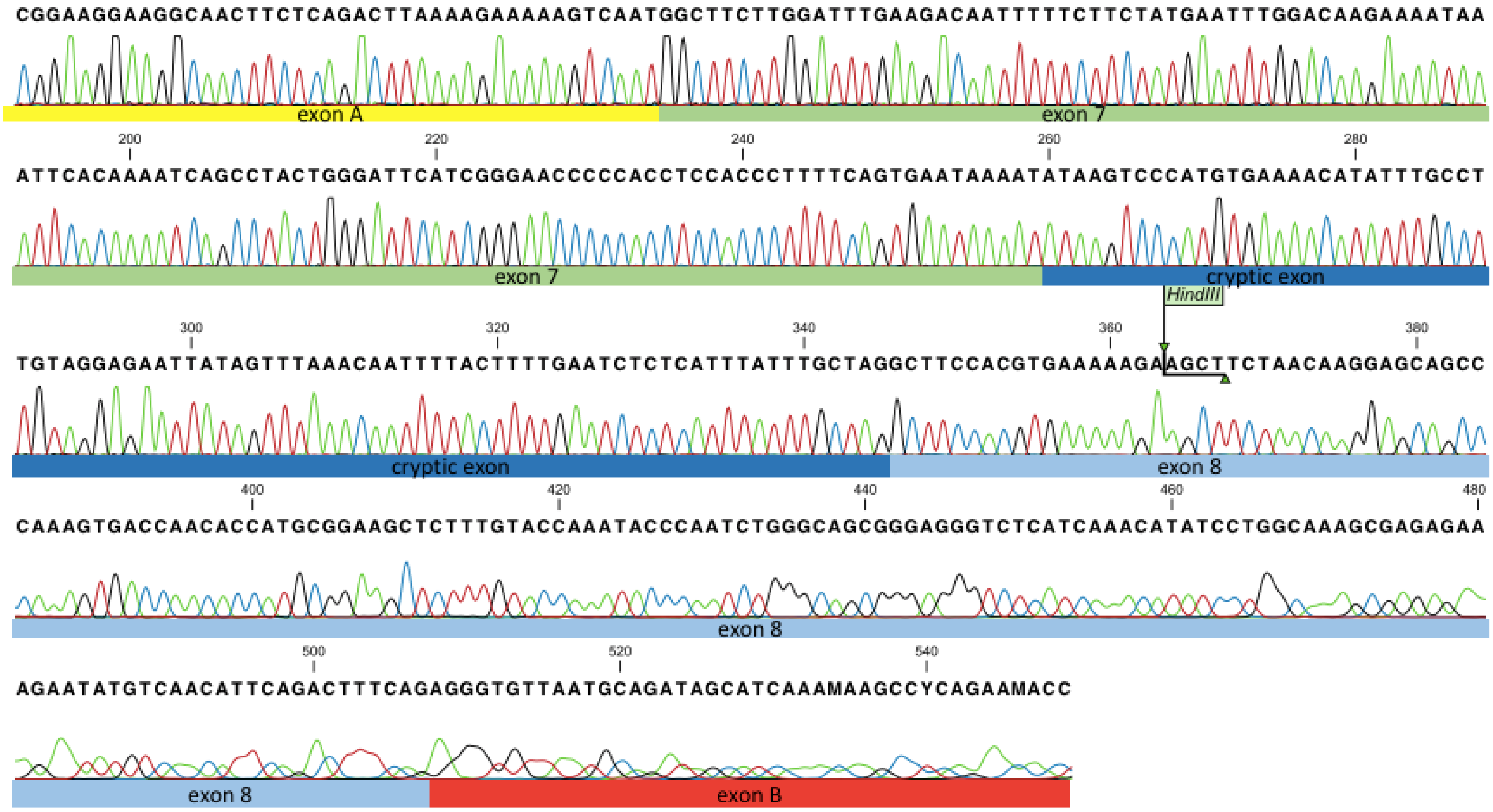

c.560+1 G > A band No. 3

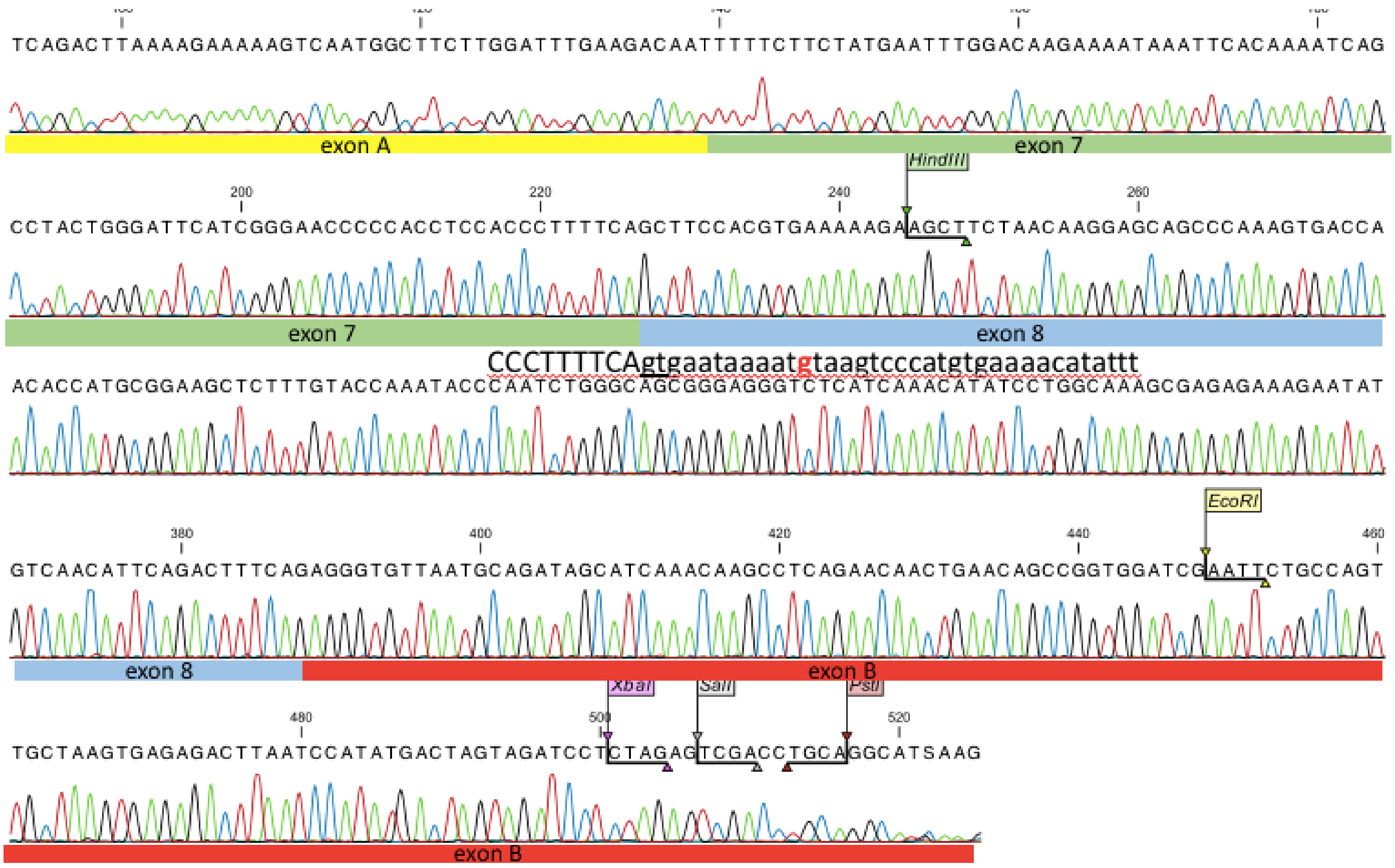

c.560+1 G > A band No. 4

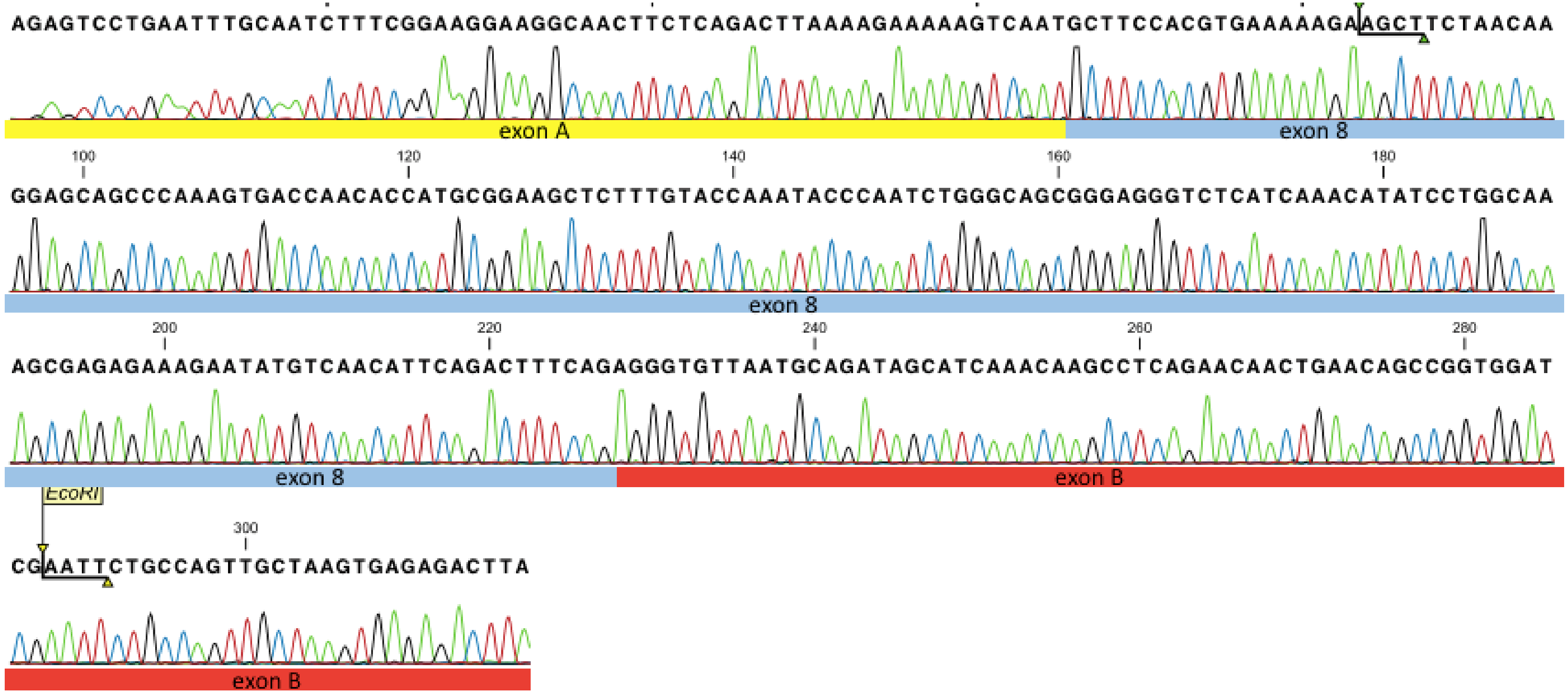

c.560+1 G >A

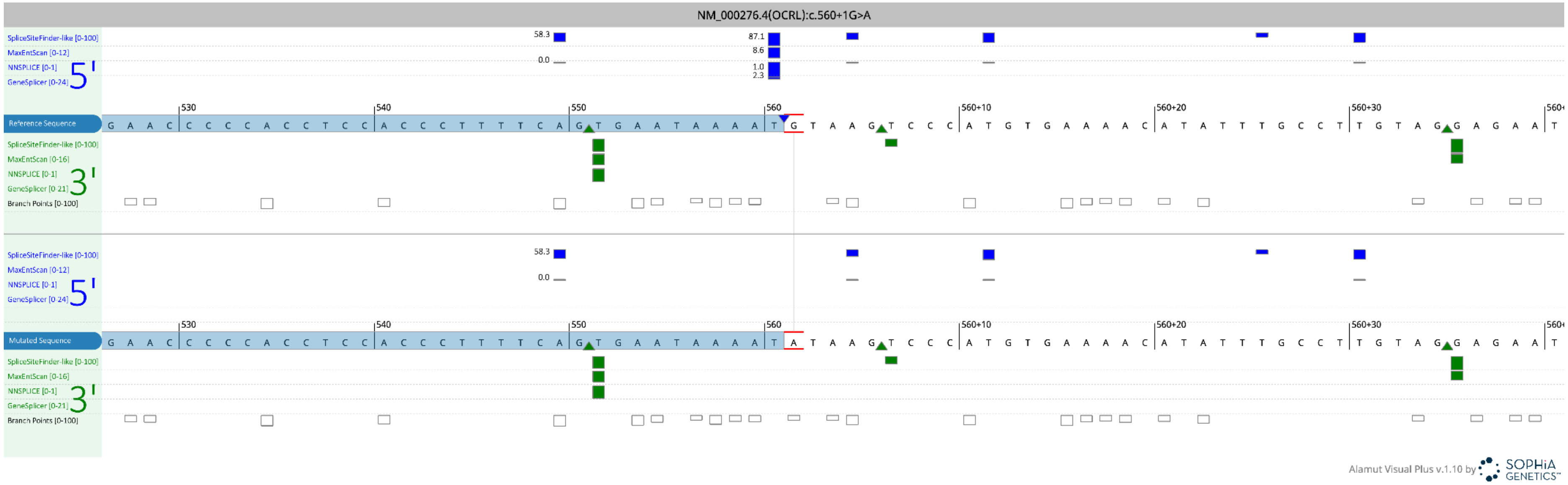

SpliceAI scores:

Variant	Gene (= MANE Select transcript; = non-coding transcript)	Δ type	Δ score	position	REF score	ALT score
NM_000276.4(OCRL):c.560+1G>A<br>⇒ *X:129558754 G>A*<br>UCSC, gnomAD	OCRL<br>(ENSG00000122126.18 / ENST00000371113.9 / NM_000276.4)<br>protein coding MANE Select transcript (plus strand)<br>OMIM, GTEx, gnomAD, ClinGen, Ensembl, Decipher, GeneCards	Acceptor Loss	0.55	-121 bp	1.00	0.44
		Donor Loss	0.85	-1 bp	1.00	0.15
		Acceptor Gain	0.00		0.00	0.00
		Donor Gain	0.04	-12 bp	0.00	0.04

5. c.561-1 G > A

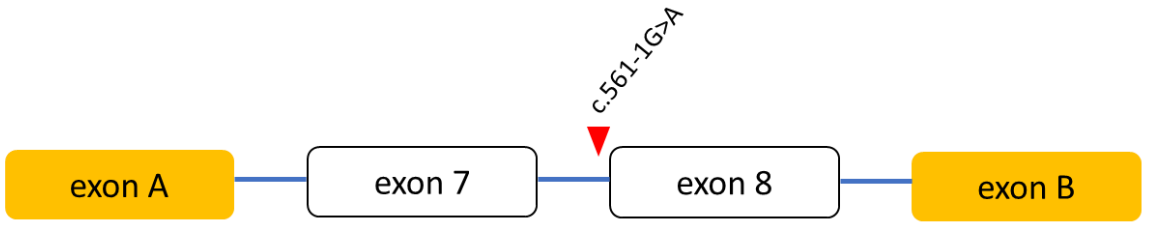

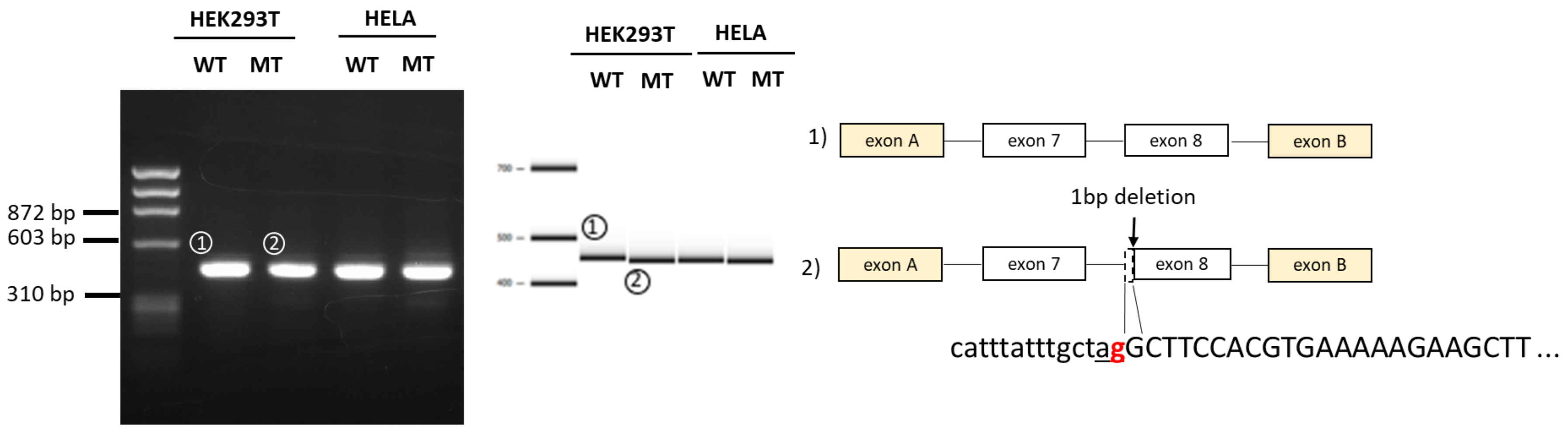

RT-PCR-amplified products of c.561-1G>A
hybrid minigene transcripts (left :electrophoresis; right: DNA1000 assay)

c.561-1 G > A band No. 1

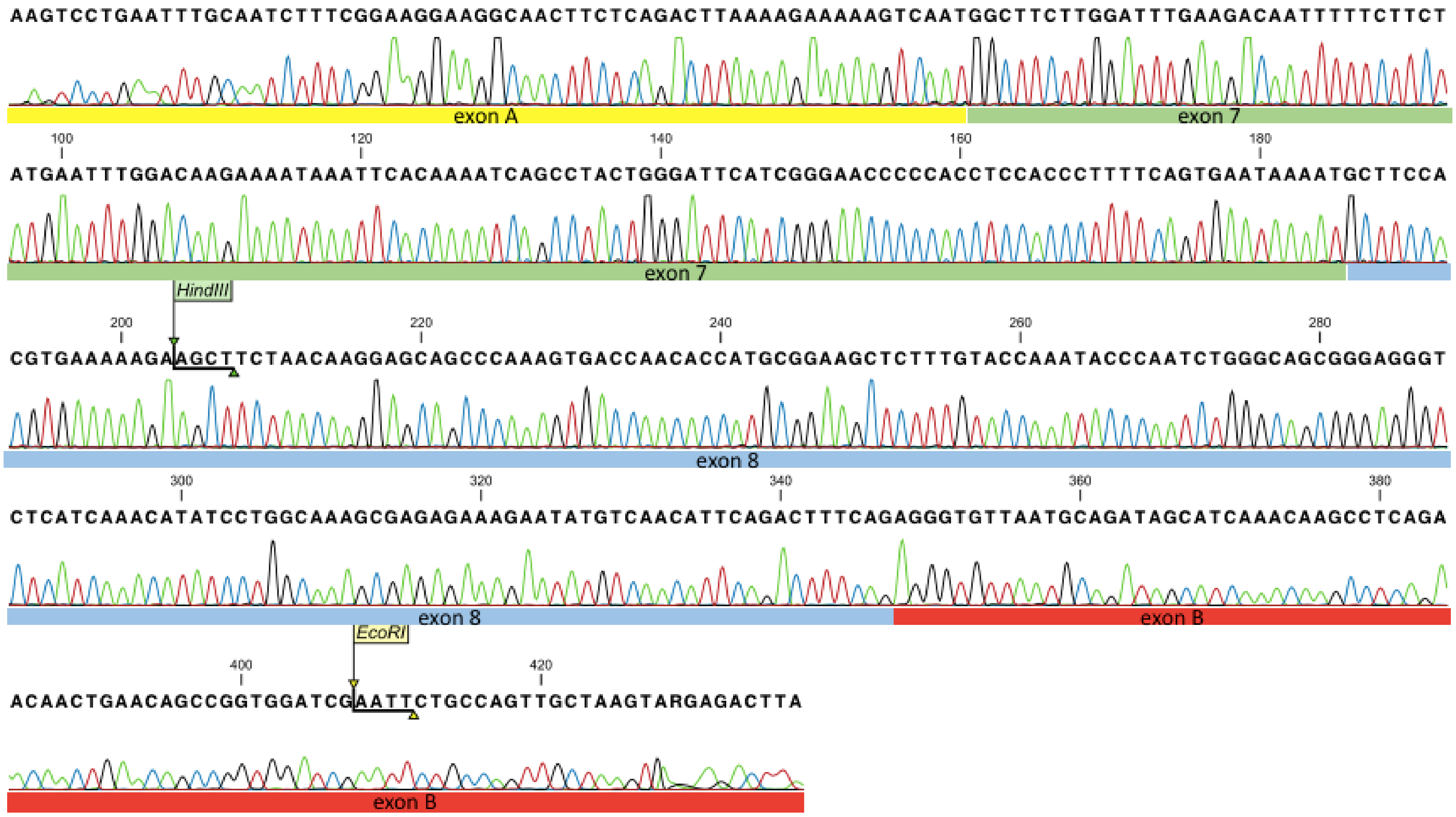

c.561-1 G > A band No. 2

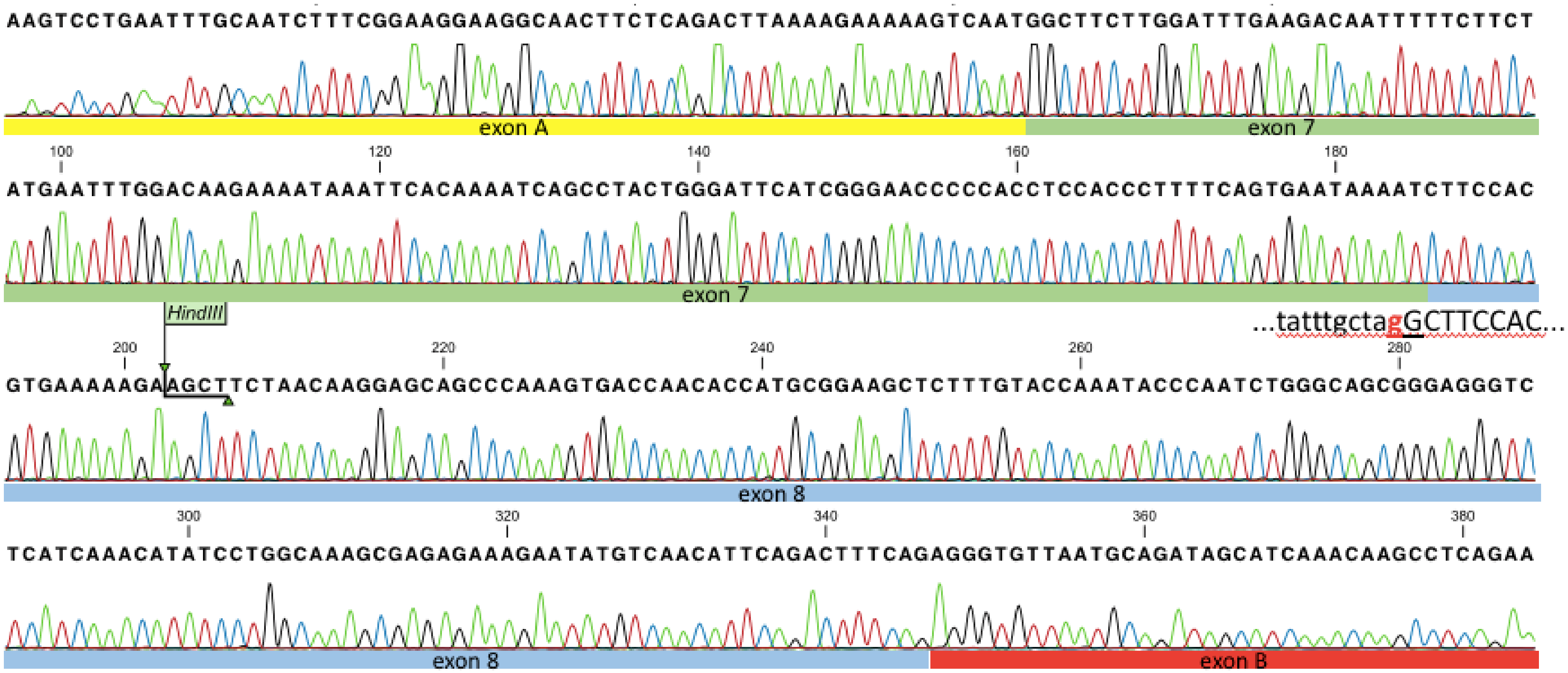

c.561-1 G>A

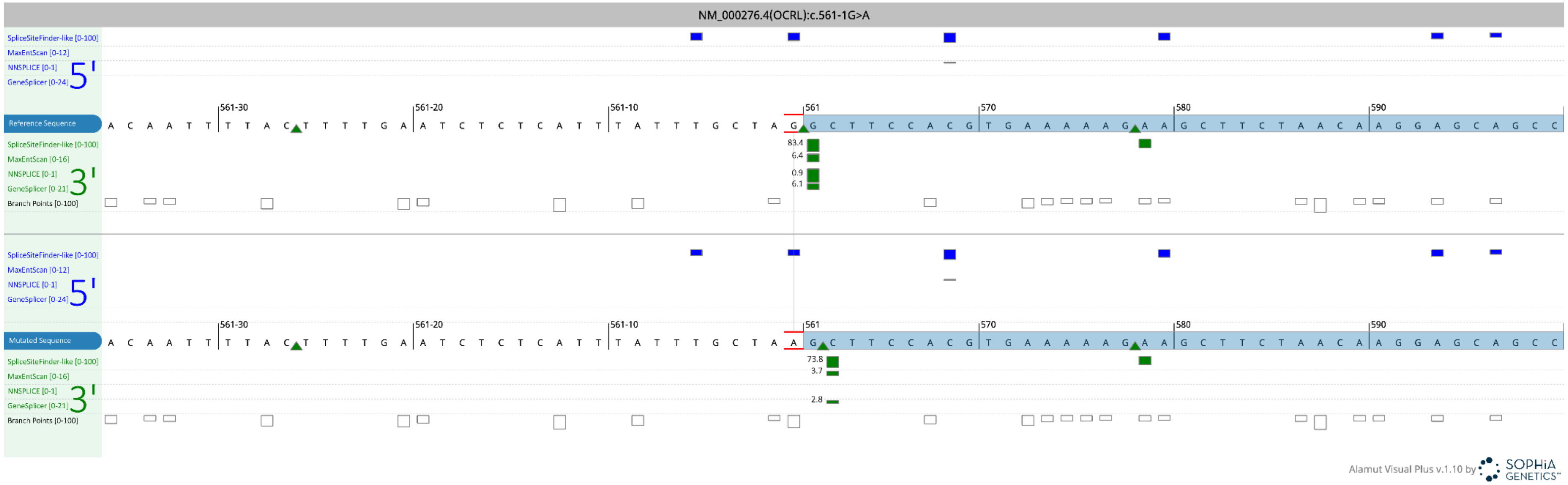

SpliceAI scores:

Variant	Gene ☐ = MANE Select transcript ☐ = non-coding transcript	Δ type	Δ score	position	REF score	ALT score
NM_000276.4(OCRL):c.561-1G>A ⇒ X:129558839 G>A UCSC, gnomAD	OCRL (ENSG00000122126.18 / ENST00000371113.9 / NM_000276.4) protein coding MANE Select transcript (plus strand) OMIM, GTEx, gnomAD, ClinGen, Ensembl, Decipher, GeneCards	Acceptor Loss	1.00	1 bp	1.00	0.00
		Donor Loss	0.00	-86 bp	1.00	1.00
		Acceptor Gain	0.99	2 bp	0.00	1.00
		Donor Gain	0.00		0.00	0.00

6. c.561-2 A > G

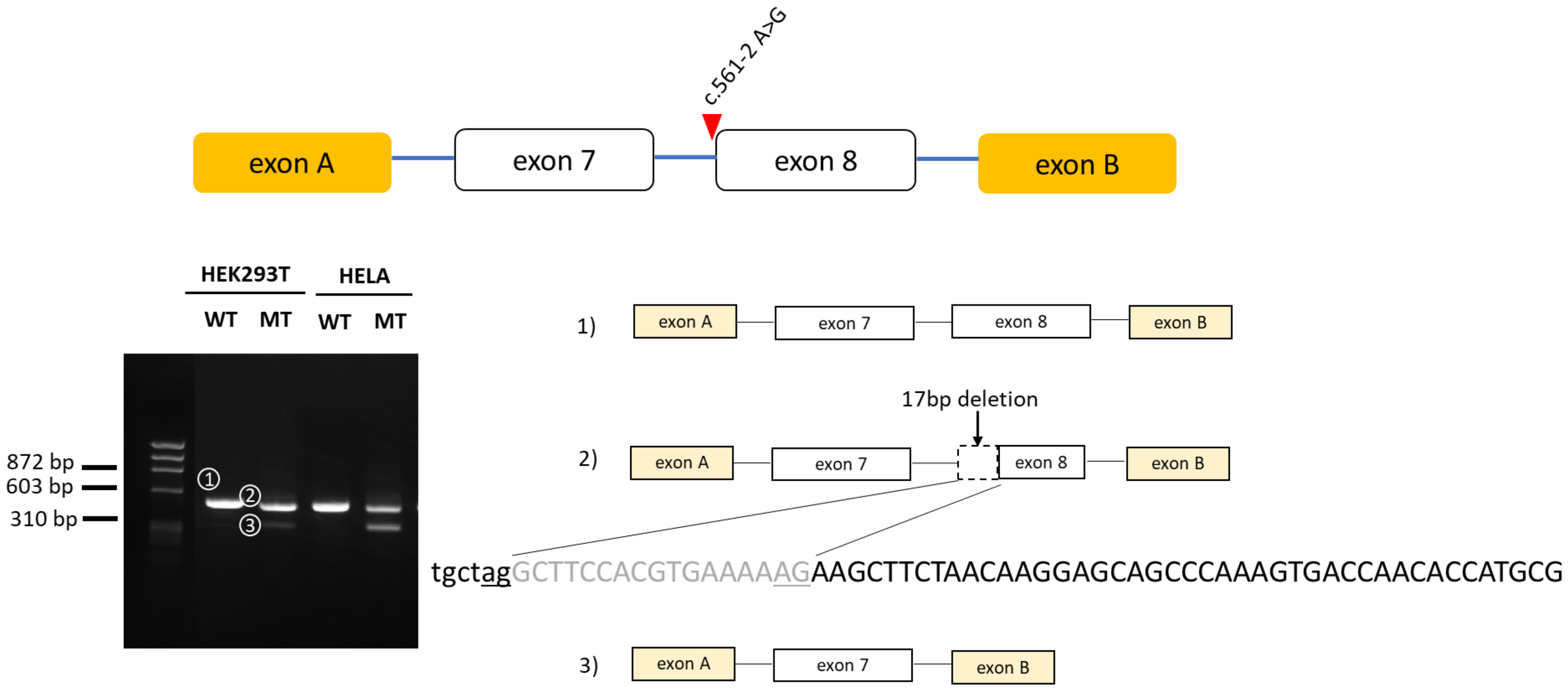

RT-PCR-amplified products of c.561-2A>G hybrid minigene transcripts

c.561-2 A > G band No. 1

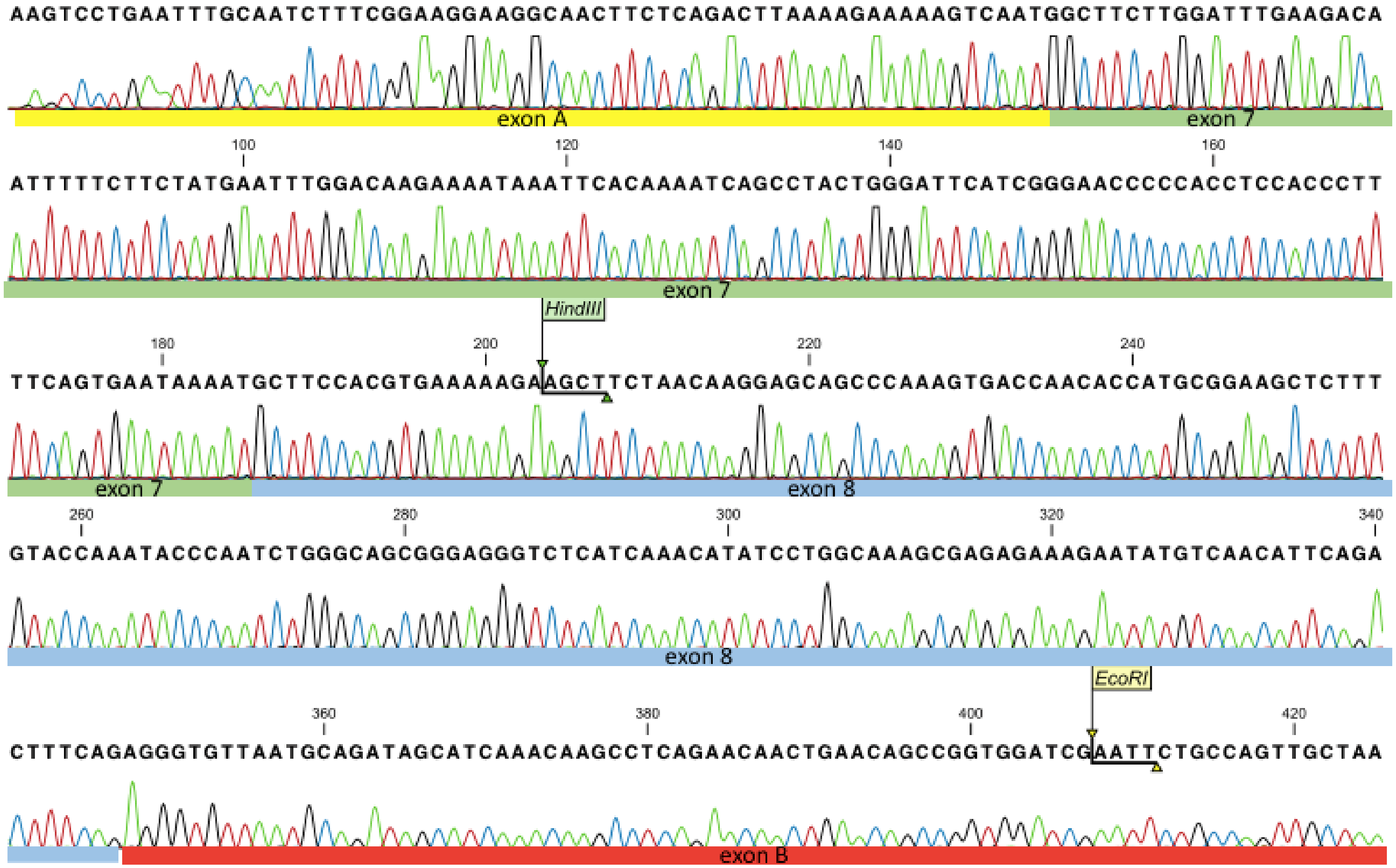

c.561-2 A > G band No. 2

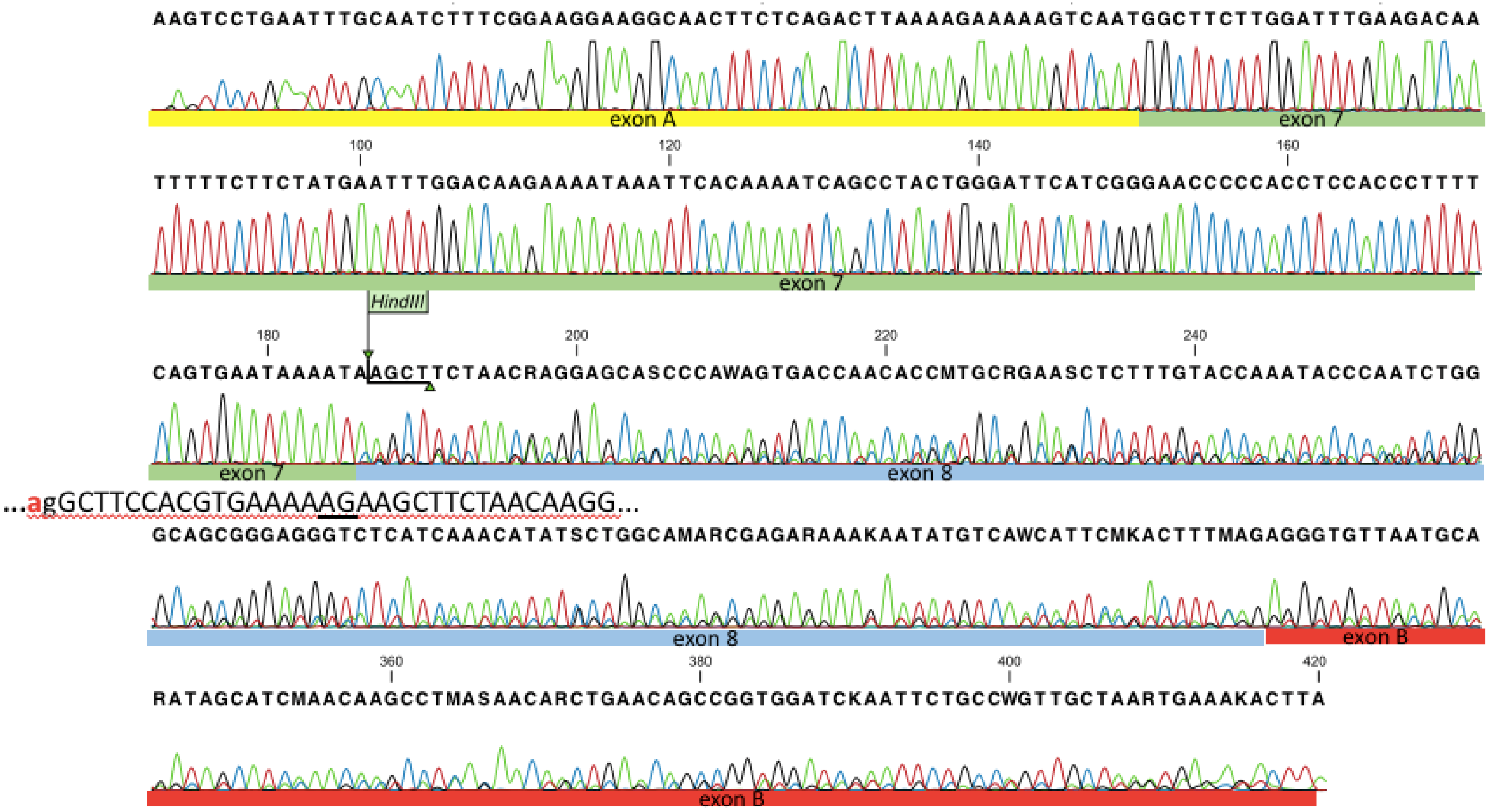

c.561-2 A > G band No. 3

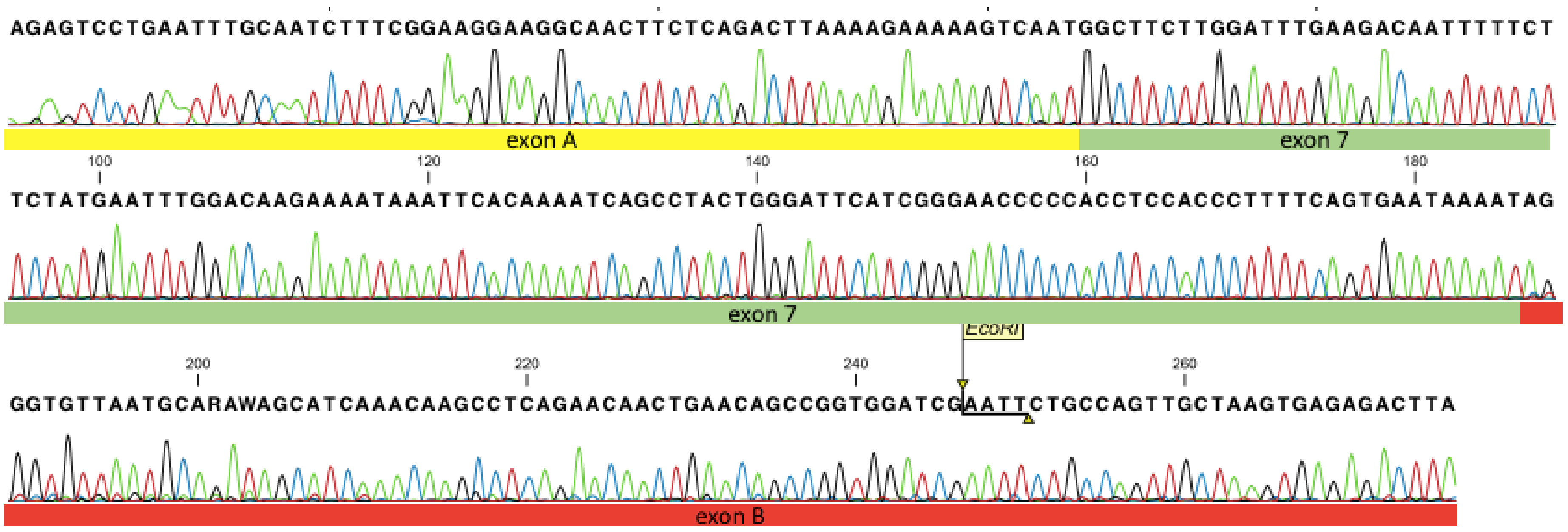

c.561-2 A > G

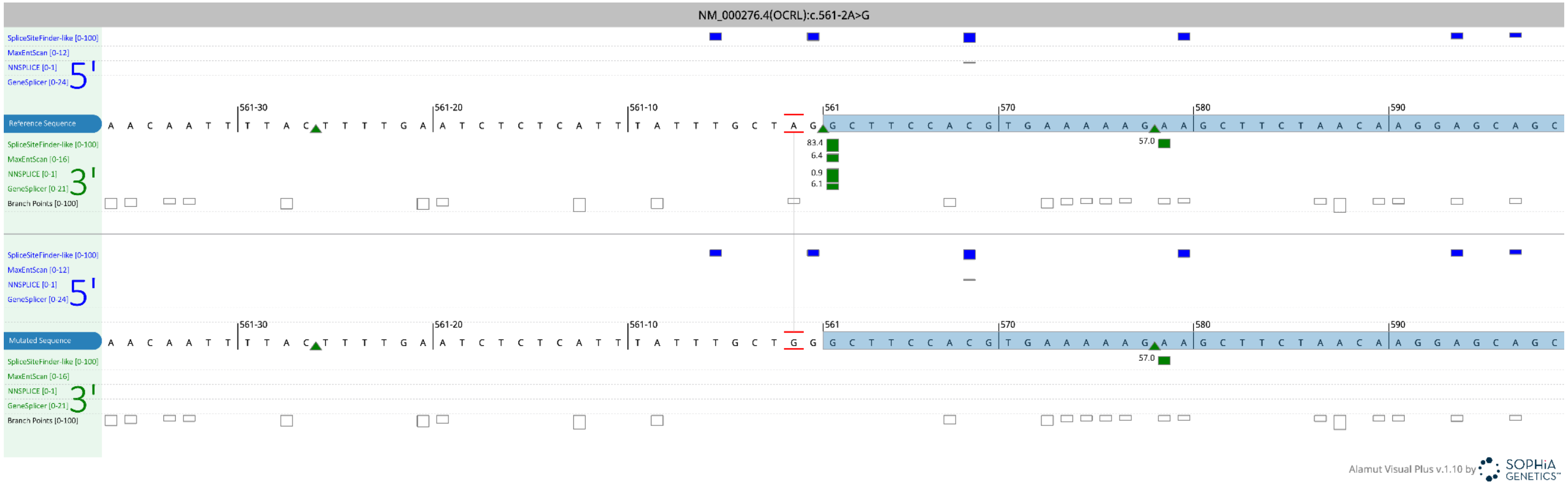

SpliceAI scores: ⓘ

Variant	Gene ☐ = MANE Select transcript ☐ = non-coding transcript	Δ type	Δ score ⓘ	position ⓘ	REF score ⓘ	ALT score ⓘ
NM_000276.4(OCRL):c.561-2A>G<br>⇒ *X:129558838 A>G*<br>UCSC, gnomAD	OCRL<br>(ENSG00000122126.18 / ENST00000371113.9 / NM_000276.4)<br>protein coding MANE Select transcript (plus strand)<br>OMIM, GTEx, gnomAD, ClinGen, Ensembl, Decipher, GeneCards	Acceptor Loss	1.00	2 bp	1.00	0.00
		Donor Loss	0.00	-85 bp	1.00	1.00
		Acceptor Gain	0.95	19 bp	0.00	0.96
		Donor Gain	0.00		0.00	0.00

7. c.723-1 G>A

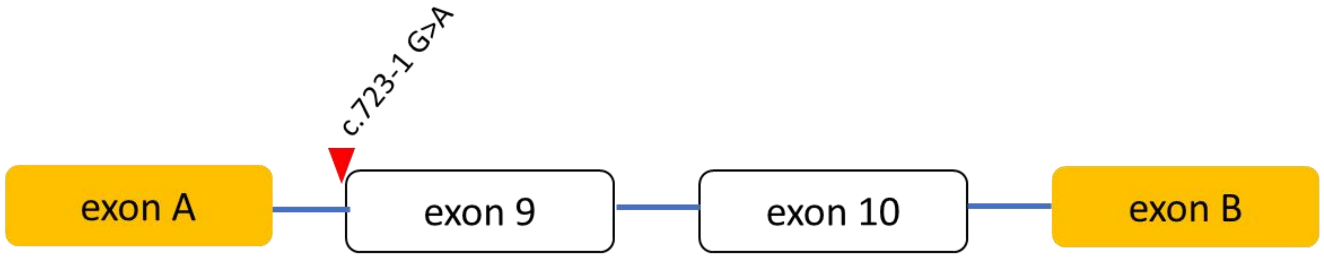

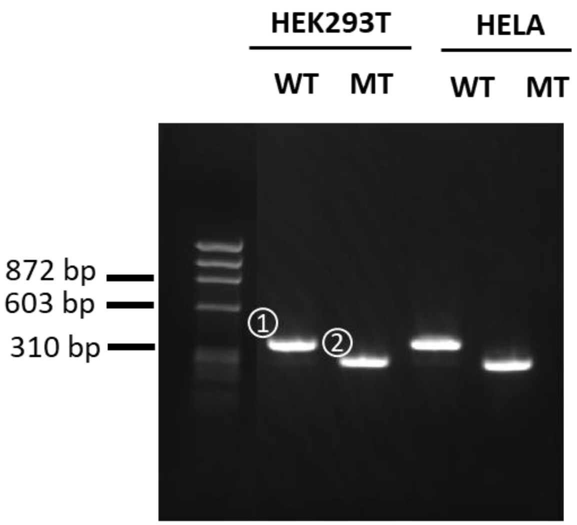

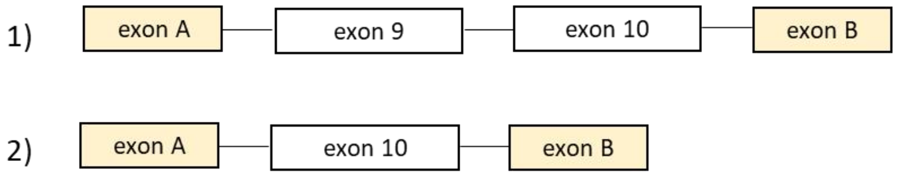

RT-PCR-amplified products of c.723-1G>A hybrid minigene transcripts

c.723-1 G > A band No. 1

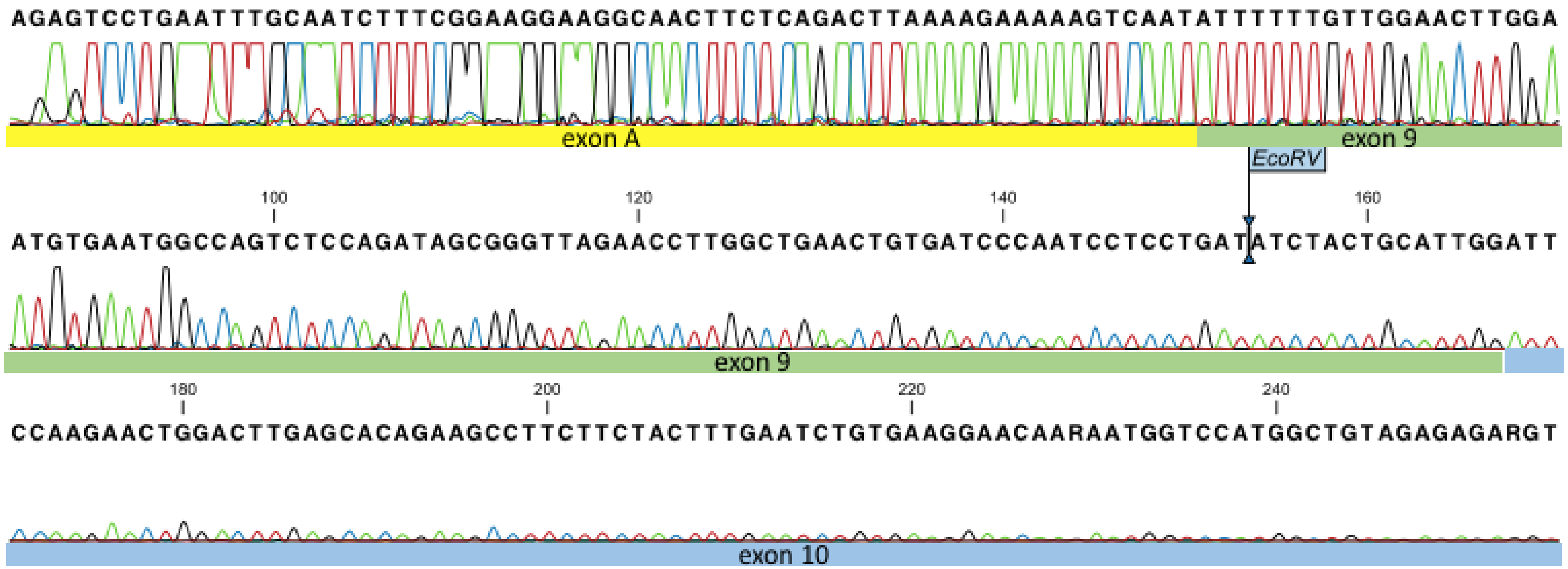

c.723-1 G > A band No. 2

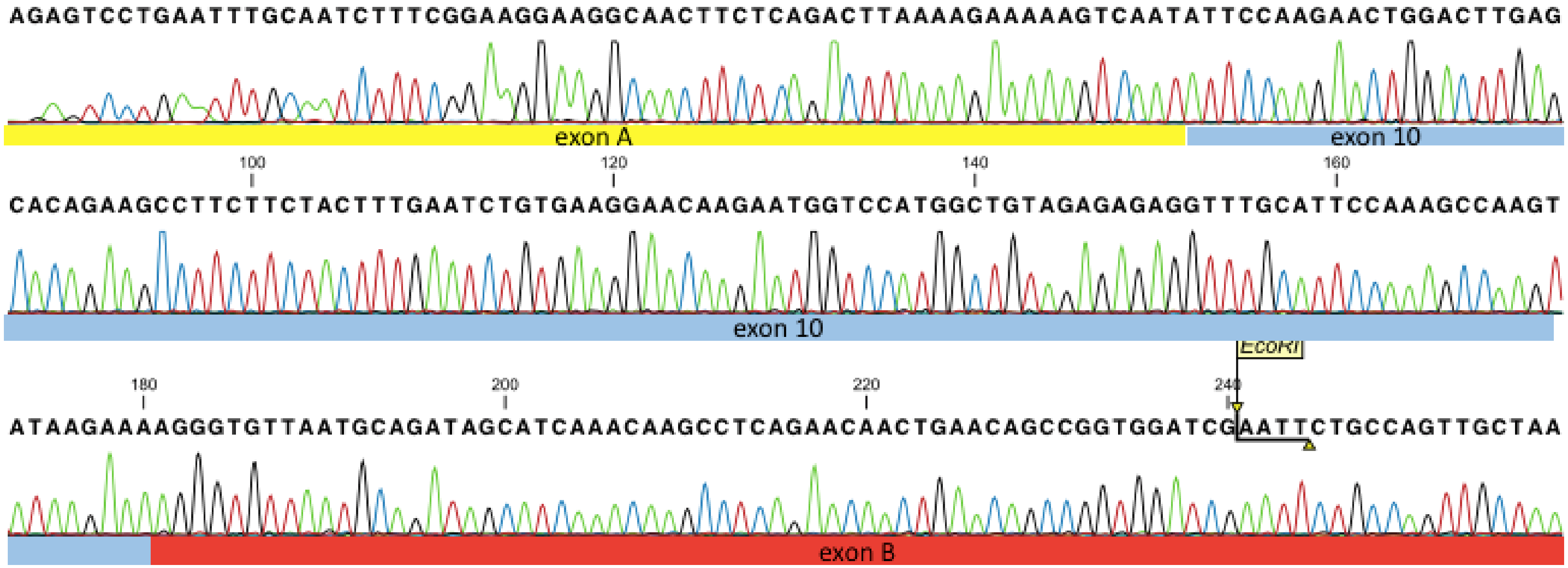

c.723-1 G > A

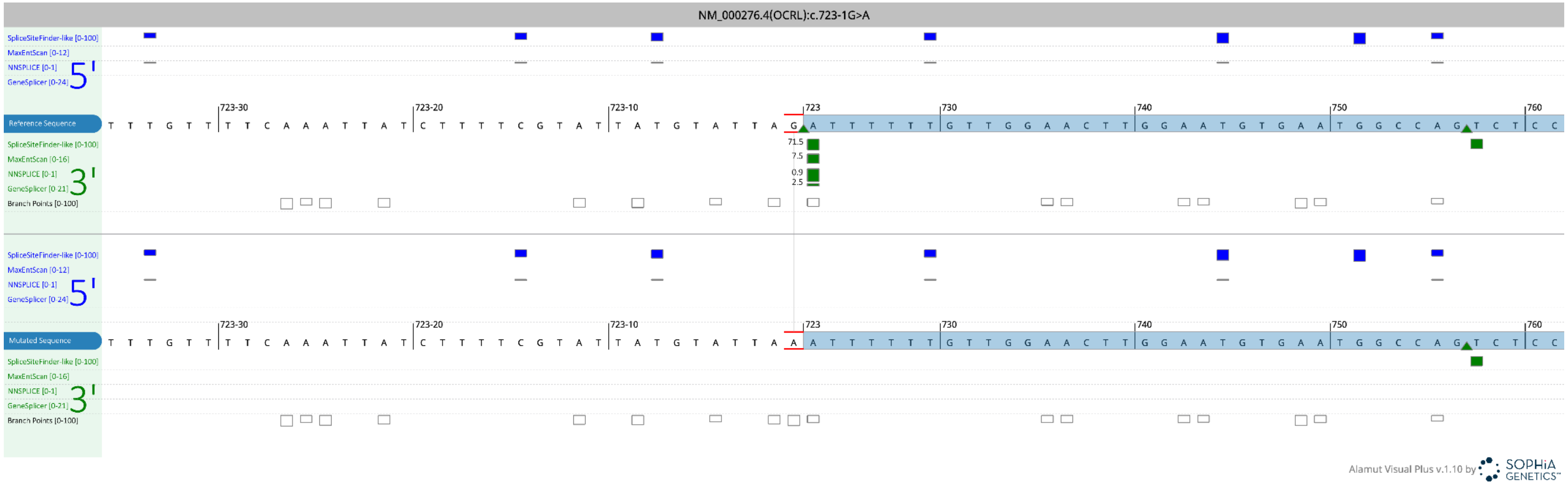

SpliceAI scores: ⓘ

Variant	Gene ☐ = MANE Select transcript ☐ = non-coding transcript	Δ type	Δ score	position	REF score	ALT score
NM_000276.4(OCRL):c.723-1G>A<br>⇒ *X:129560549 G>A*<br>UCSC, gnomAD	OCRL<br>(ENSG00000122126.18 / ENST00000371113.9 / NM_000276.4)<br>protein coding MANE Select transcript<br>(plus strand)<br>OMIM, GTEx, gnomAD, ClinGen, Ensembl, Decipher, GeneCards	Acceptor Loss	0.80	1 bp	0.80	0.00
		Donor Loss	0.58	102 bp	0.81	0.23
		Acceptor Gain	0.10	35 bp	0.00	0.10
		Donor Gain	0.00	408 bp	0.03	0.03

8. c.824 G > C

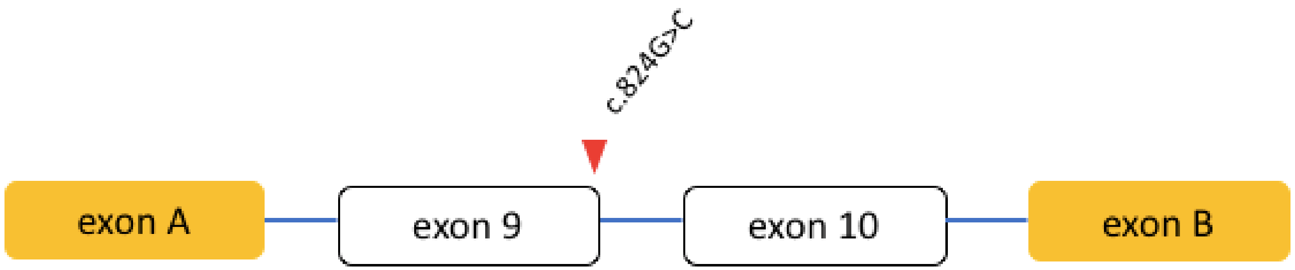

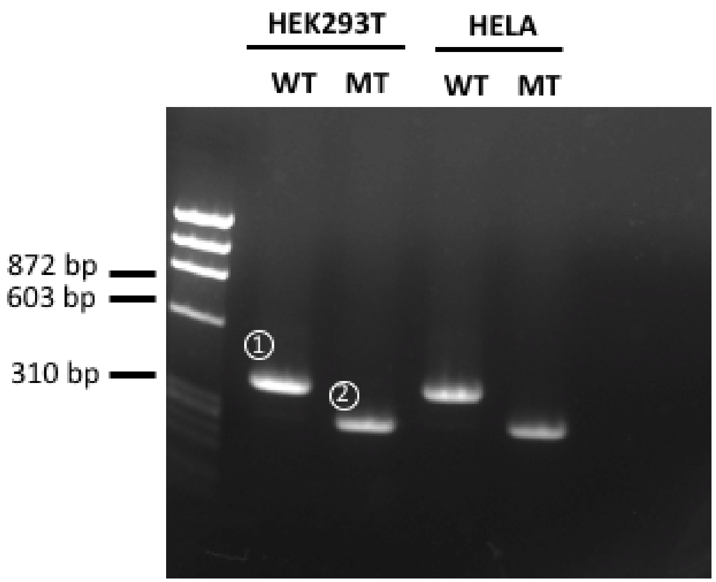

RT-PCR-amplified products of c.824G>C hybrid minigene transcripts

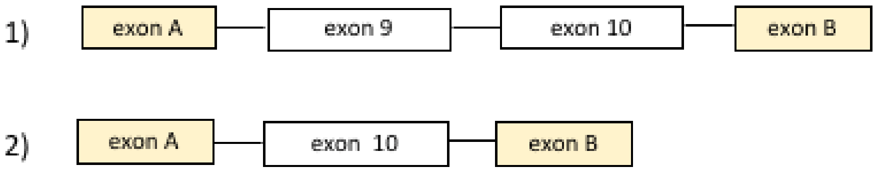

c.824 G > C band No. 1

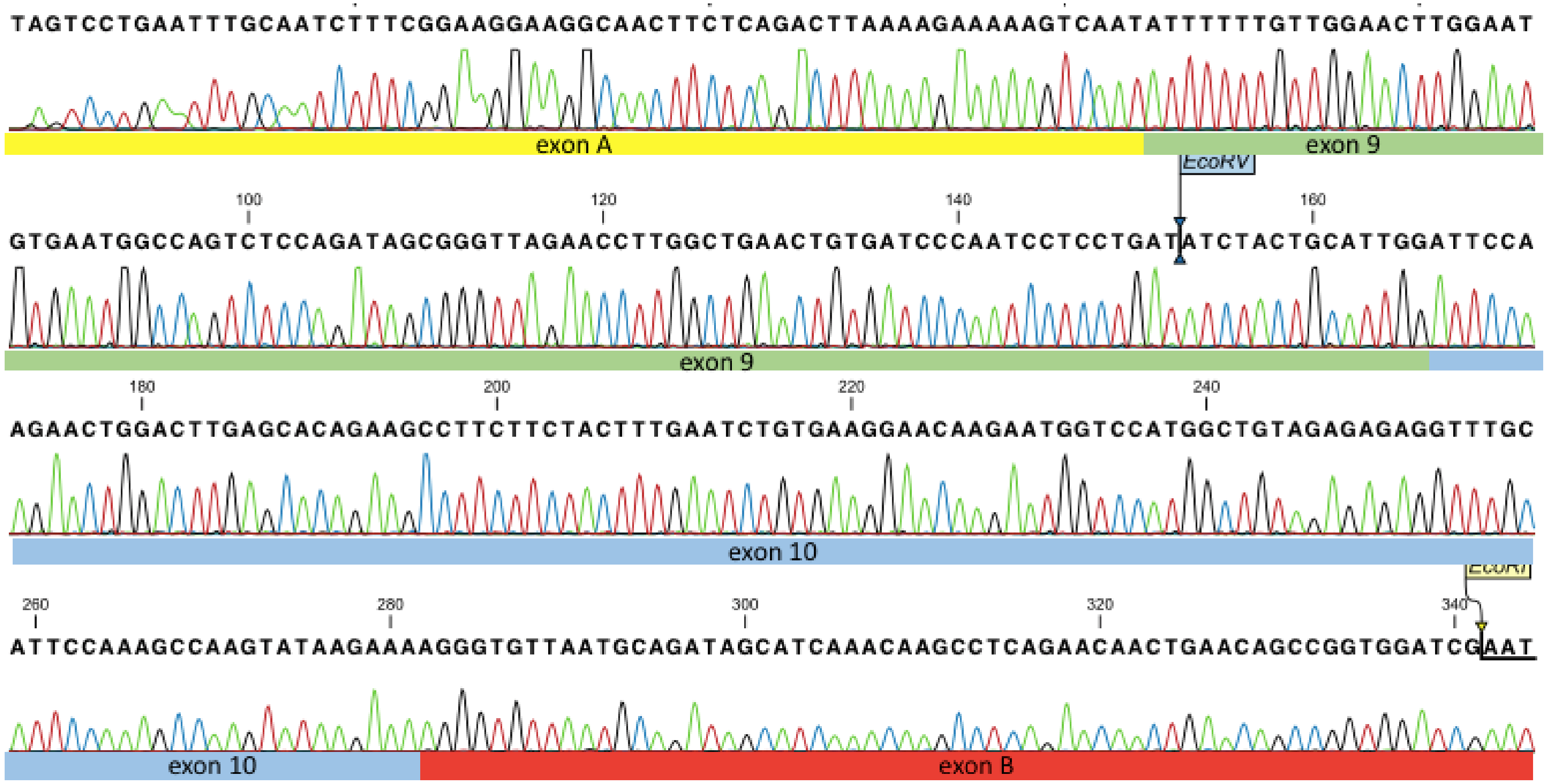

c.824 G > C band No. 2

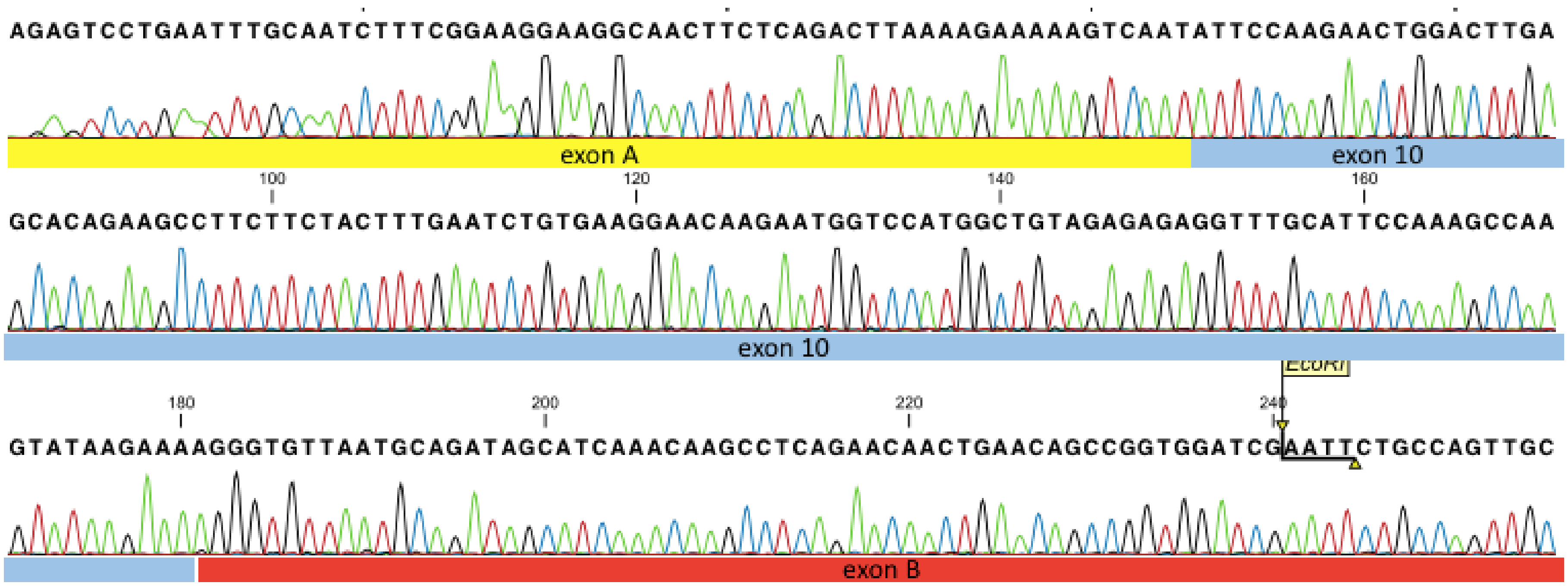

c.824 G > C

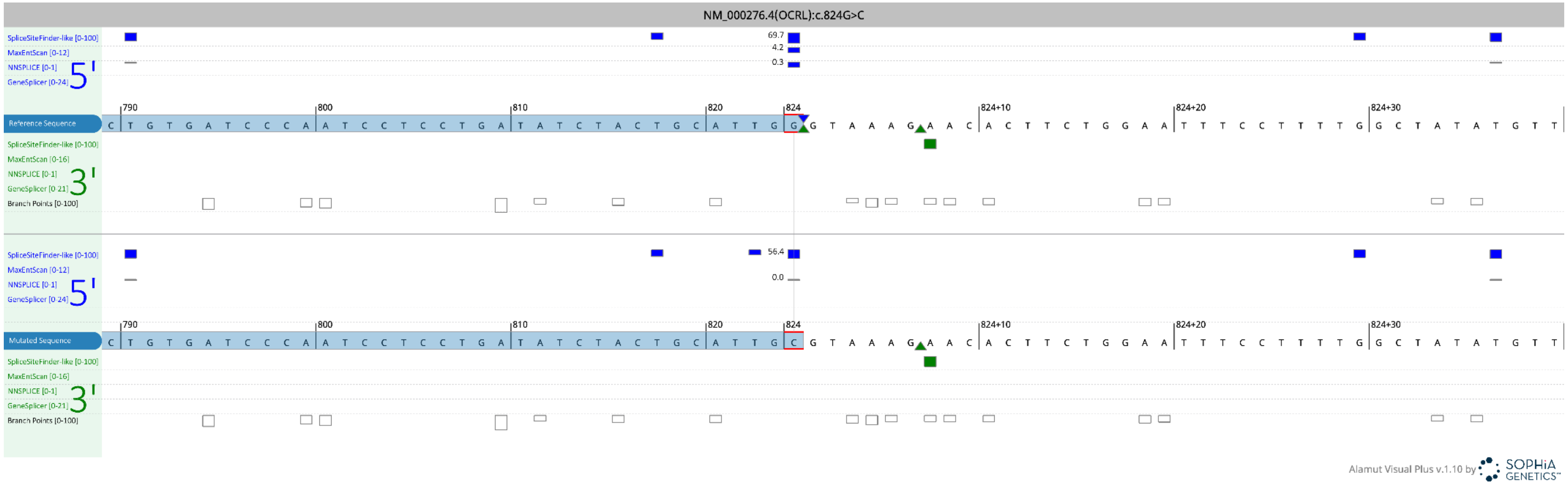

SpliceAI scores: ?

Variant	Gene ☐ = MANE Select transcript ☐ = non-coding transcript	Δ type	Δ score ?	position ?	REF score ?	ALT score ?
NM_000276.4(OCRL):c.824 G>C<br>⇒ *X:129560651 G>C*<br>UCSC, gnomAD	OCRL<br>(ENSG00000122126.18 / ENST00000371113.9 / NM_000276.4)<br>protein coding MANE Select transcript (plus strand)<br>OMIM, GTEx, gnomAD, ClinGen, Ensembl, Decipher, GeneCards	Acceptor Loss	0.74	-101 bp	0.80	0.06
		Donor Loss	0.79	0 bp	0.81	0.02
		Acceptor Gain	0.00		0.00	0.00
		Donor Gain	0.01	306 bp	0.03	0.04

9. c.825-2 A > G

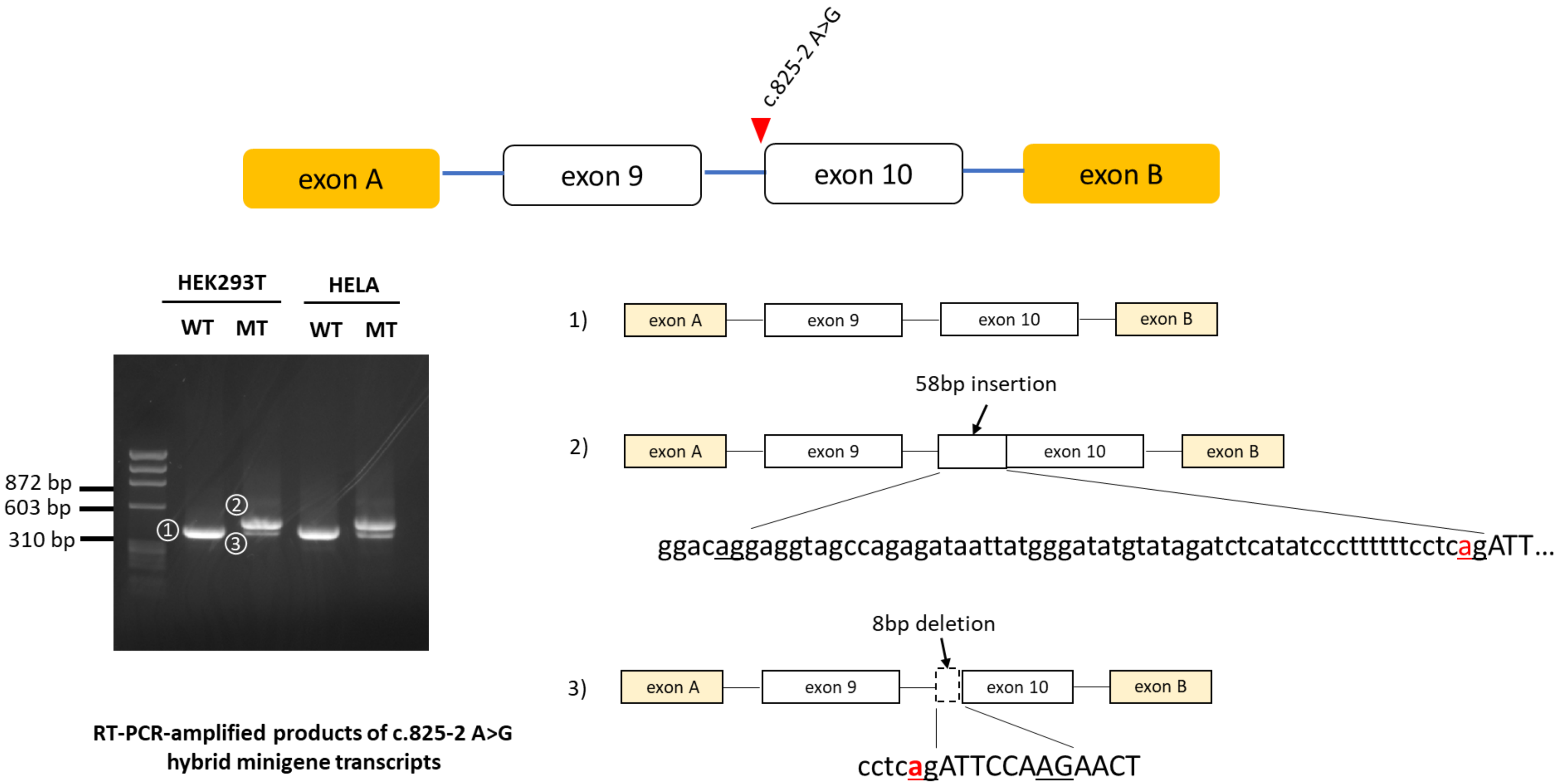

RT-PCR-amplified products of c.825-2 A>G hybrid minigene transcripts

c.825-2 A > G band No. 1

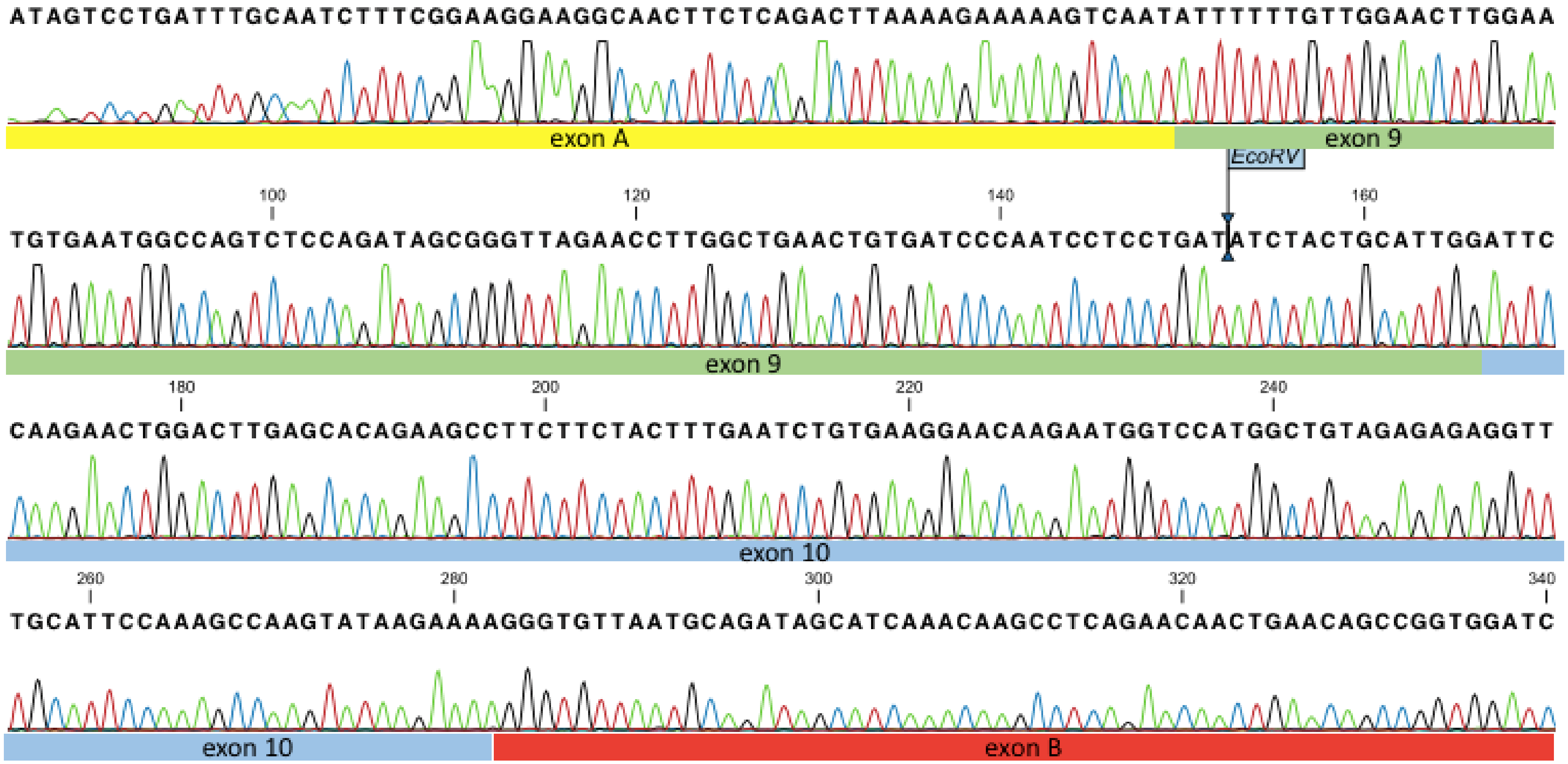

c.825-2 A > G band No. 2

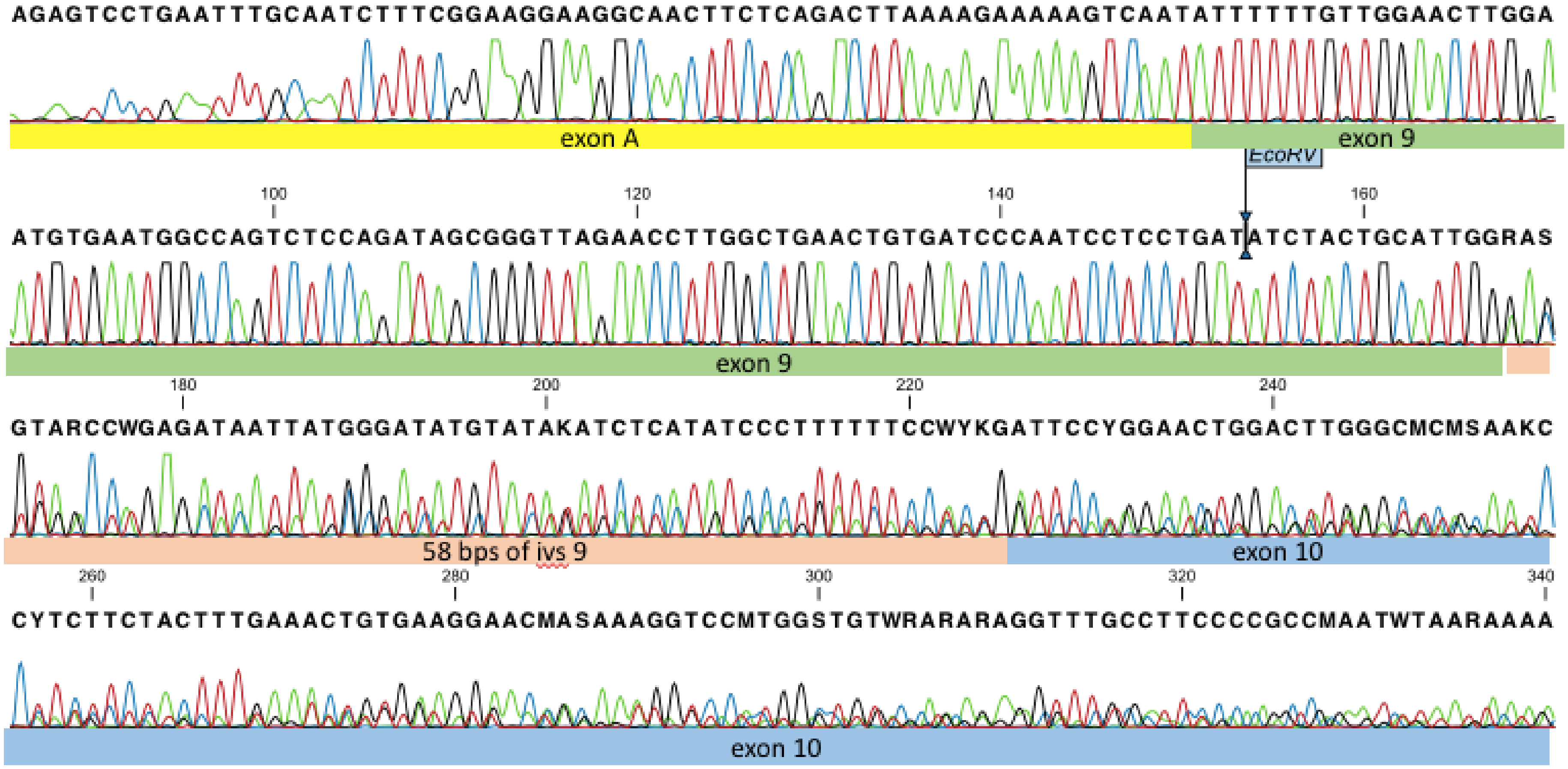

c.825-2 A > G band No. 3

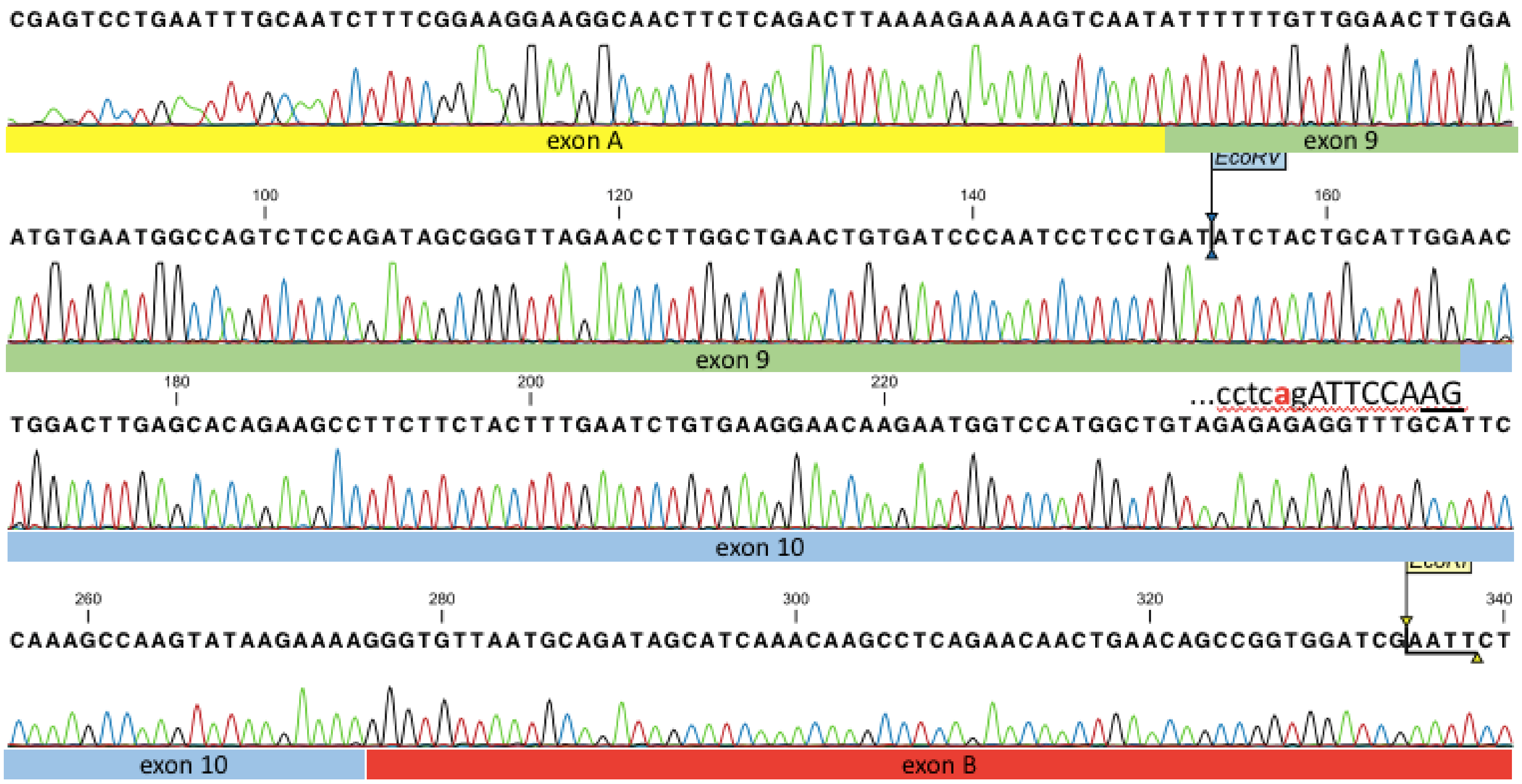

c.825-2 A > G

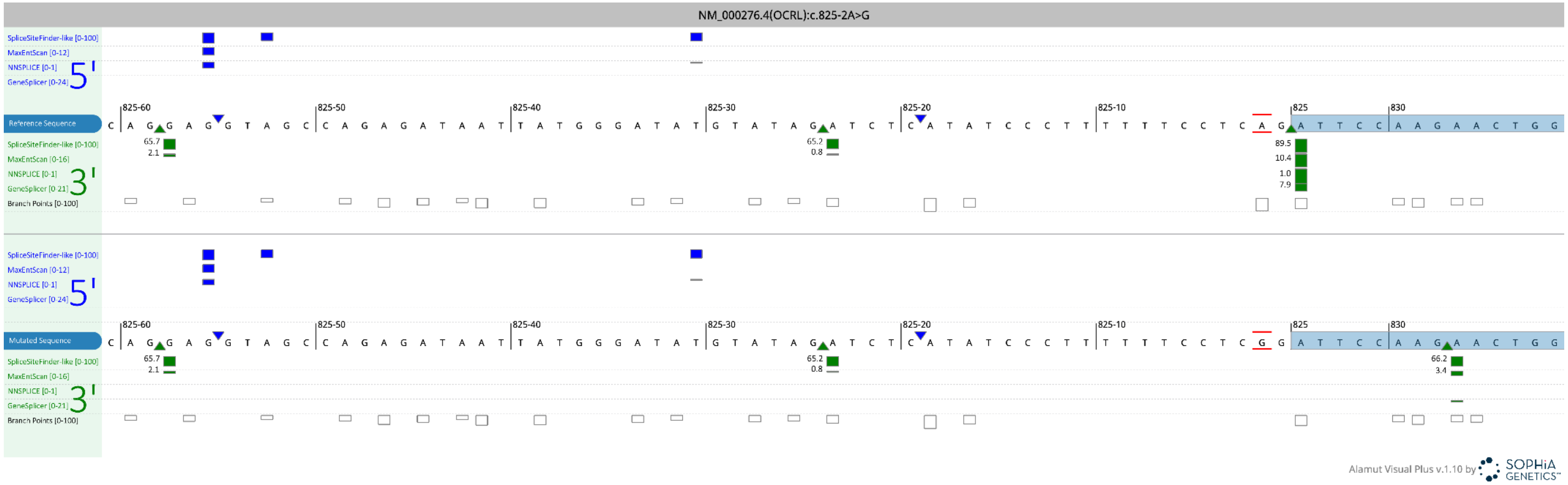

SpliceAI scores:

Variant	Gene ☐ = MANE Select transcript ☐ = non-coding transcript	Δ type	Δ score	position	REF score	ALT score
NM_000276.4(OCRL):c.825-2 A>G<br>⇒ *X:129561177 A>G*<br>UCSC, gnomAD	OCRL<br>(ENSG00000122126.18 / ENST00000371113.9 / NM_000276.4)<br>protein coding MANE Select transcript (plus strand)<br>OMIM, GTEx, gnomAD, ClinGen, Ensembl, Decipher, GeneCards	Acceptor Loss	1.00	2 bp	1.00	0.00
		Donor Loss	0.02	116 bp	1.00	0.98
		Acceptor Gain	0.94	10 bp	0.00	0.94
		Donor Gain	0.09	87 bp	0.22	0.31

10. c.939+3 A > C

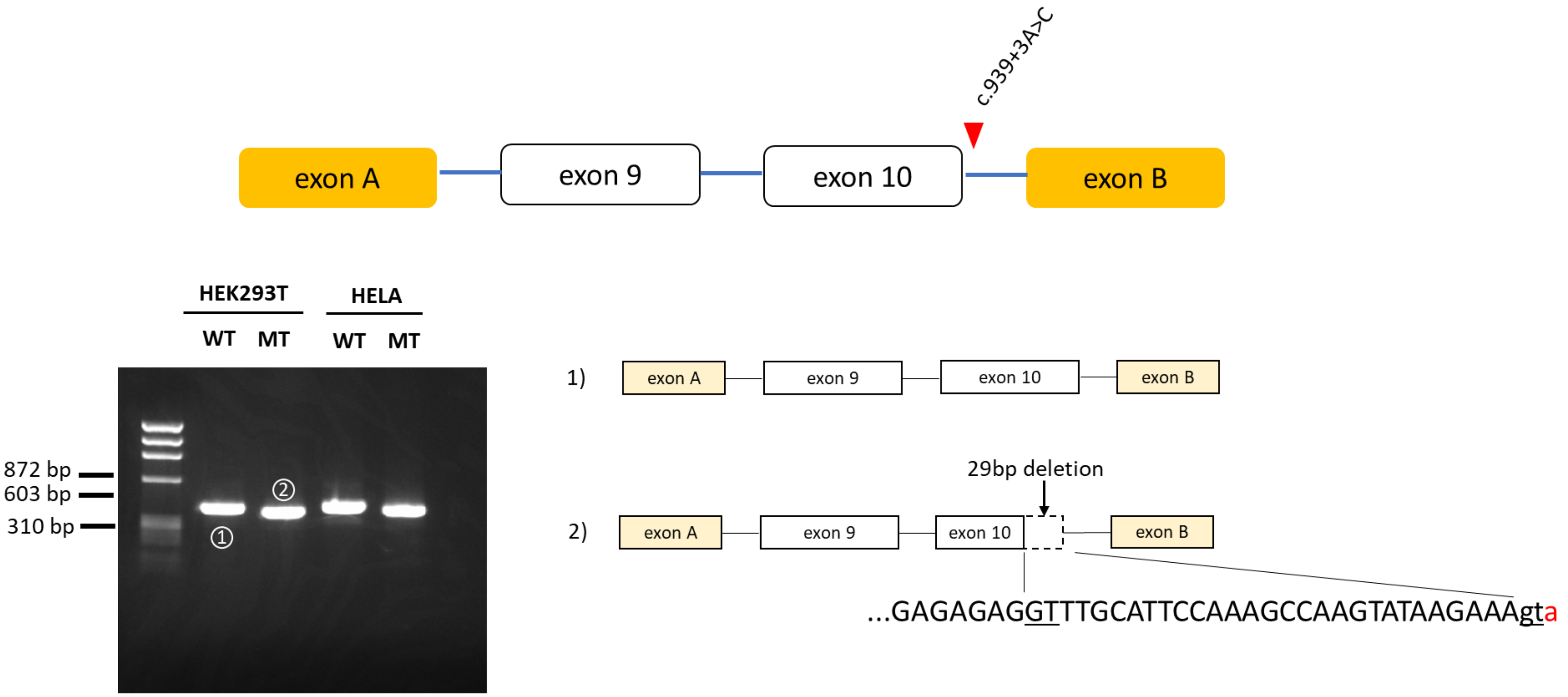

RT-PCR-amplified products of c.939+3A>C hybrid minigene transcripts

c.939+3 A > C band No. 1

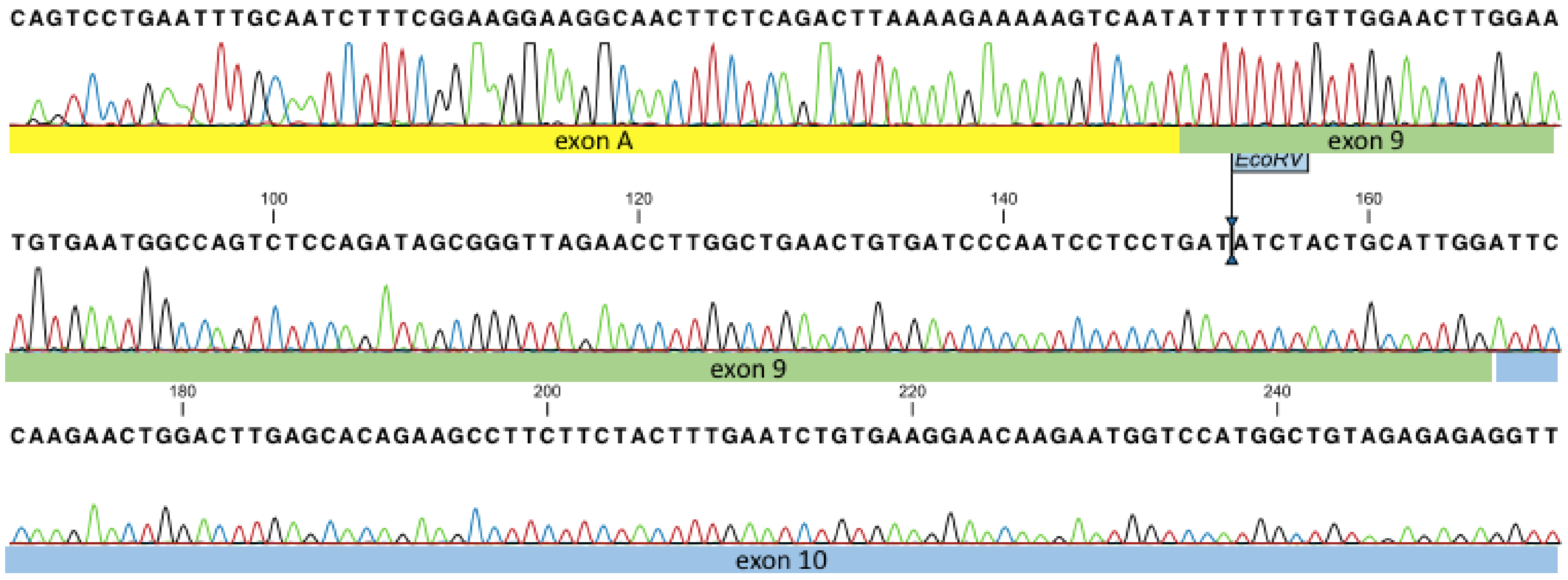

c.939+3 A > C band No. 2

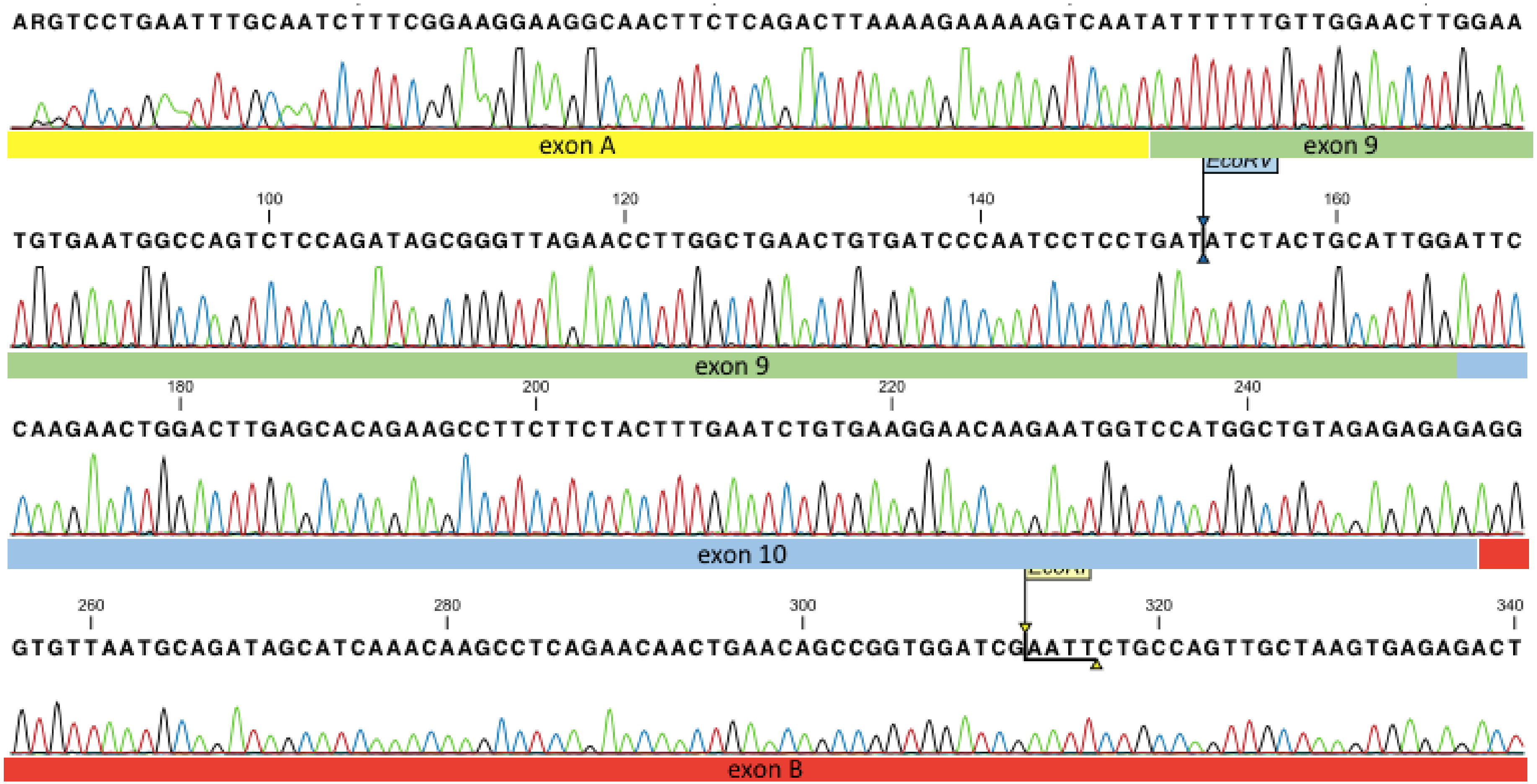

c.939+3 A > C

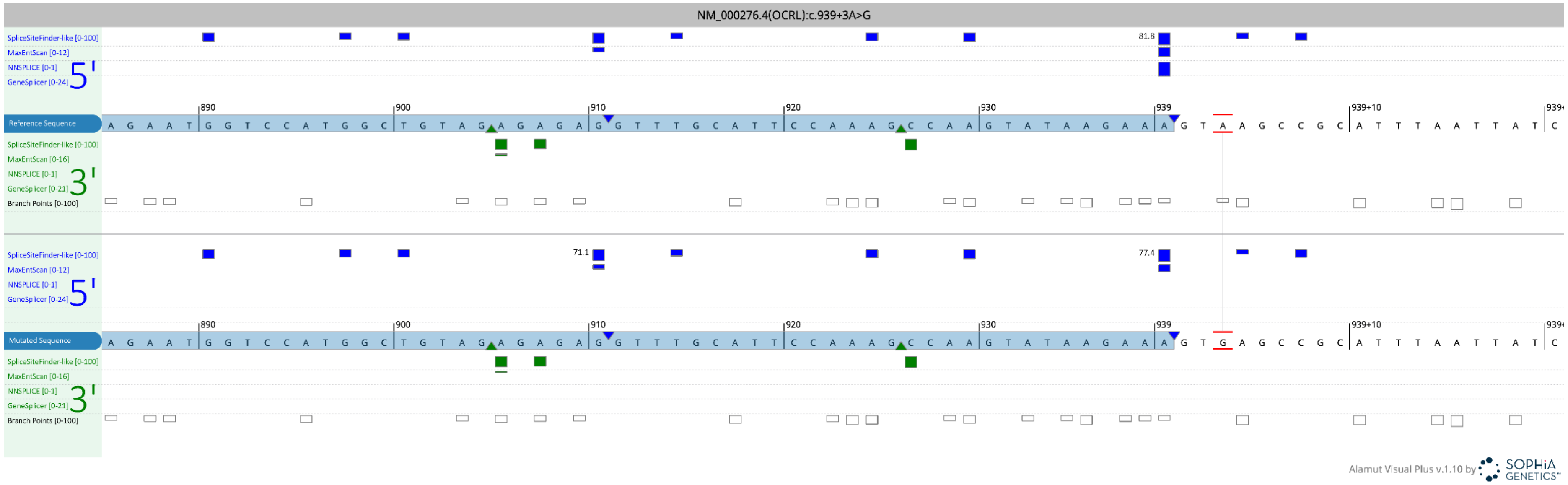

SpliceAI scores:

Variant	Gene (= MANE Select transcript; = non-coding transcript)	Δ type	Δ score	position	REF score	ALT score
NM_000276.4(OCRL):c.939+3A>C<br>⇒ *X:129561296 A>C*<br>UCSC, gnomAD	OCRL<br>(ENSG00000122126.18 / ENST00000371113.9 / NM_000276.4)<br>protein coding MANE Select transcript (plus strand)<br>OMIM, GTEx, gnomAD, ClinGen, Ensembl, Decipher, GeneCards	Acceptor Loss	0.00	-117 bp	1.00	0.99
		Donor Loss	0.62	-3 bp	1.00	0.38
		Acceptor Gain	0.00	-141 bp	0.08	0.08
		Donor Gain	0.78	-32 bp	0.22	1.00

11. c.940-11 G > A

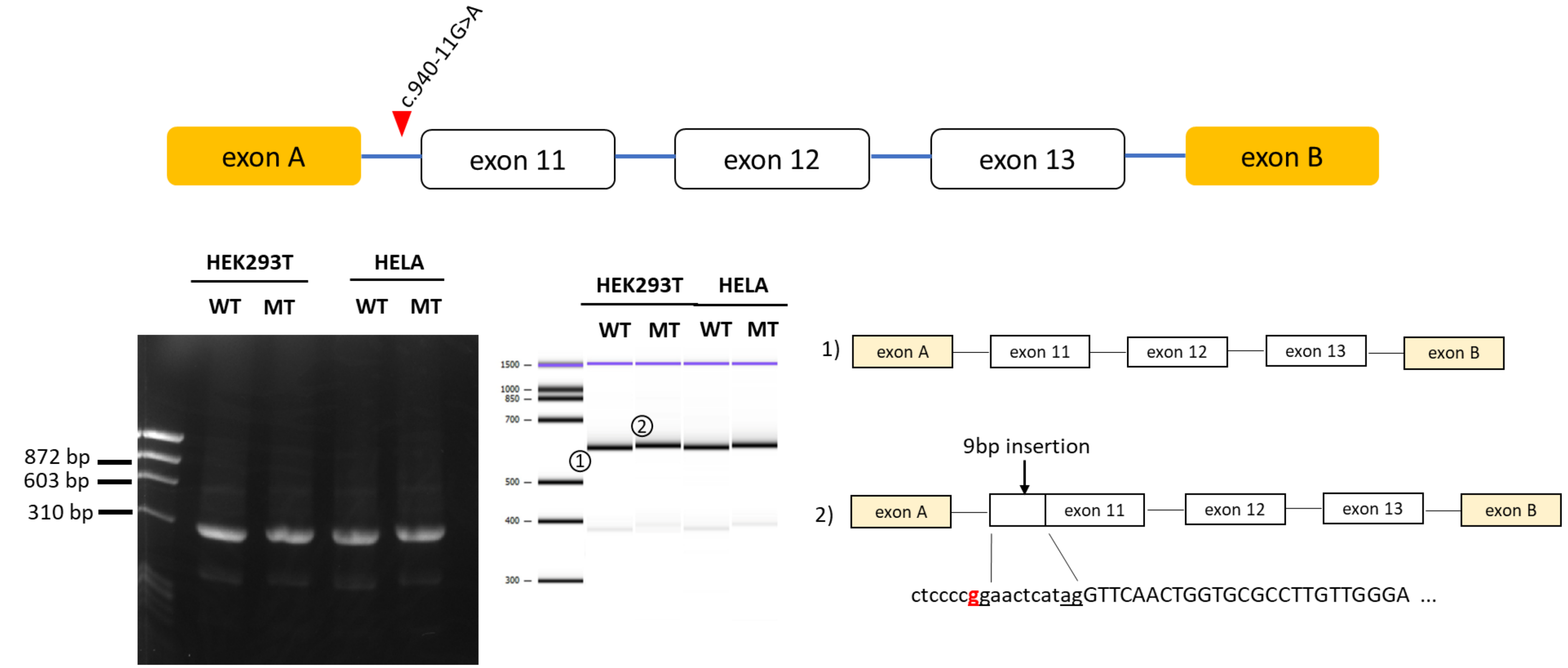

RT-PCR-amplified products of c.940-11 G>A
hybrid minigene transcripts (left :electrophoresis; right: DNA1000 assay)

c.940-11 G > A band No. 1

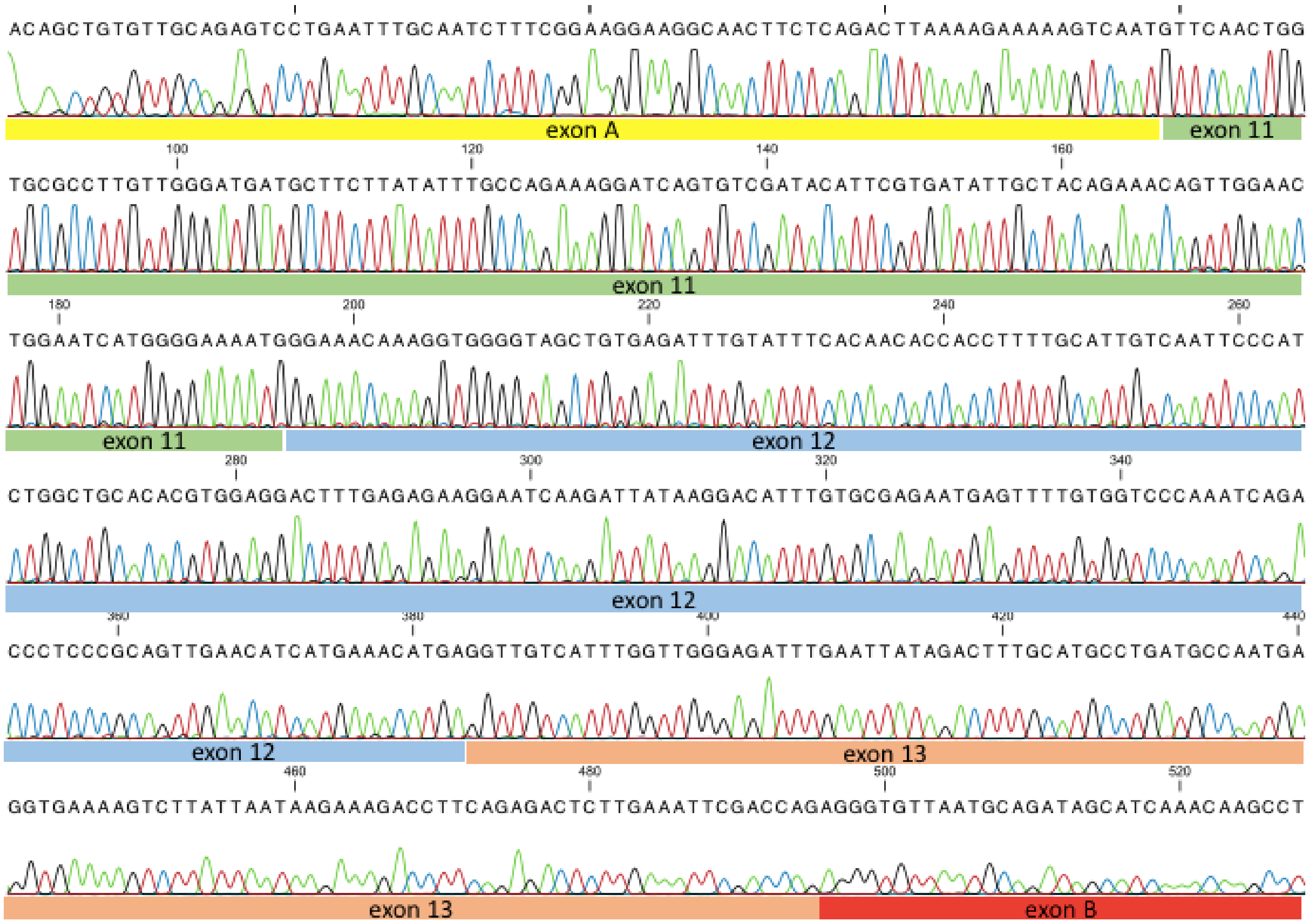

c.940-11 G > A band No. 2

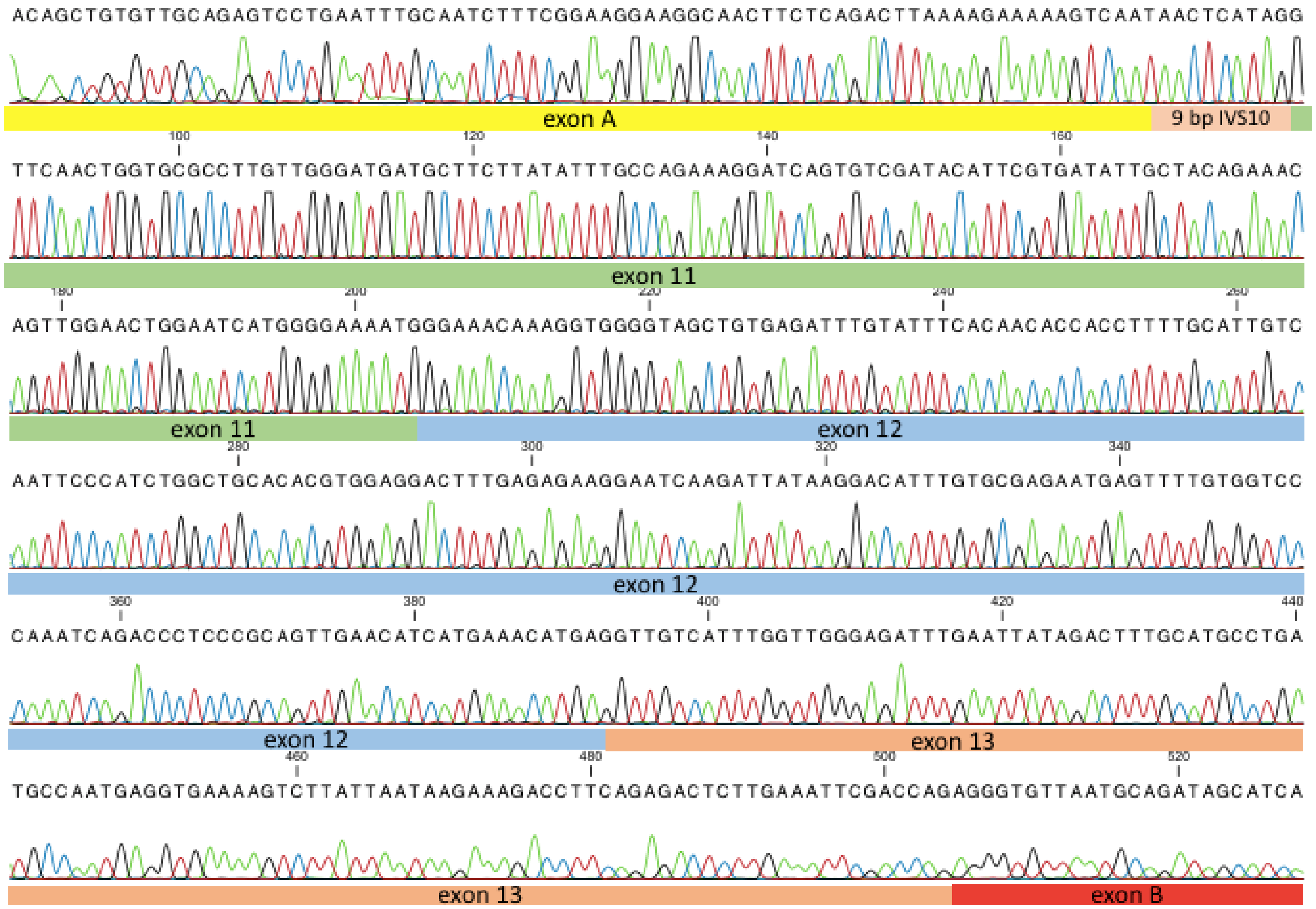

c.940-11 G > A

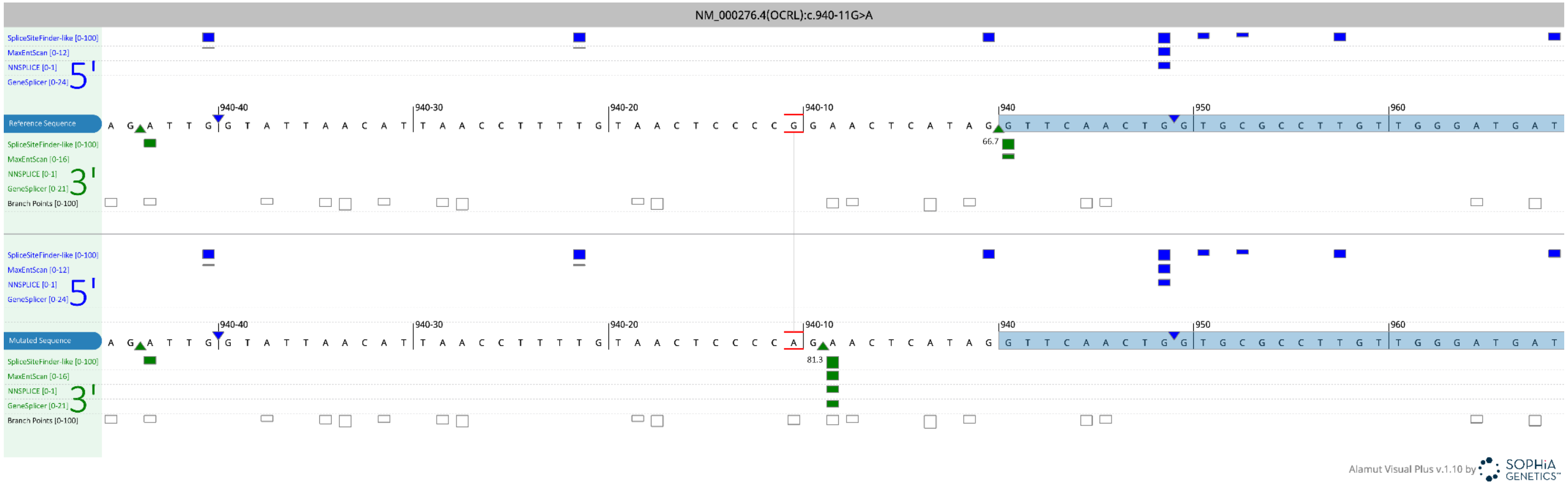

SpliceAI scores:

Variant	Gene (= MANE Select transcript; = non-coding transcript)	Δ type	Δ score	position	REF score	ALT score
NM_000276.4(OCRL):c.940-11 G>A ⇒ X:129562373 G>A UCSC, gnomAD	OCRL (ENSG00000122126.18 / ENST00000371113.9 / NM_000276.4) protein coding MANE Select transcript (plus strand) OMIM, GTEx, gnomAD, ClinGen, Ensembl, Decipher, GeneCards	Acceptor Loss	0.74	11 bp	1.00	0.25
		Donor Loss	0.00		0.00	0.00
		Acceptor Gain	1.00	2 bp	0.00	1.00
		Donor Gain	0.00	413 bp	0.99	0.99

12. c.940-1 G > A

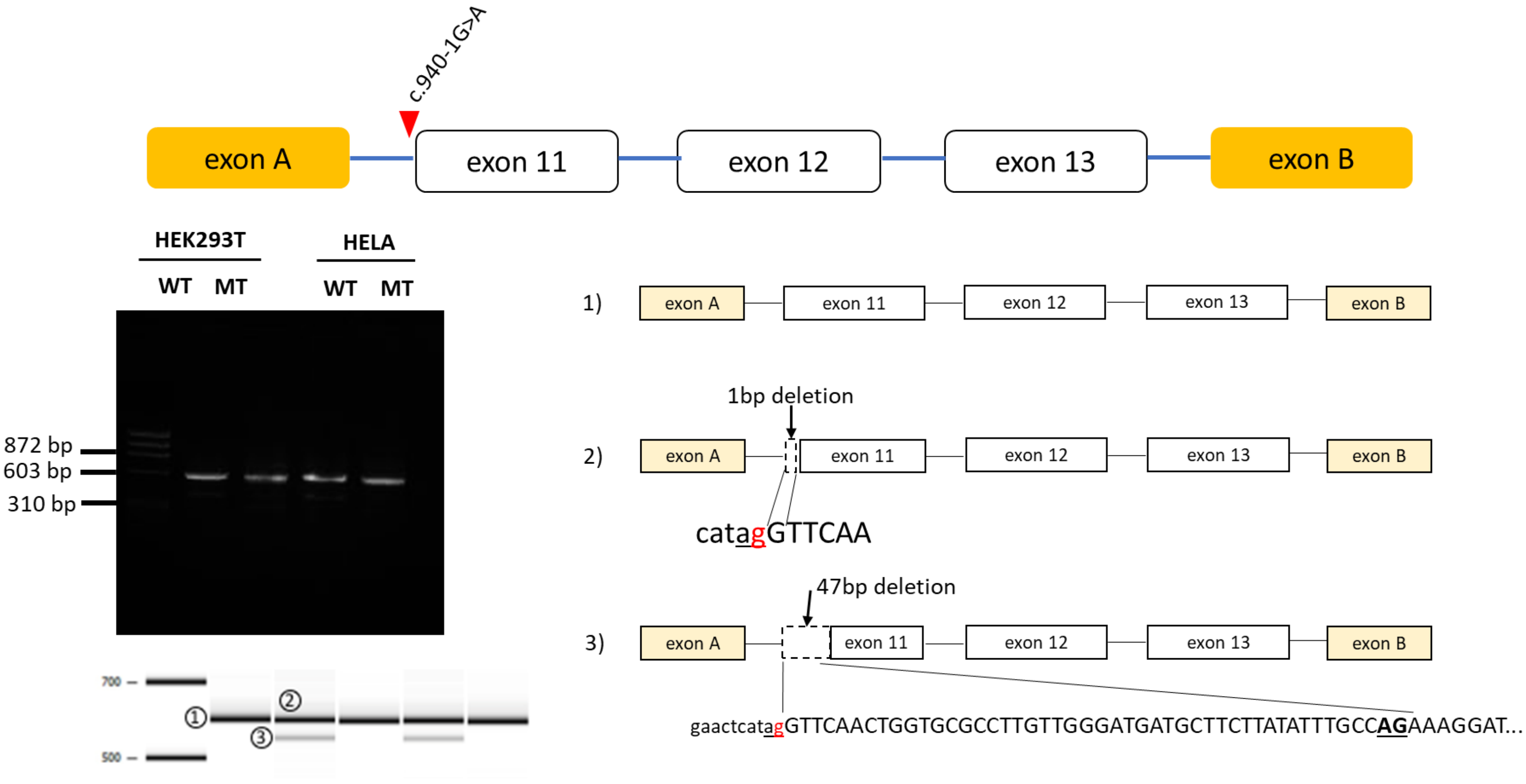

RT-PCR-amplified products of c.940-1 G>A hybrid minigene transcripts (upper : electrophoresis; lower :DNA 1000 Assay)

c.940-1 G > A band No. 1

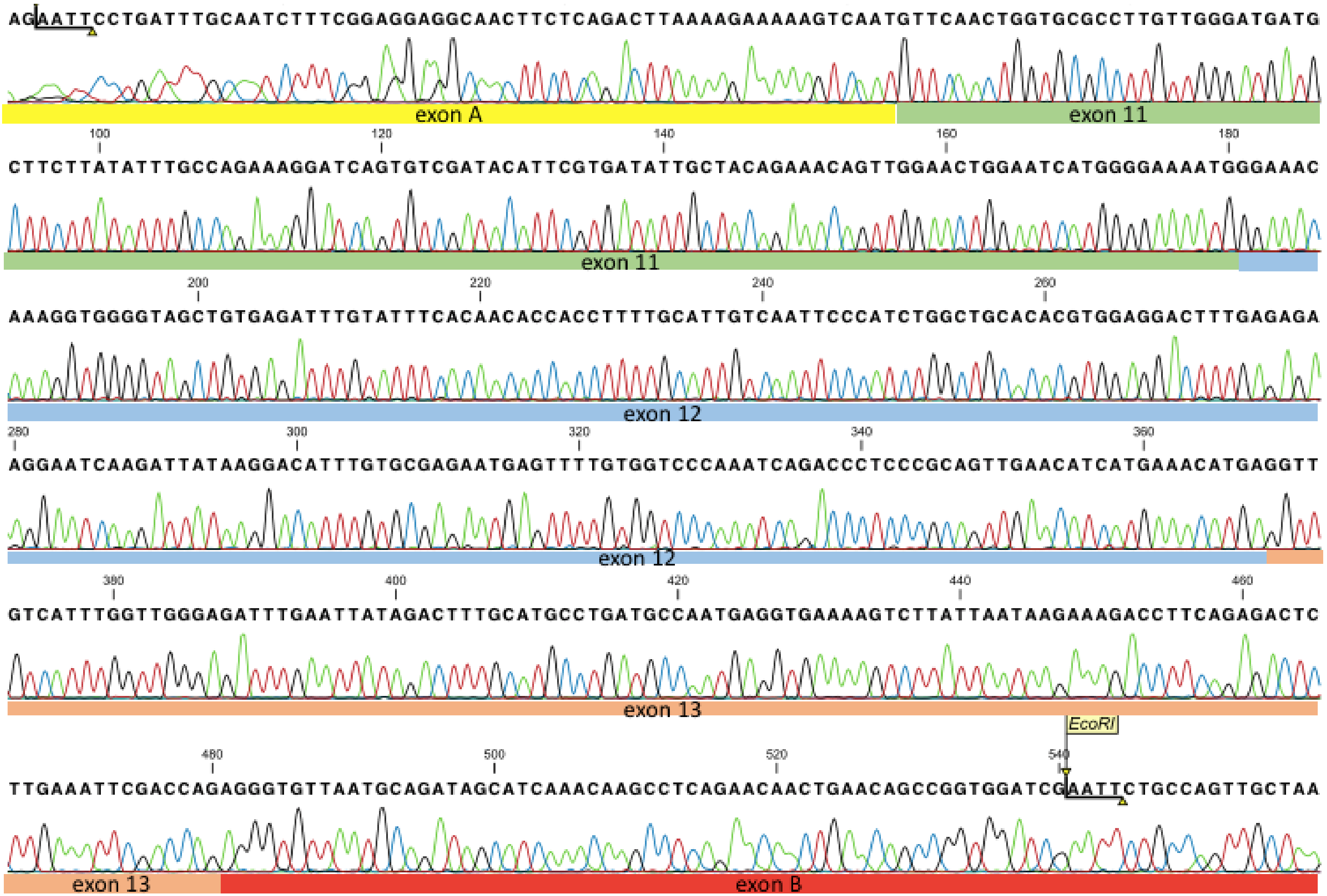

c.940-1 G > A band No. 2

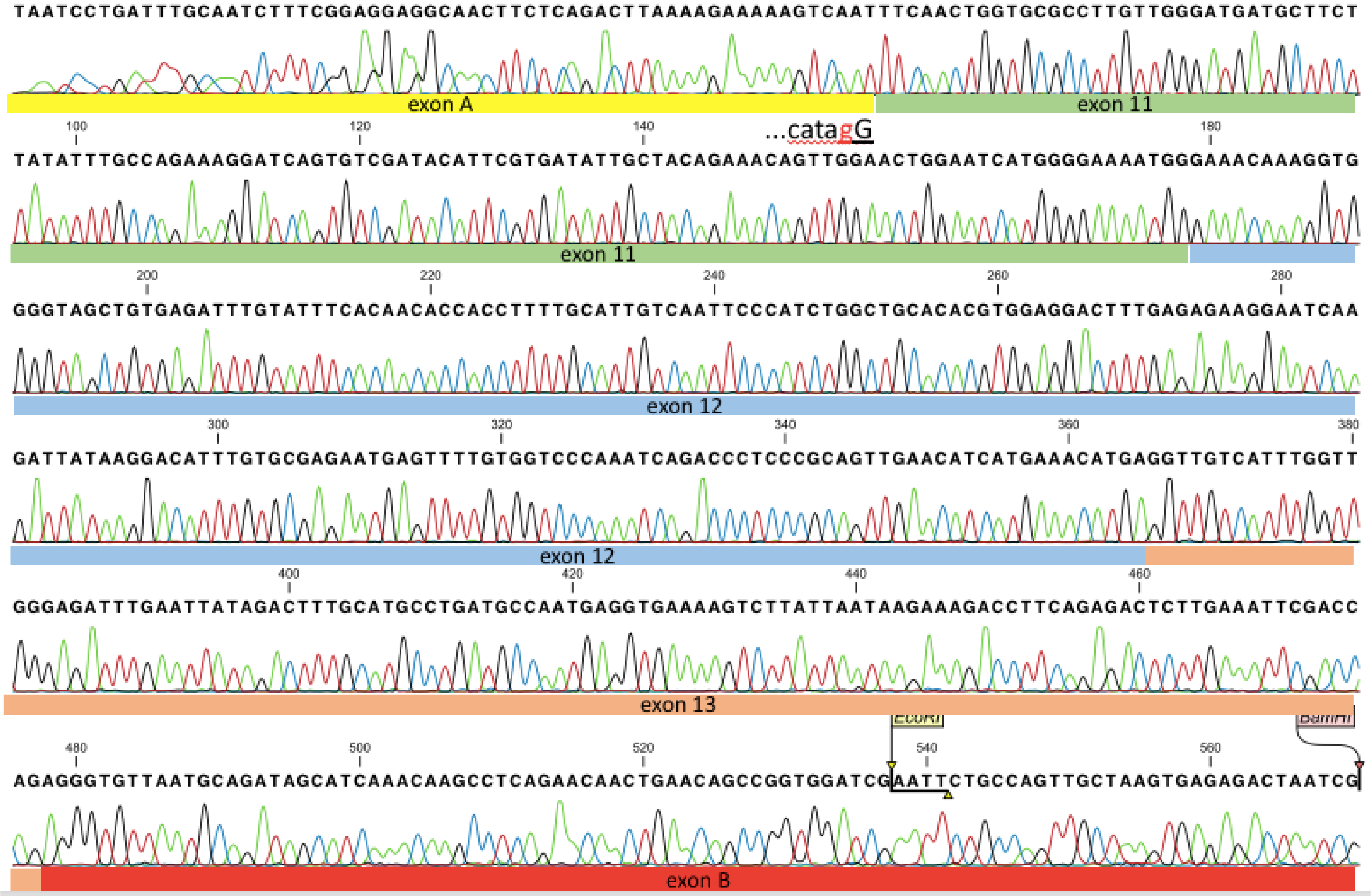

c.940-1 G > A band No. 3

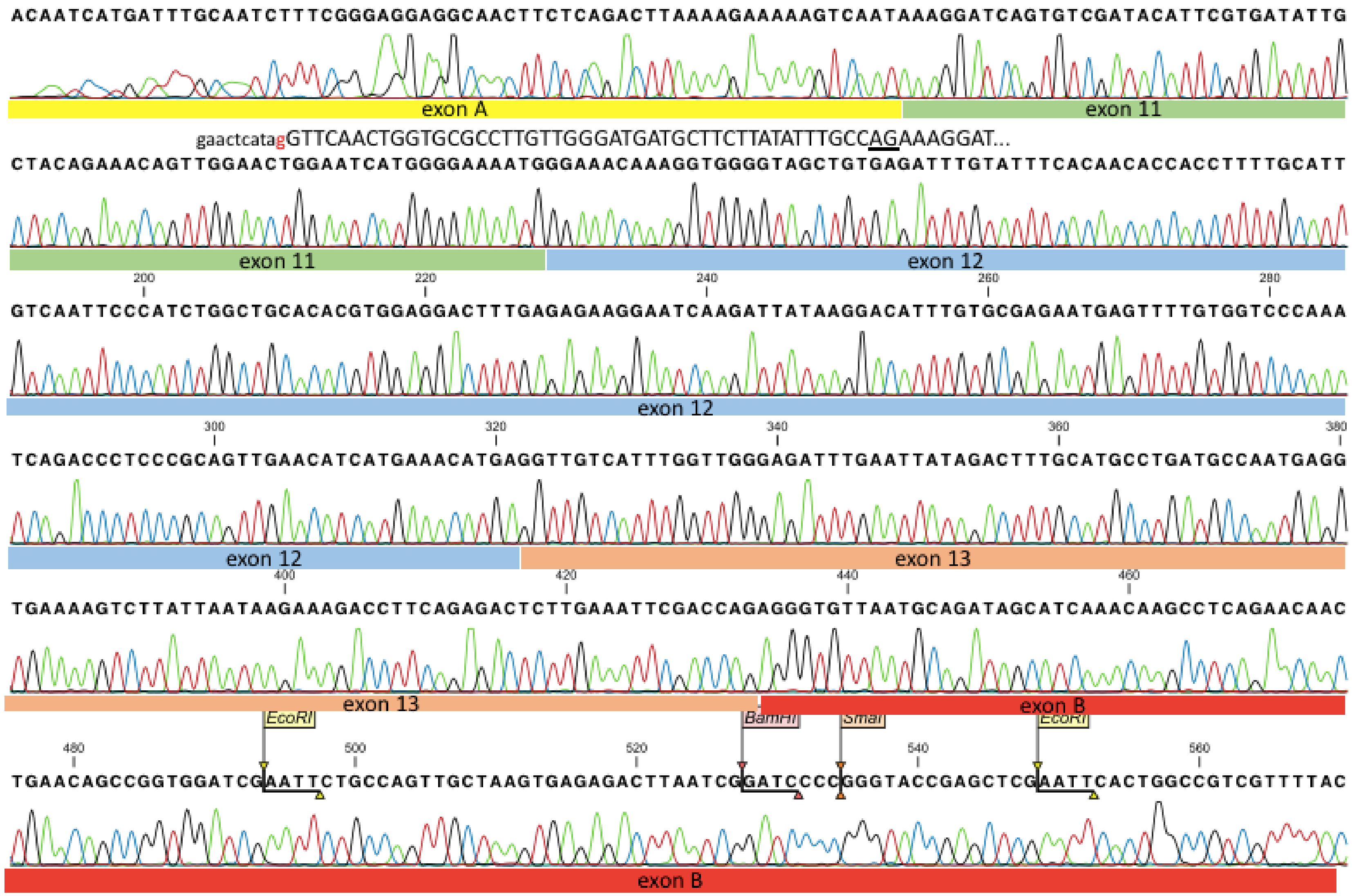

c.940-1 G > A

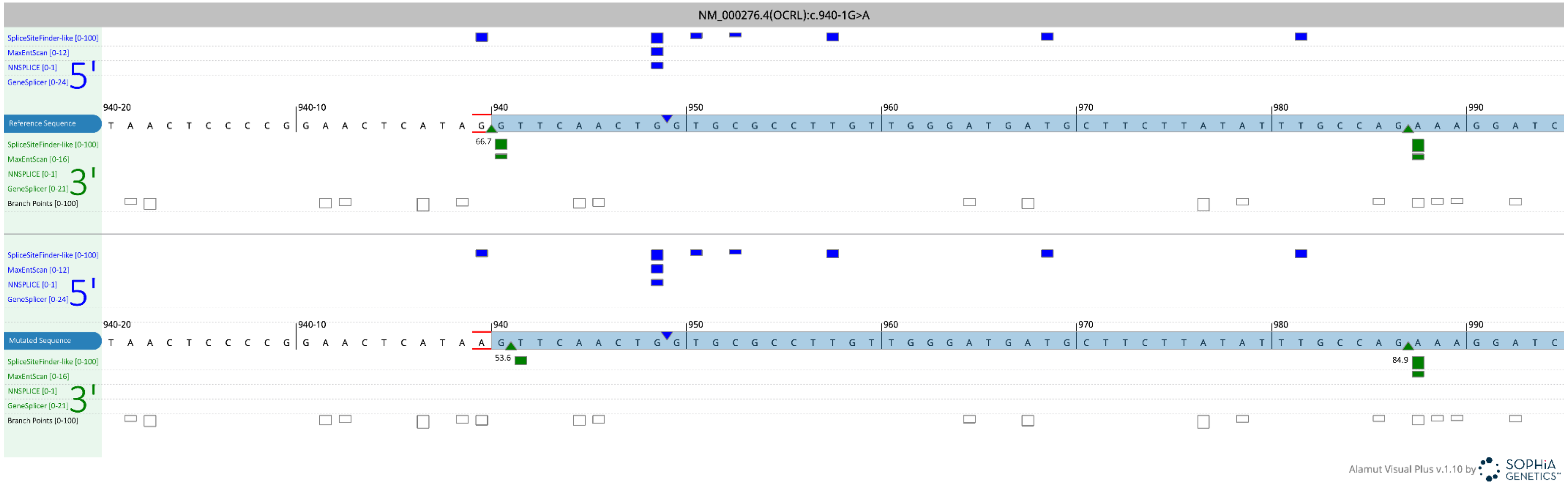

SpliceAI scores:

Variant	Gene ☐ = MANE Select transcript ☐ = non-coding transcript	Δ type	Δ score	position	REF score	ALT score
NM_000276.4(OCRL):c.940-1G>A<br>⇒ *X:129562383 G>A*<br>UCSC, gnomAD	OCRL<br>(ENSG00000122126.18 / ENST00000371113.9 / NM_000276.4)<br>protein coding MANE Select transcript<br>(plus strand)<br>OMIM, GTEx, gnomAD, ClinGen, Ensembl, Decipher, GeneCards	Acceptor Loss	0.99	1 bp	1.00	0.00
		Donor Loss	0.00	117 bp	1.00	1.00
		Acceptor Gain	0.82	2 bp	0.00	0.82
		Donor Gain	0.00		0.00	0.00

13. c.1244+1 G > C

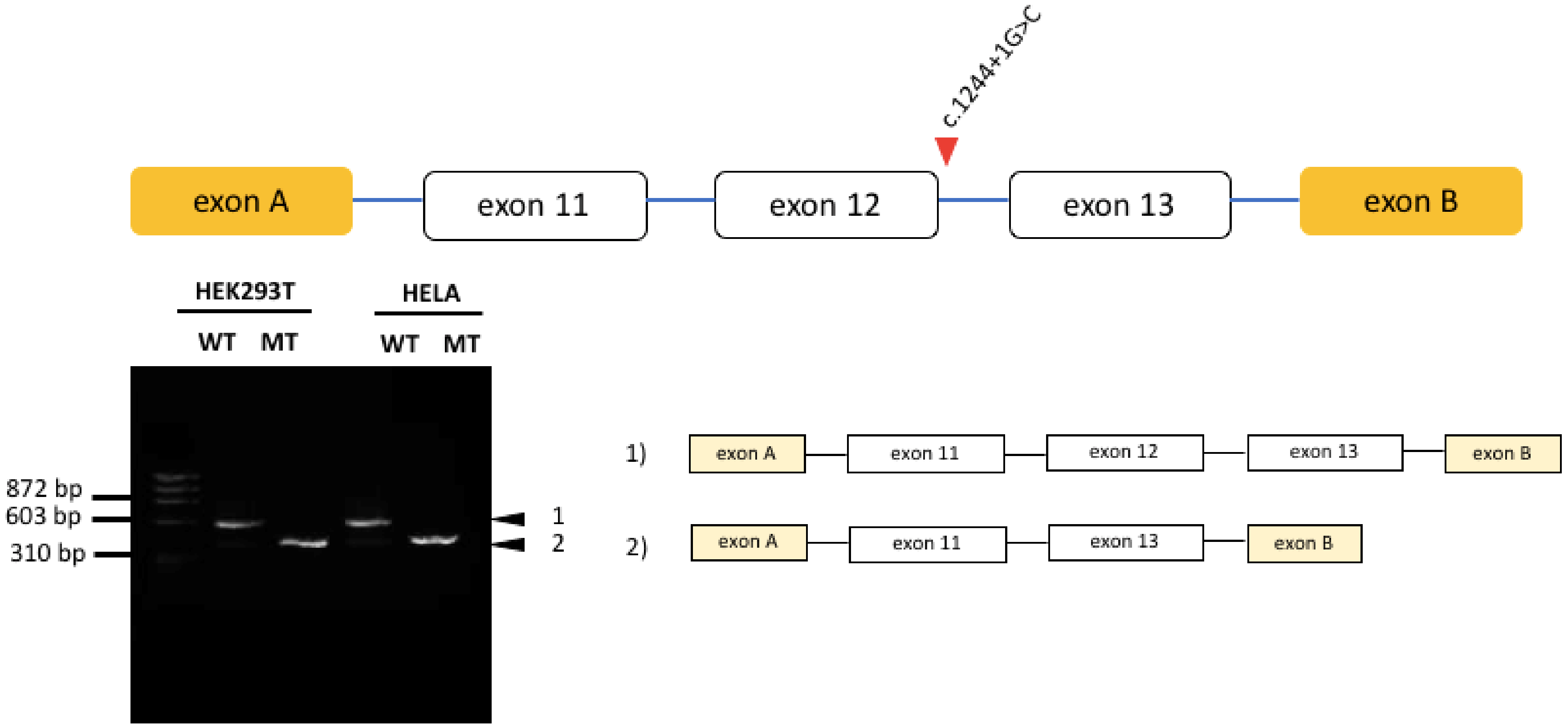

RT-PCR-amplified products of c.1244+1 G>C hybrid minigene transcripts

c.1244+1 G > C band No. 1

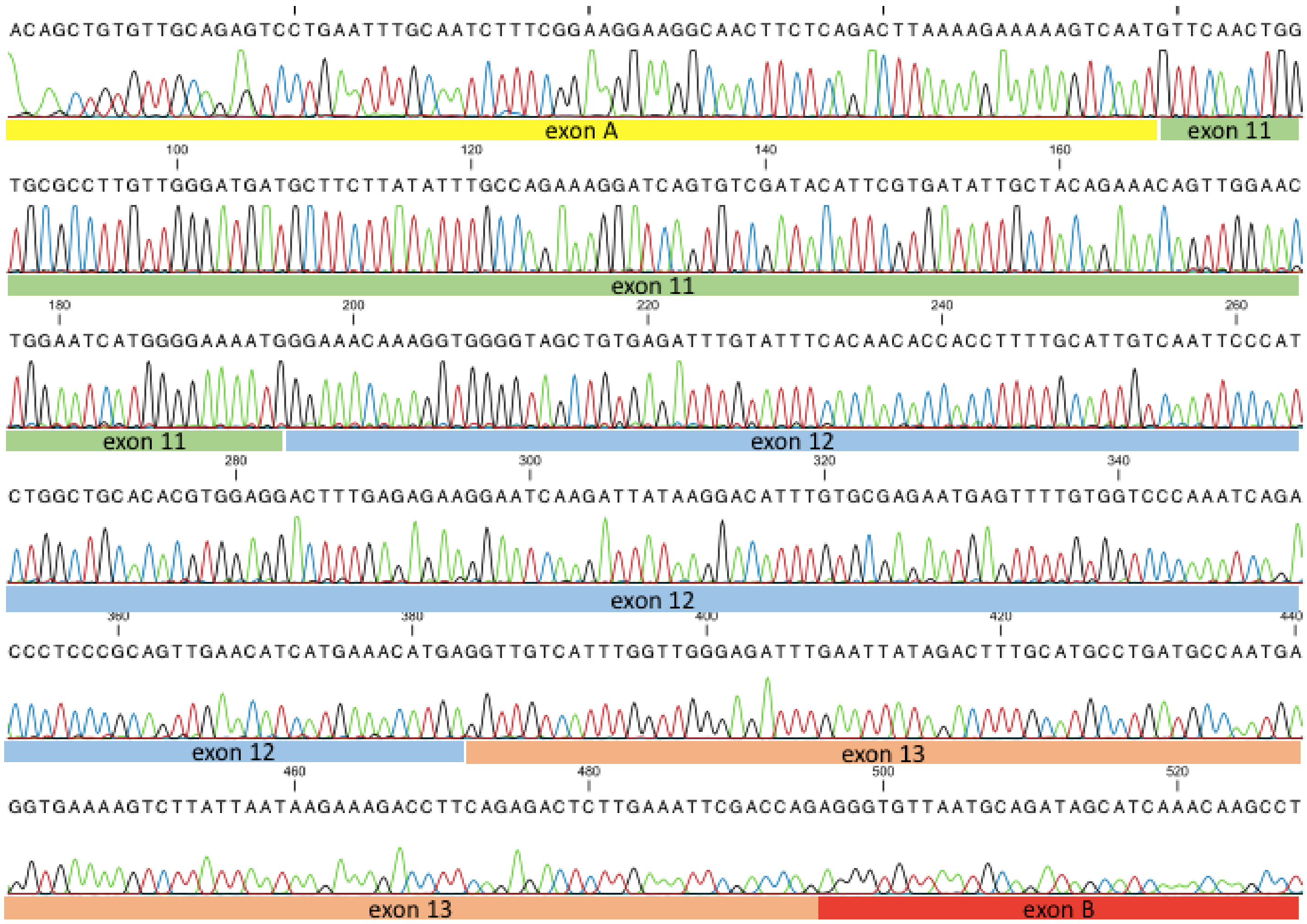

c.1244+1 G > C band No. 2

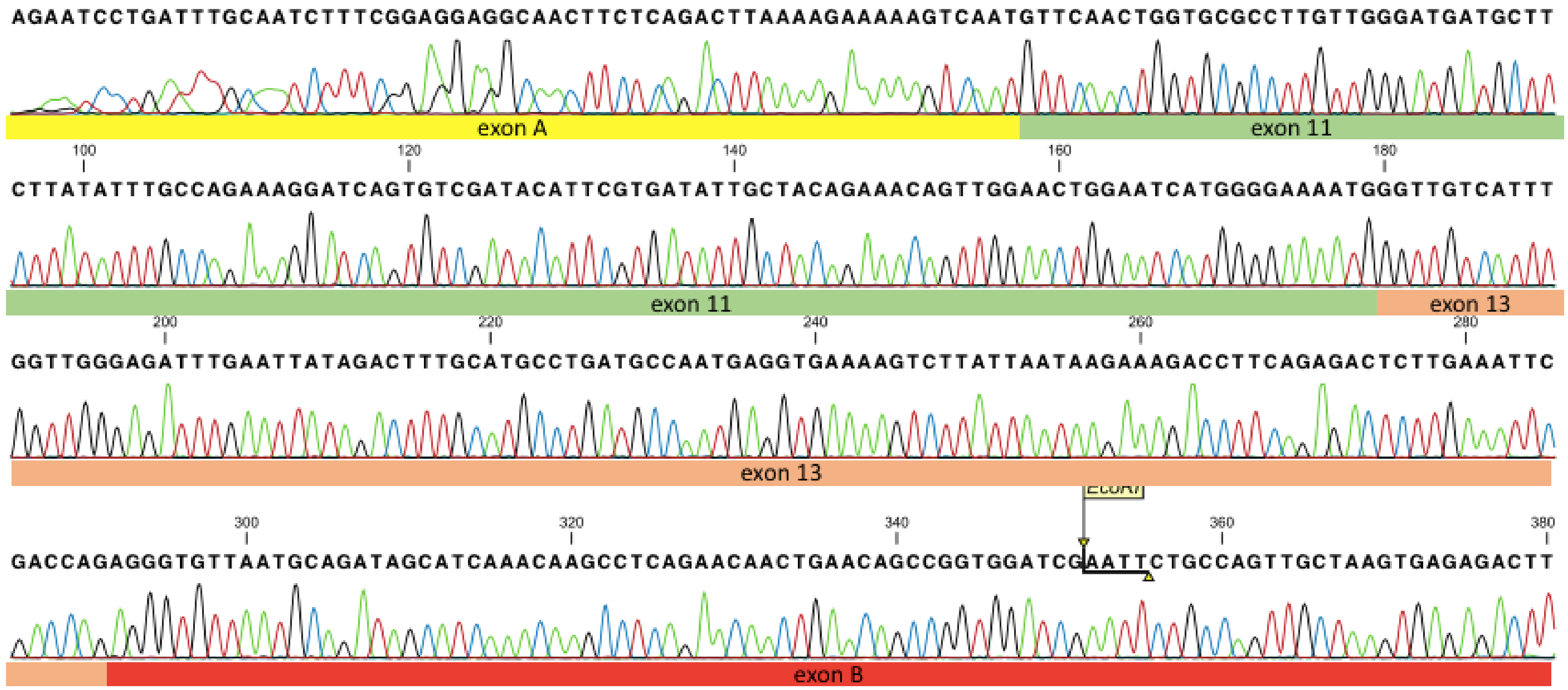

c.1244+1 G > C

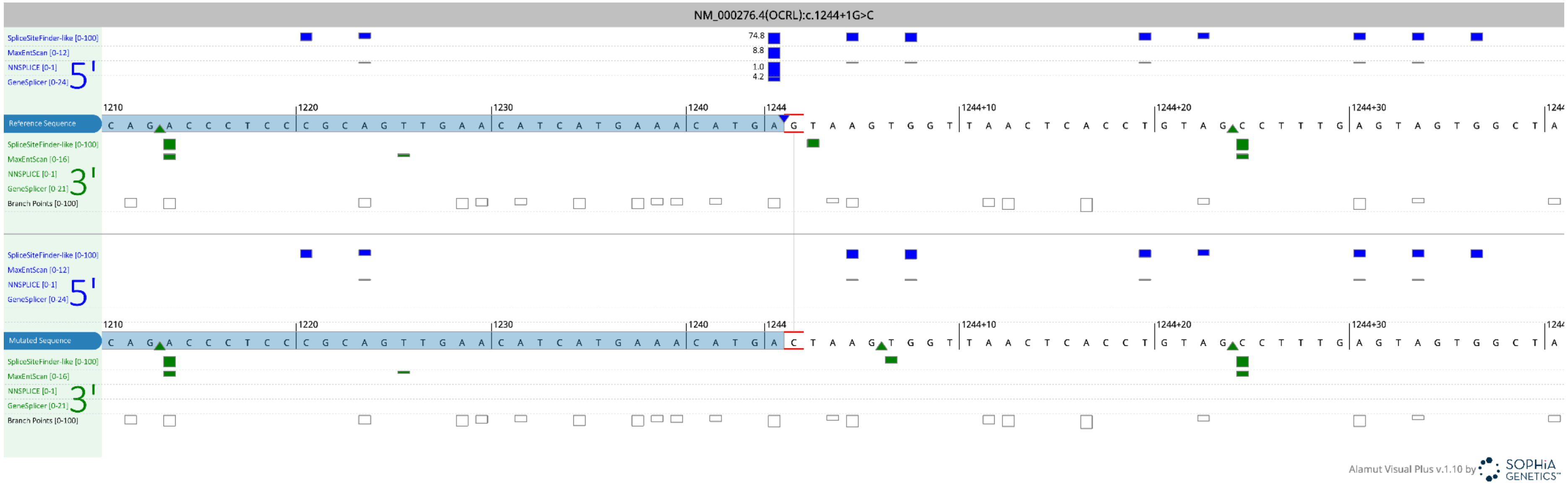

SpliceAI scores: ⓘ

Variant	Gene □ = MANE Select transcript □ = non-coding transcript	Δ type	Δ score	position	REF score	ALT score
NM_000276.4(OCRL):c.1244+1G>C<br>⇒ *X:129562787 G>C*<br>UCSC, gnomAD	OCRL<br>(ENSG00000122126.18 / ENST00000371113.9 / NM_000276.4)<br>protein coding MANE Select transcript (plus strand)<br>OMIM, GTEx, gnomAD, ClinGen, Ensembl, Decipher, GeneCards	Acceptor Loss	0.58	-188 bp	0.99	0.41
		Donor Loss	0.99	-1 bp	0.99	0.00
		Acceptor Gain	0.00	-352 bp	0.01	0.01
		Donor Gain	0.04	3 bp	0.00	0.05

14. c.1466 G > A

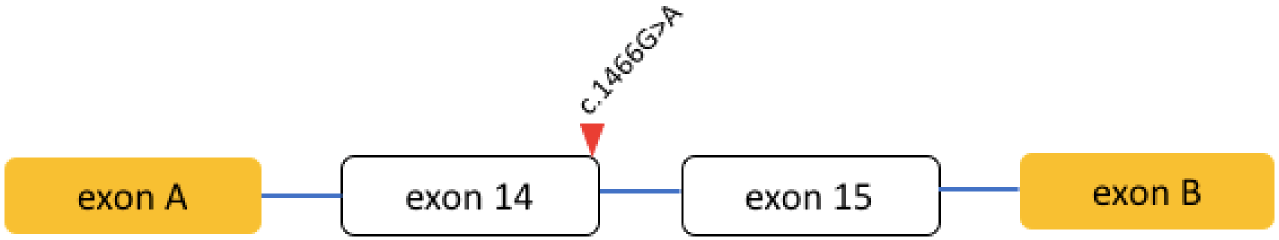

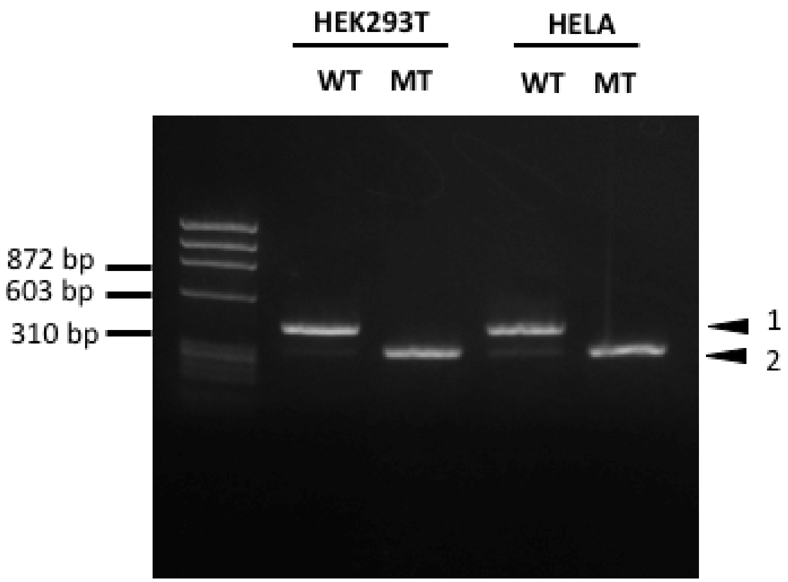

RT-PCR-amplified products of c.1466G>A hybrid minigene transcripts

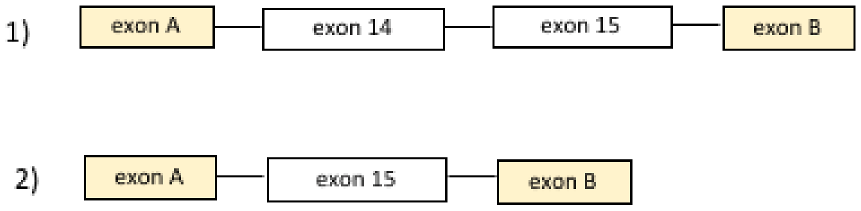

c.1466 G > A band No. 1

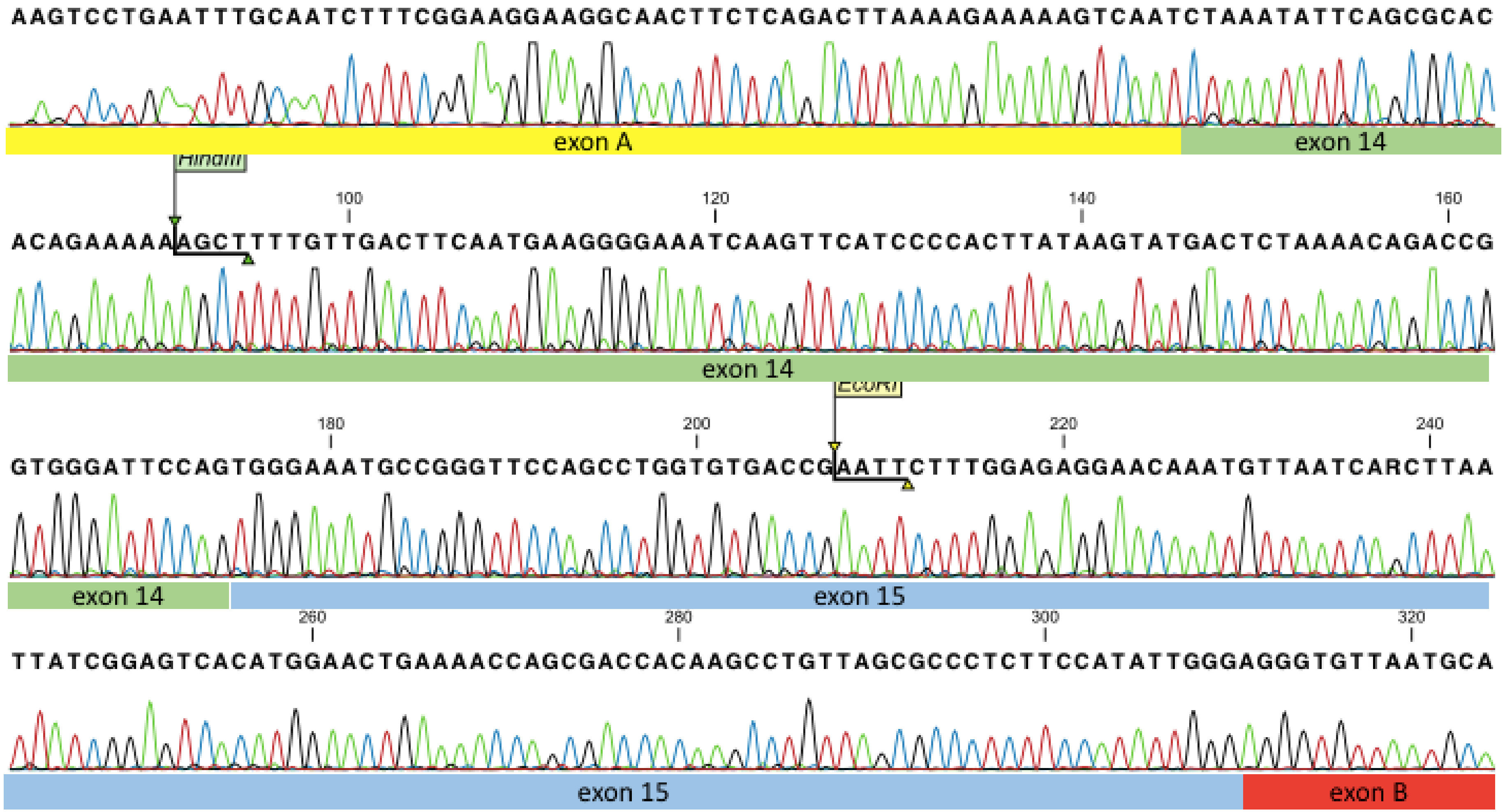

c.1466 G > A band No. 2

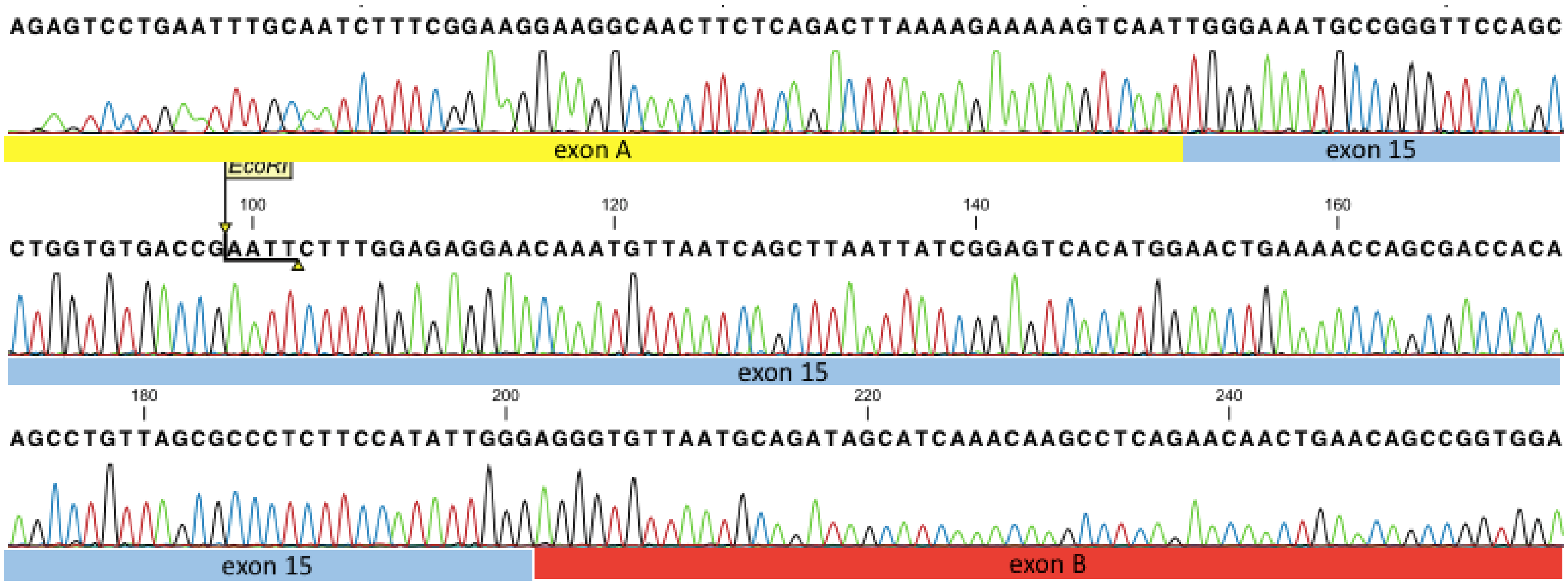

c.1466 G > A

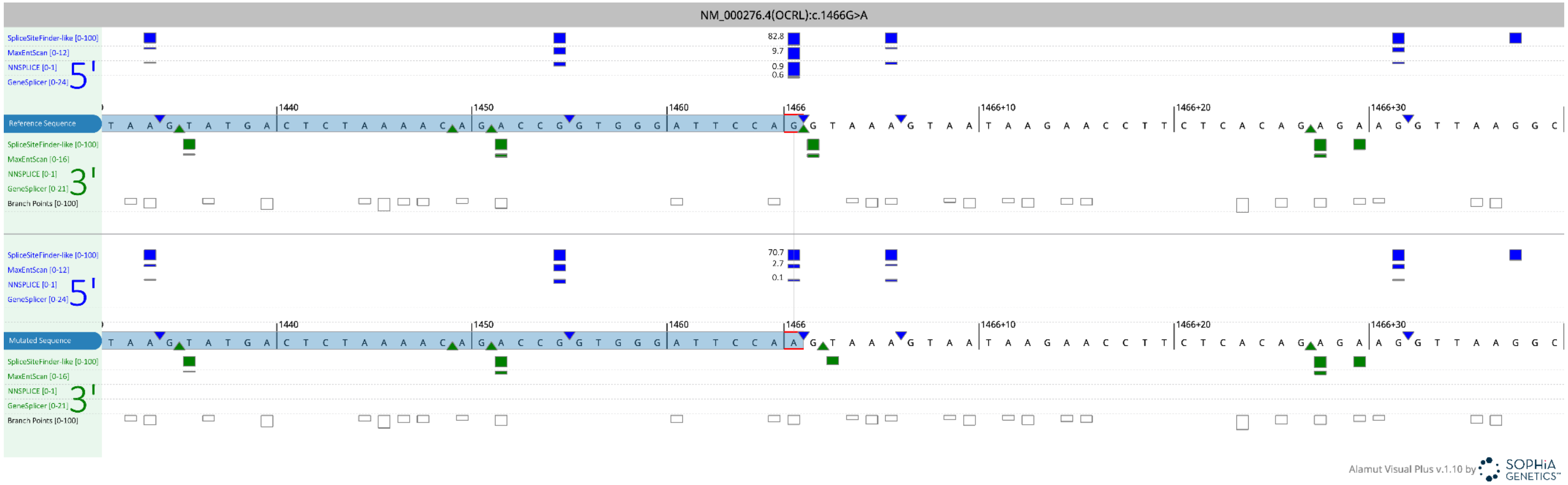

SpliceAI scores:

Variant	Gene (= MANE Select transcript; = non-coding transcript)	Δ type	Δ score	position	REF score	ALT score
NM_000276.4(OCRL):c.1466G>A<br>⇒ X:129567363 G>A<br>UCSC, gnomAD	OCRL<br>(ENSG00000122126.18 / ENST00000371113.9 / NM_000276.4)<br>protein coding MANE Select transcript (plus strand)<br>OMIM, GTEx, gnomAD, ClinGen, Ensembl, Decipher, GeneCards	Acceptor Loss	0.59	-109 bp	0.99	0.40
		Donor Loss	0.91	0 bp	0.98	0.07
		Acceptor Gain	0.00		0.00	0.00
		Donor Gain	0.08	31 bp	0.00	0.08

15. c.1467-2 A > G

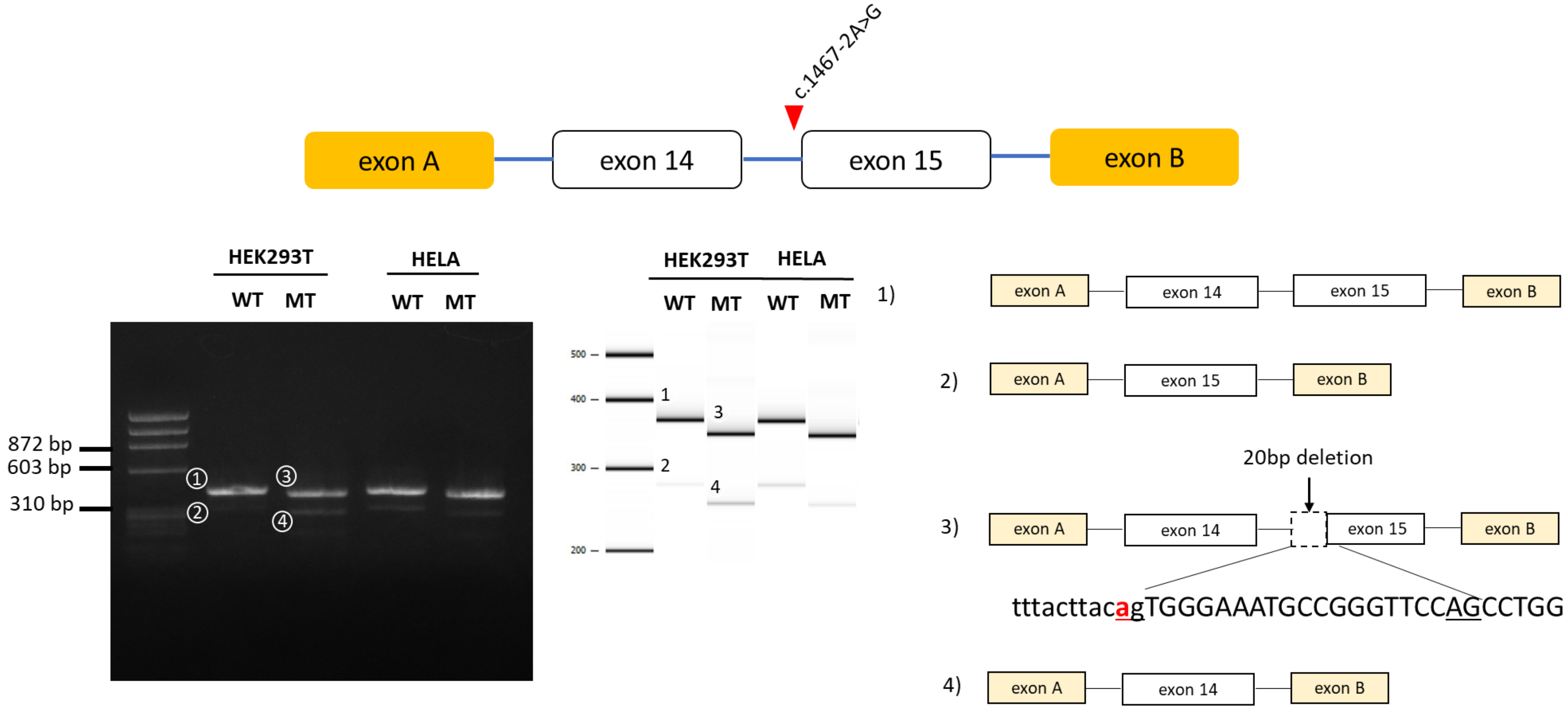

RT-PCR-amplified products of c.1467-2G>A
hybrid minigene transcripts (left :electrophoresis; right: DNA1000 assay)

c.1467-2 A > G band No. 1

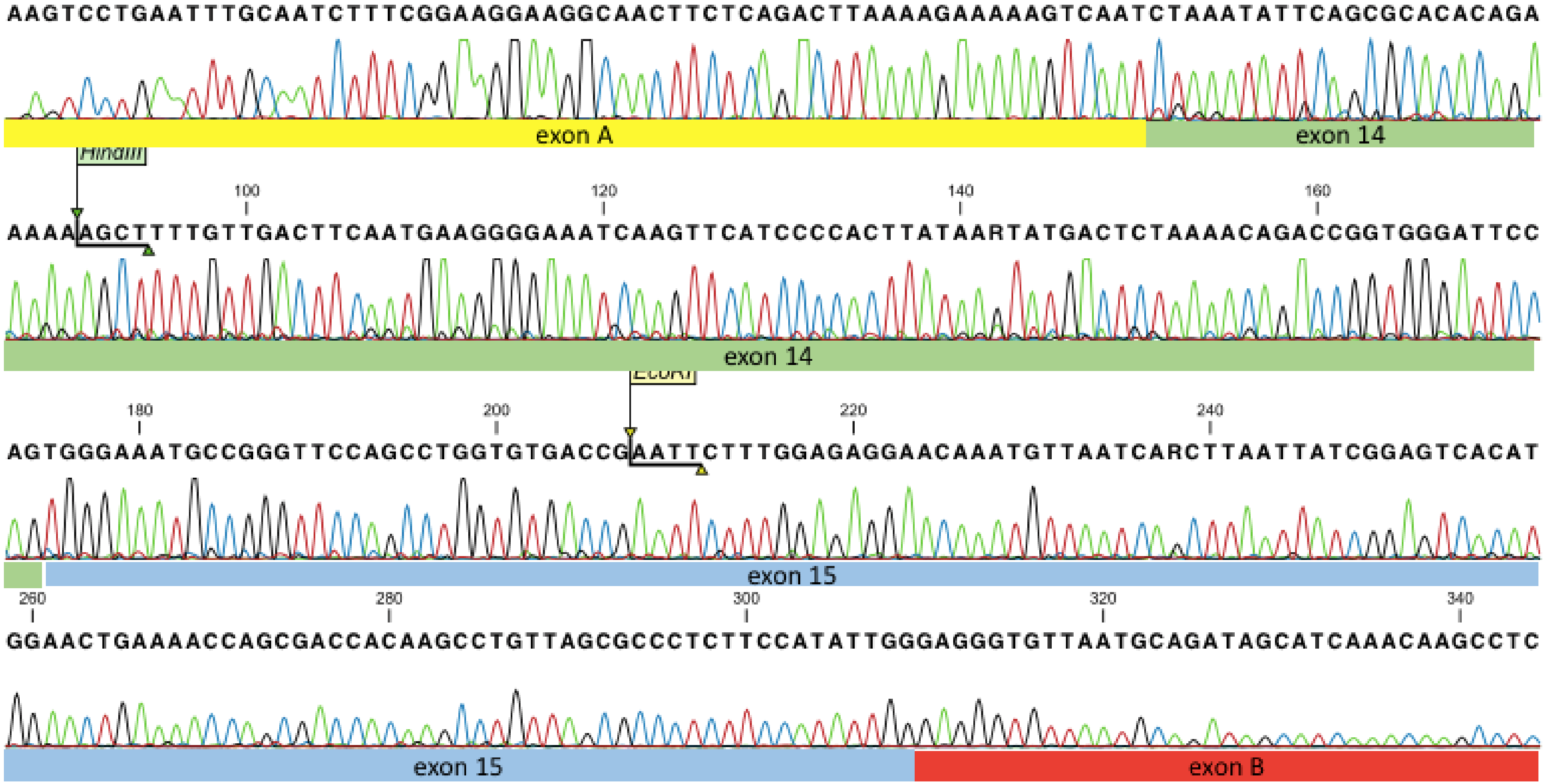

c.1467-2 A > G band No. 2

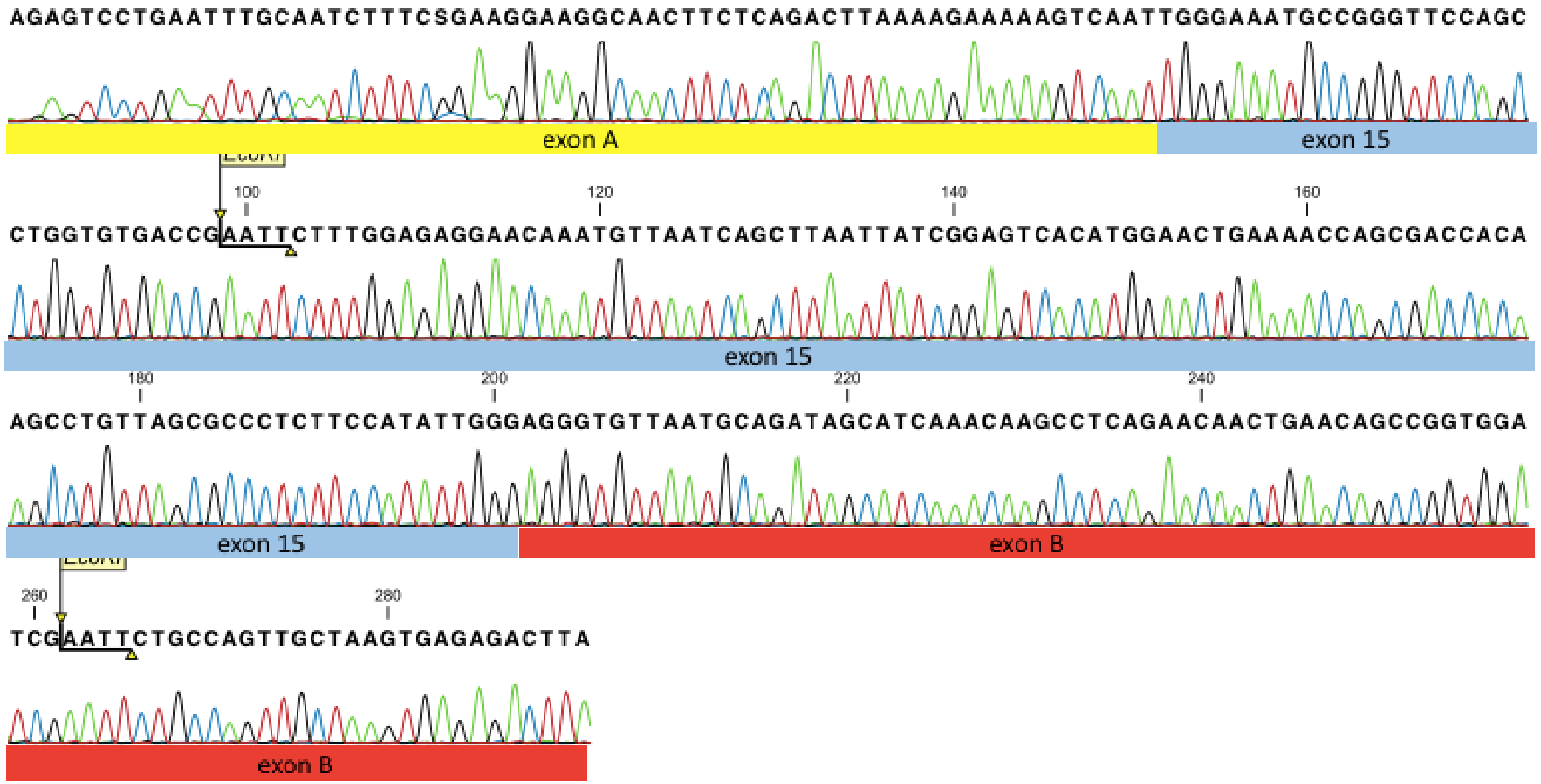

c.1467-2 A > G band No. 3

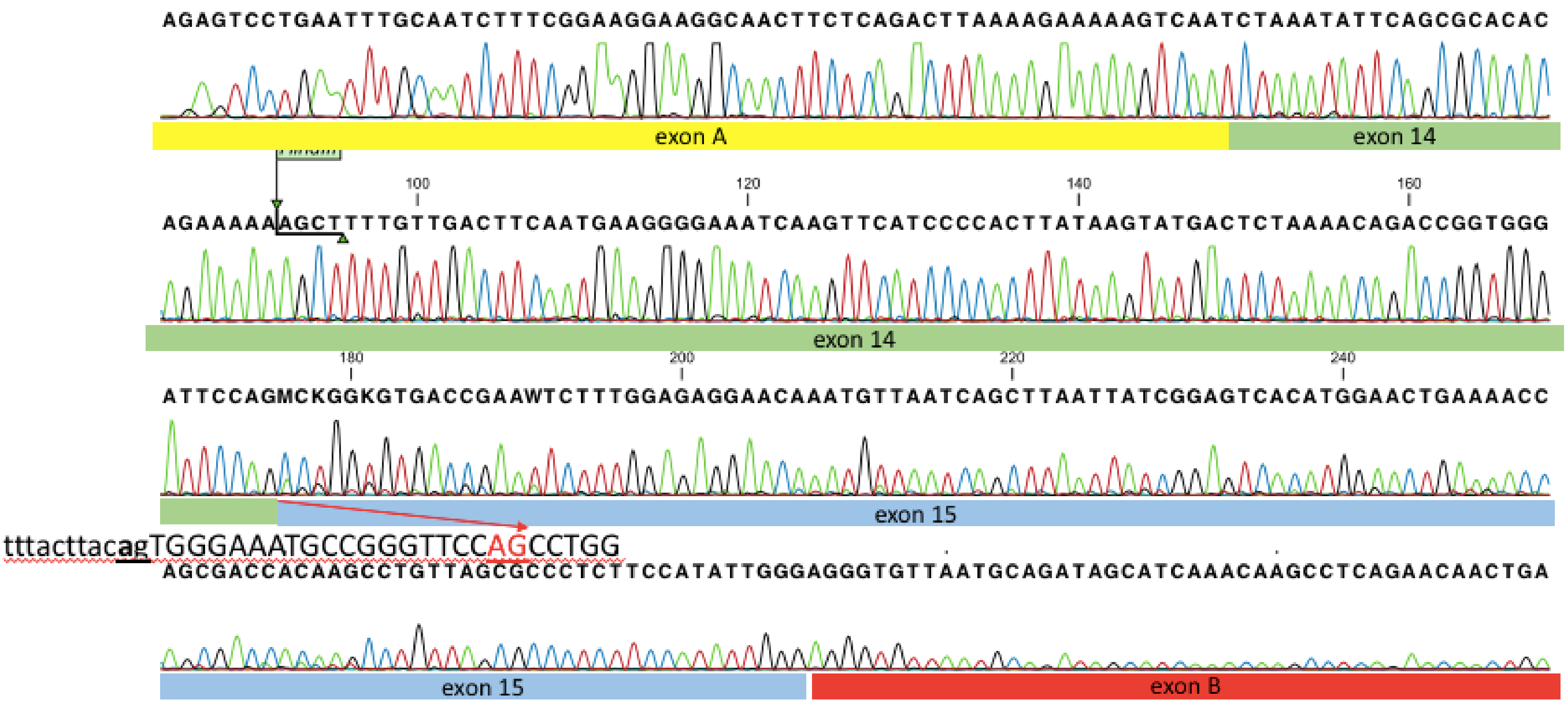

c.1467-2 A > G band No. 4

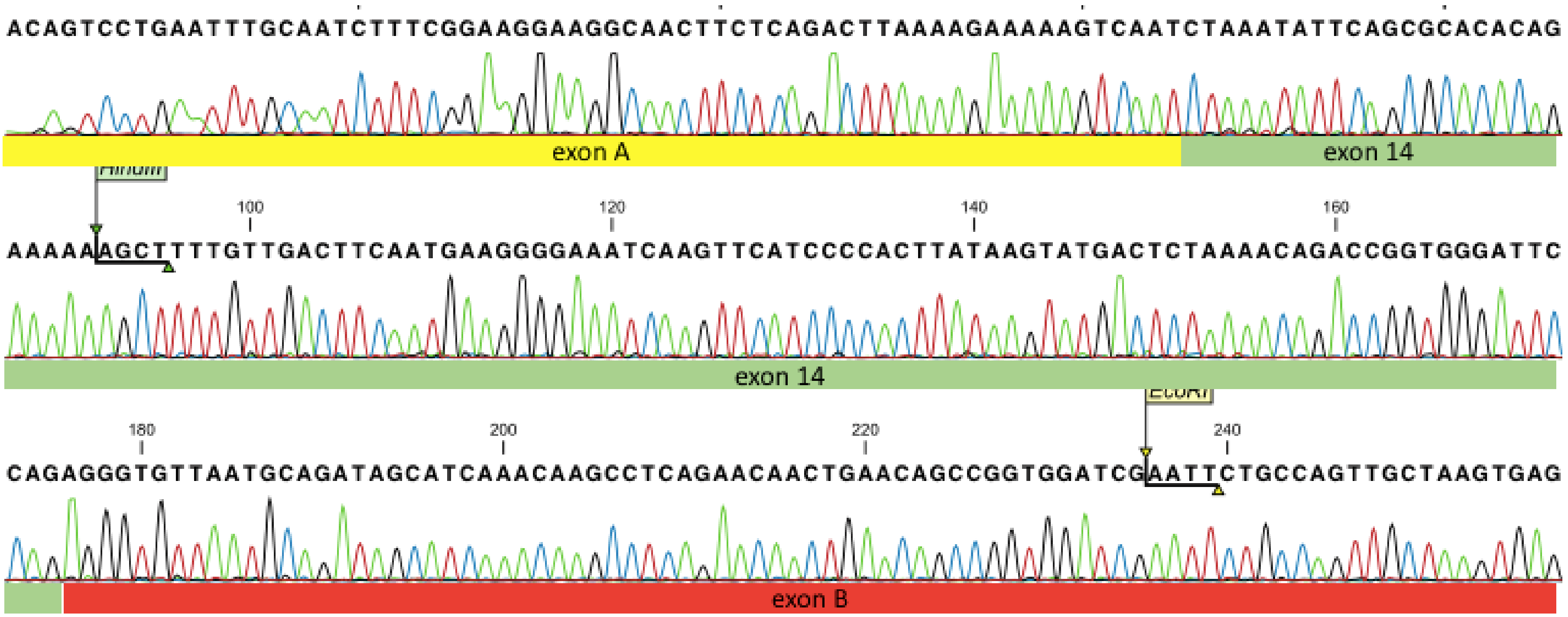

c.1467-2 A > G

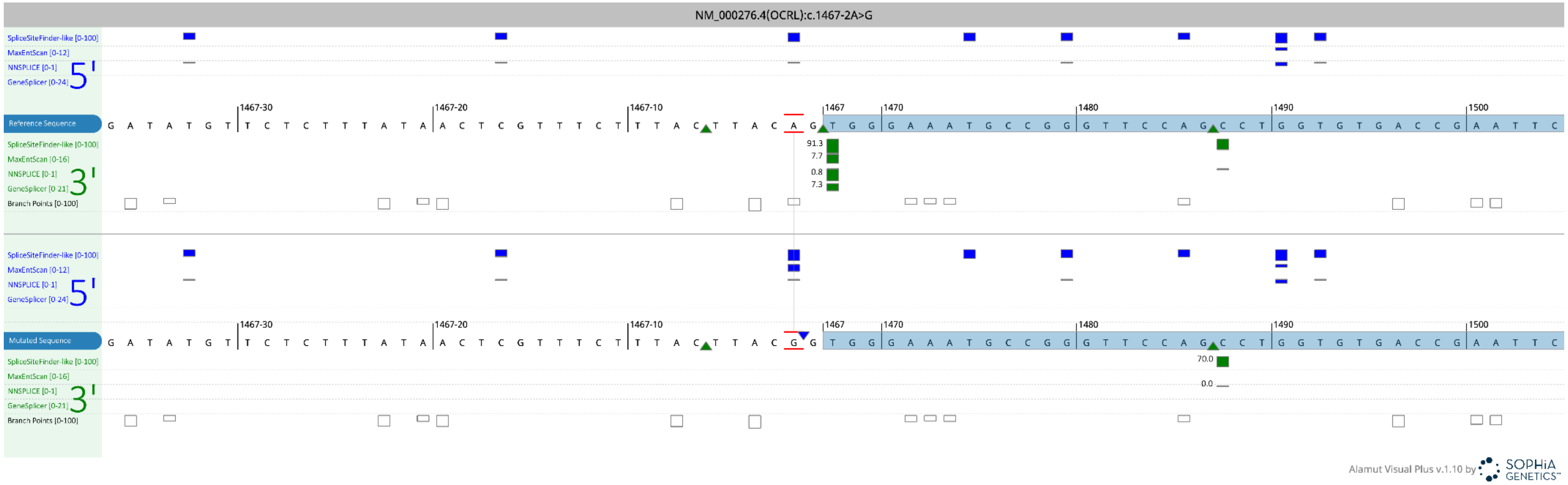

SpliceAI scores: ?

Variant	Gene (☐ = MANE Select transcript ☐ = non-coding transcript)	Δ type	Δ score	position	REF score	ALT score
NM_000276.4(OCRL):c.1467-2 A>G<br>⇒ *X:129569262 A>G*<br>UCSC, gnomAD	OCRL<br>(ENSG00000122126.18 / ENST00000371113.9 / NM_000276.4)<br>protein coding MANE Select transcript<br>(plus strand)<br>OMIM, GTEx, gnomAD, ClinGen, Ensembl, Decipher, GeneCards	Acceptor Loss	0.99	2 bp	0.99	0.00
		Donor Loss	0.18	137 bp	1.00	0.81
		Acceptor Gain	0.61	22 bp	0.00	0.62
		Donor Gain	0.02	-57 bp	0.00	0.02

16. c.1467-3 C > G

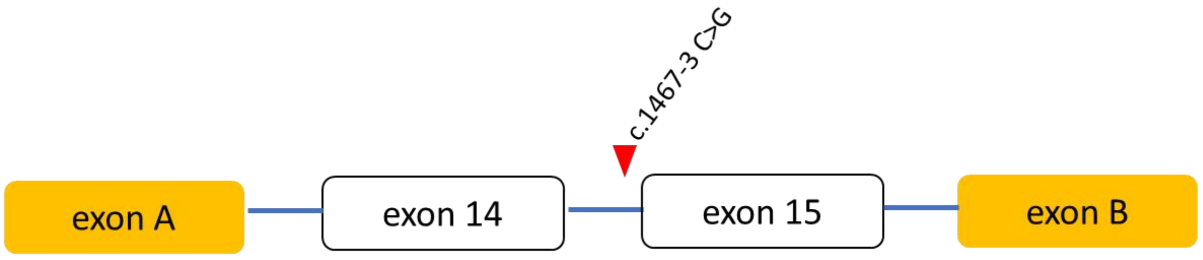

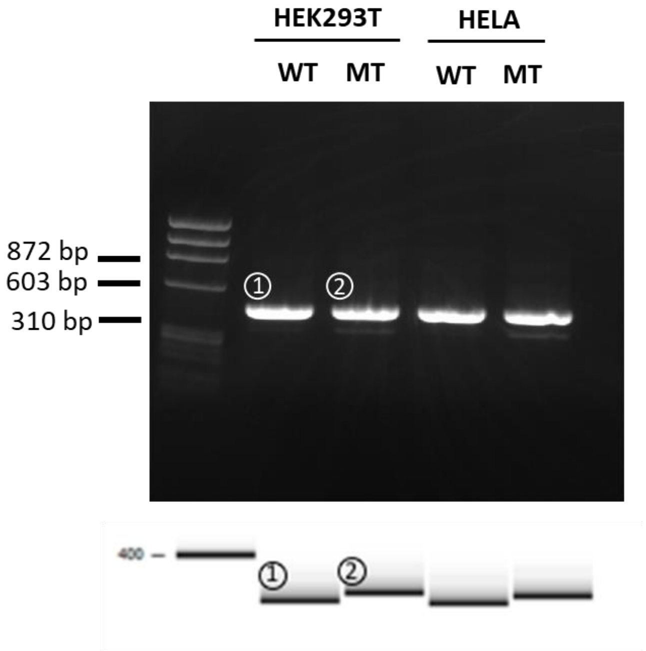

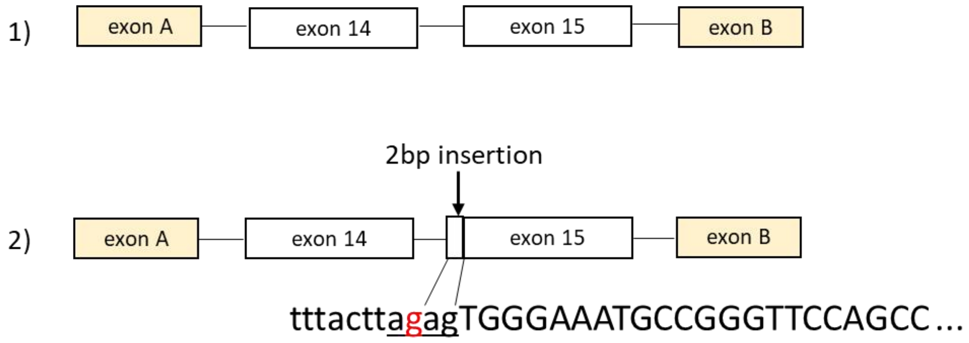

RT-PCR-amplified products of c.1467-3 C>G hybrid minigene transcripts (upper : electrophoresis; lower :DNA 1000 Assay)

c.1467-3 C > G band No. 1

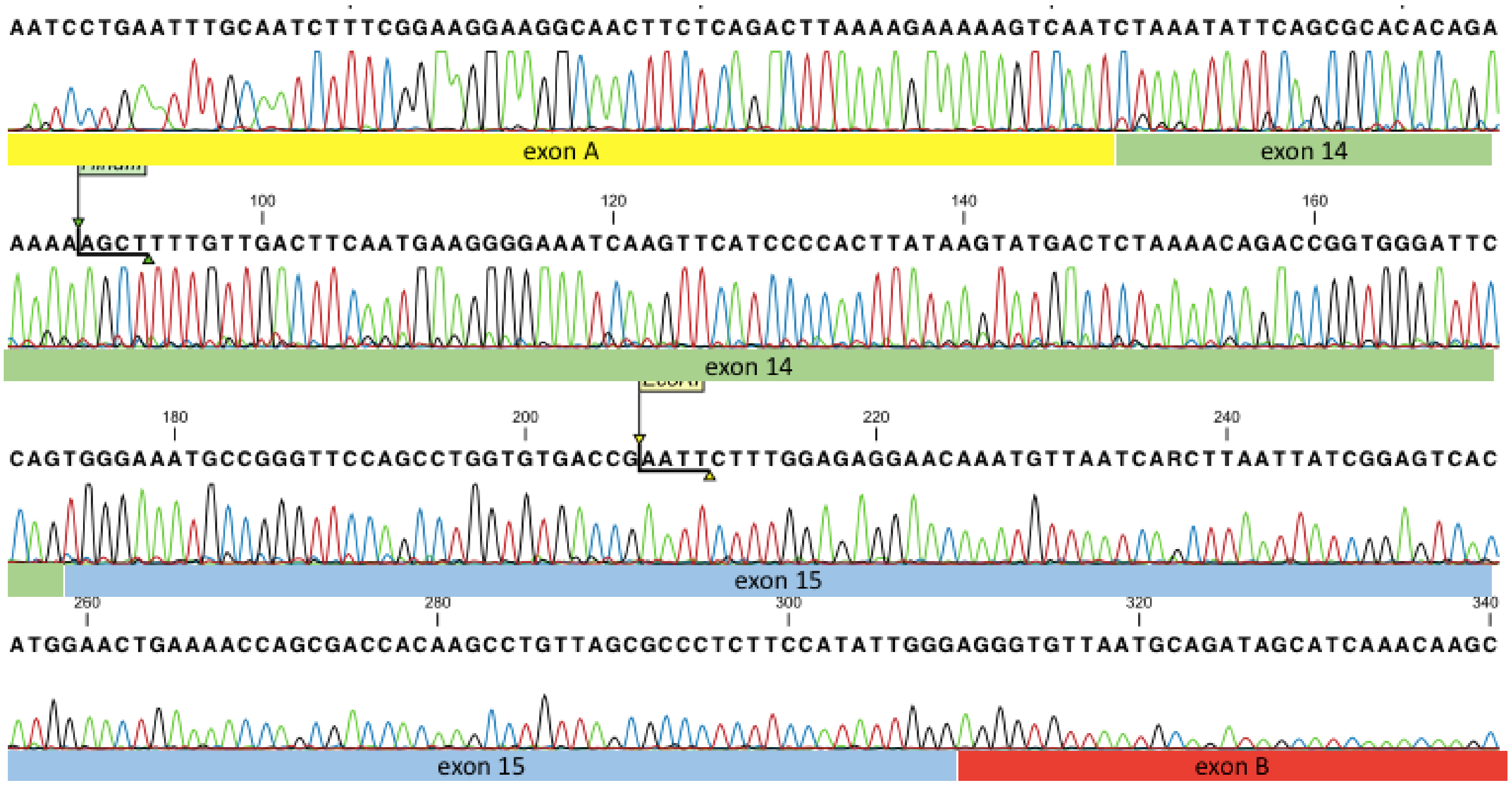

c.1467-3 C > G band No. 2

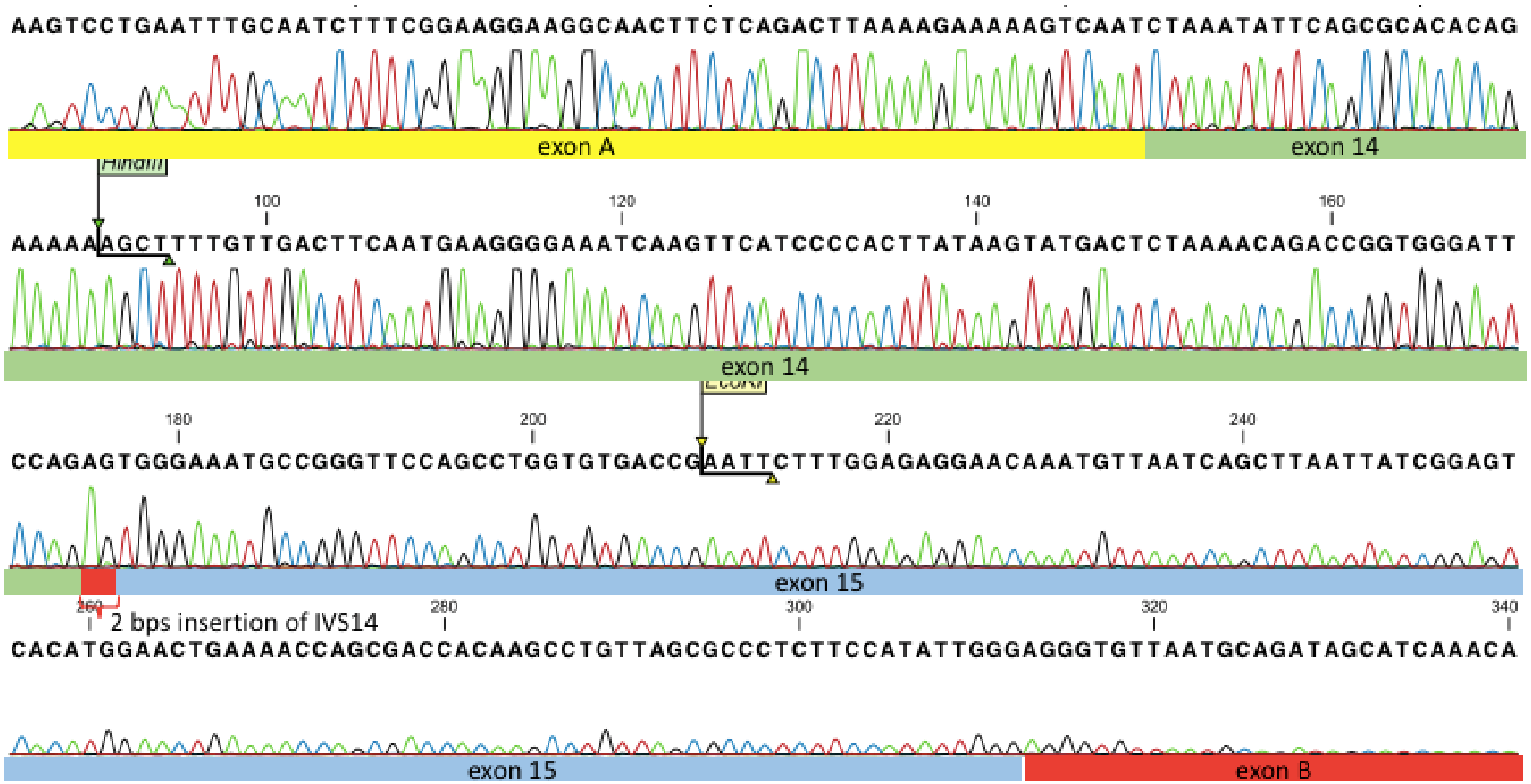

c.1467-3 C >G

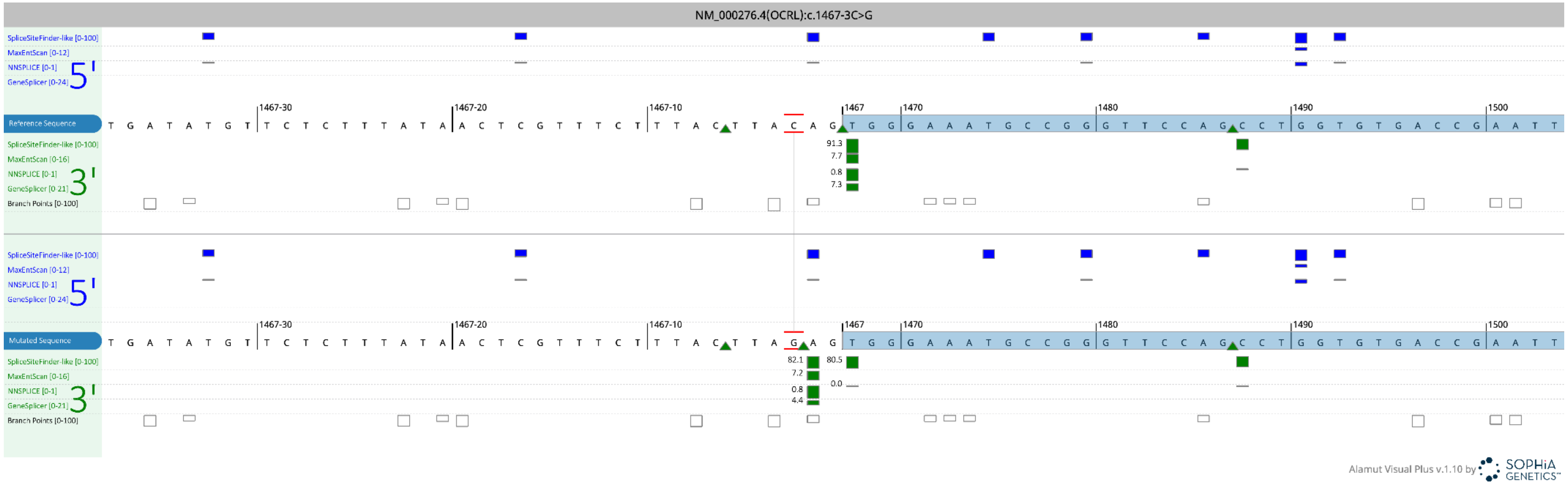

SpliceAI scores: ⓘ

Variant	Gene ☐ = MANE Select transcript ☐ = non-coding transcript	Δ type	Δ score	position	REF score	ALT score
NM_000276.4(OCRL):c.1467-3C>G<br>⇒ *X:129569261 C>G*<br>UCSC, gnomAD	OCRL<br>(ENSG00000122126.18 / ENST00000371113.9 / NM_000276.4)<br>protein coding MANE Select transcript (plus strand)<br>OMIM, GTEx, gnomAD, ClinGen, Ensembl, Decipher, GeneCards	Acceptor Loss	0.99	3 bp	0.99	0.01
		Donor Loss	0.01	148 bp	0.06	0.04
		Acceptor Gain	0.98	1 bp	0.00	0.98
		Donor Gain	0.00	-56 bp	0.00	0.01

17. c.1602+1 G > A

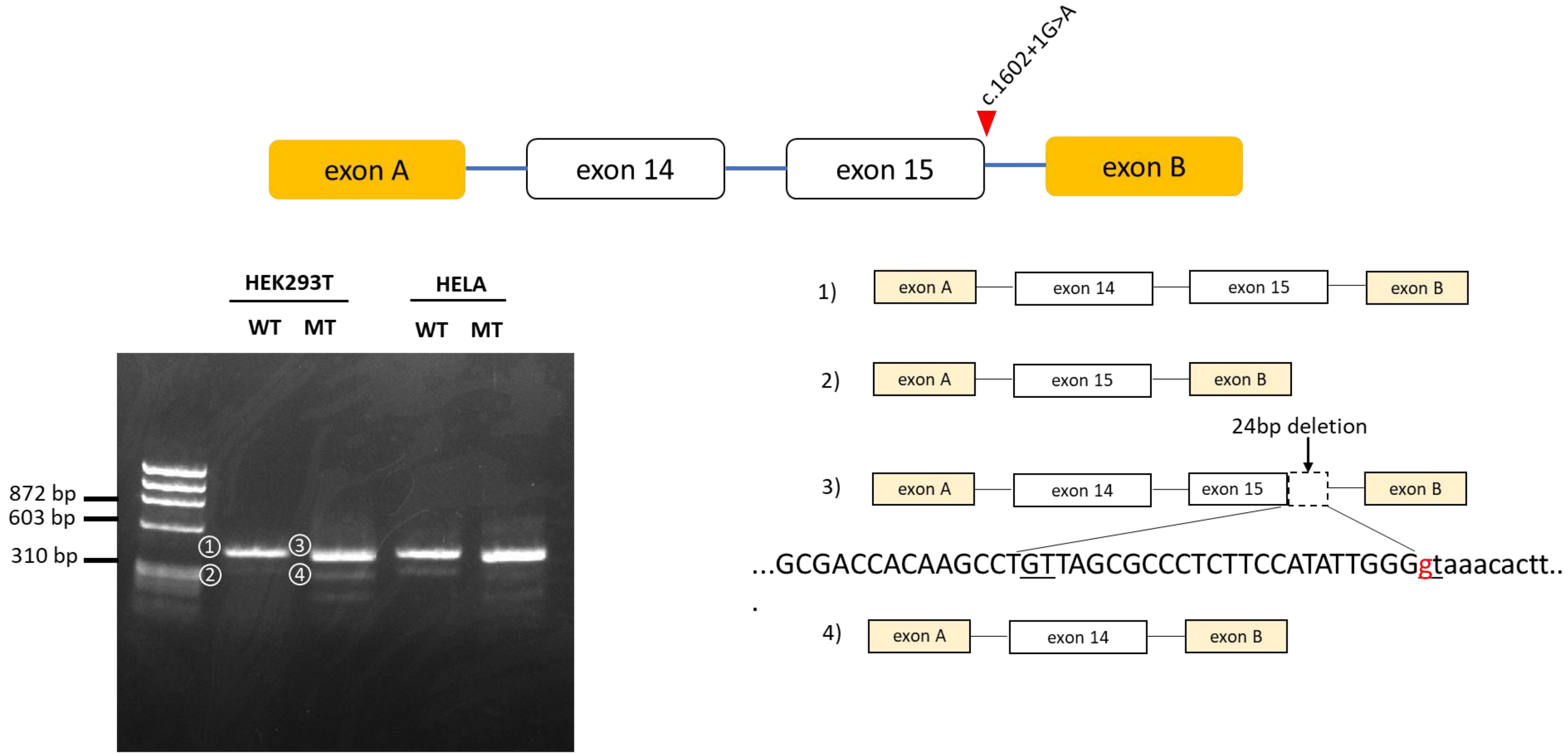

RT-PCR-amplified products of c.1602+1G>A hybrid minigene transcripts

c.1602+1 G > A band No. 1

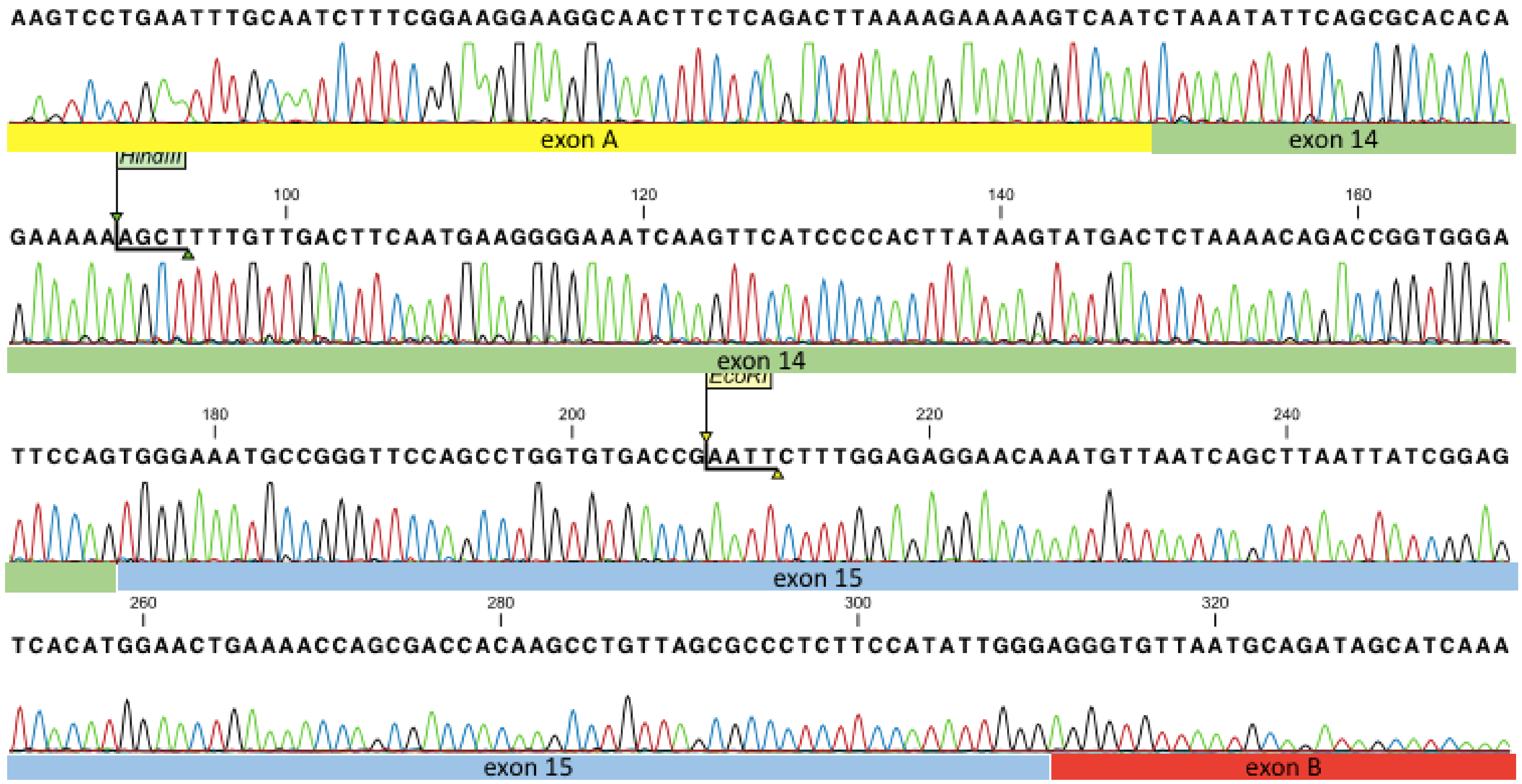

c.1602+1 G > A band No. 2

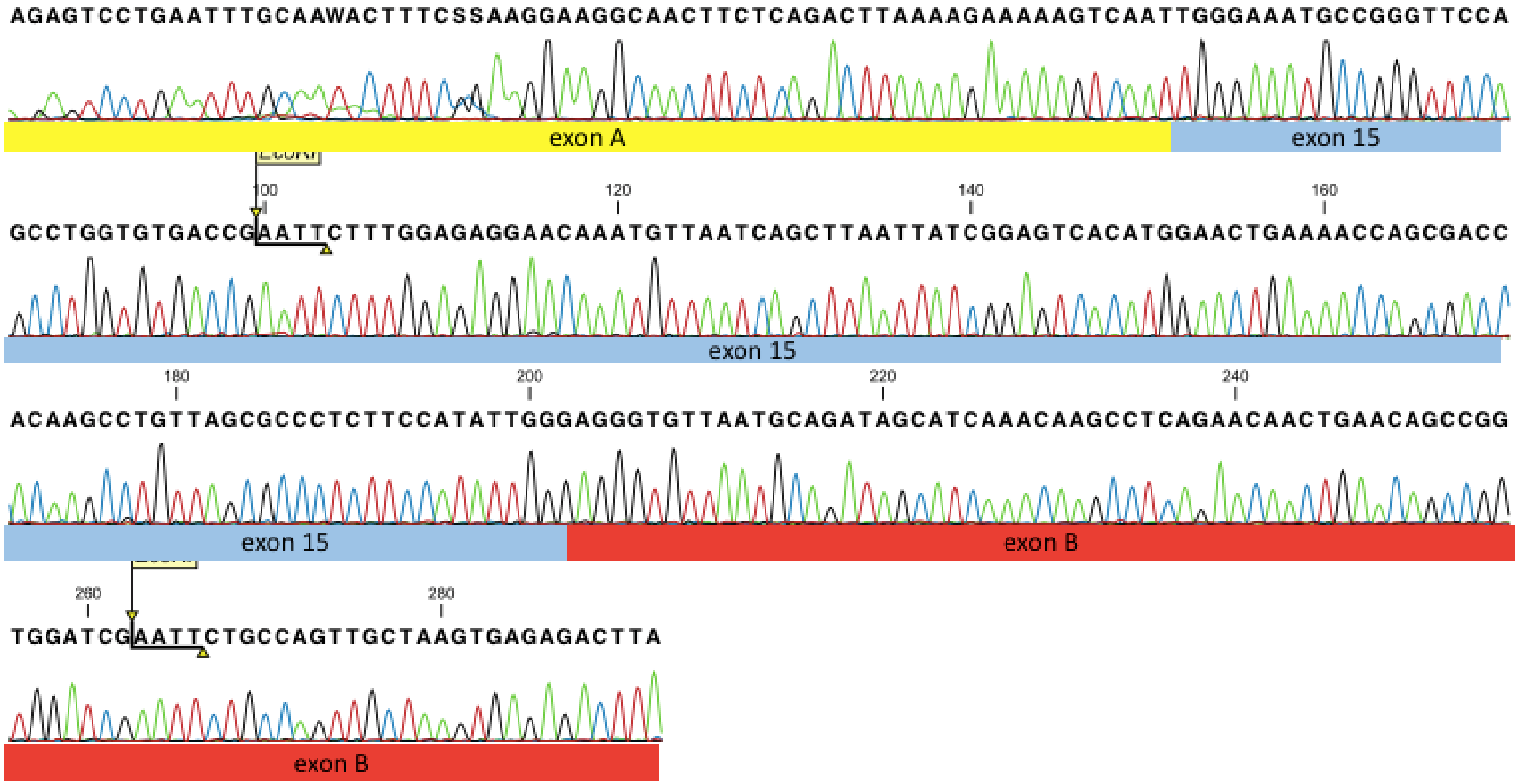

c.1602+1 G > A band No. 3

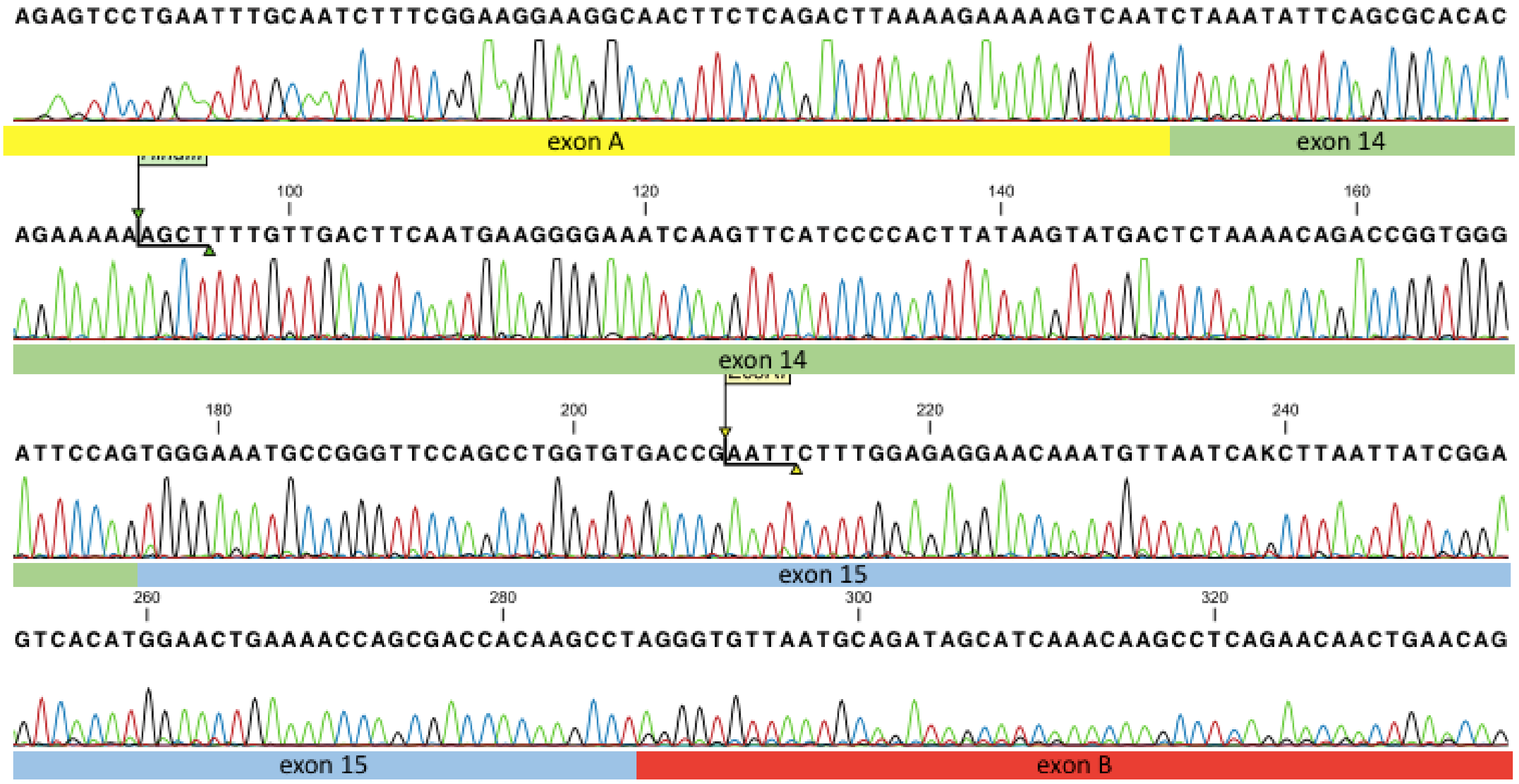

...GCGACCACAAGCCTgttagcgccctcttccatattggggtaaacactt...

c.1602+1 G > A band No. 4

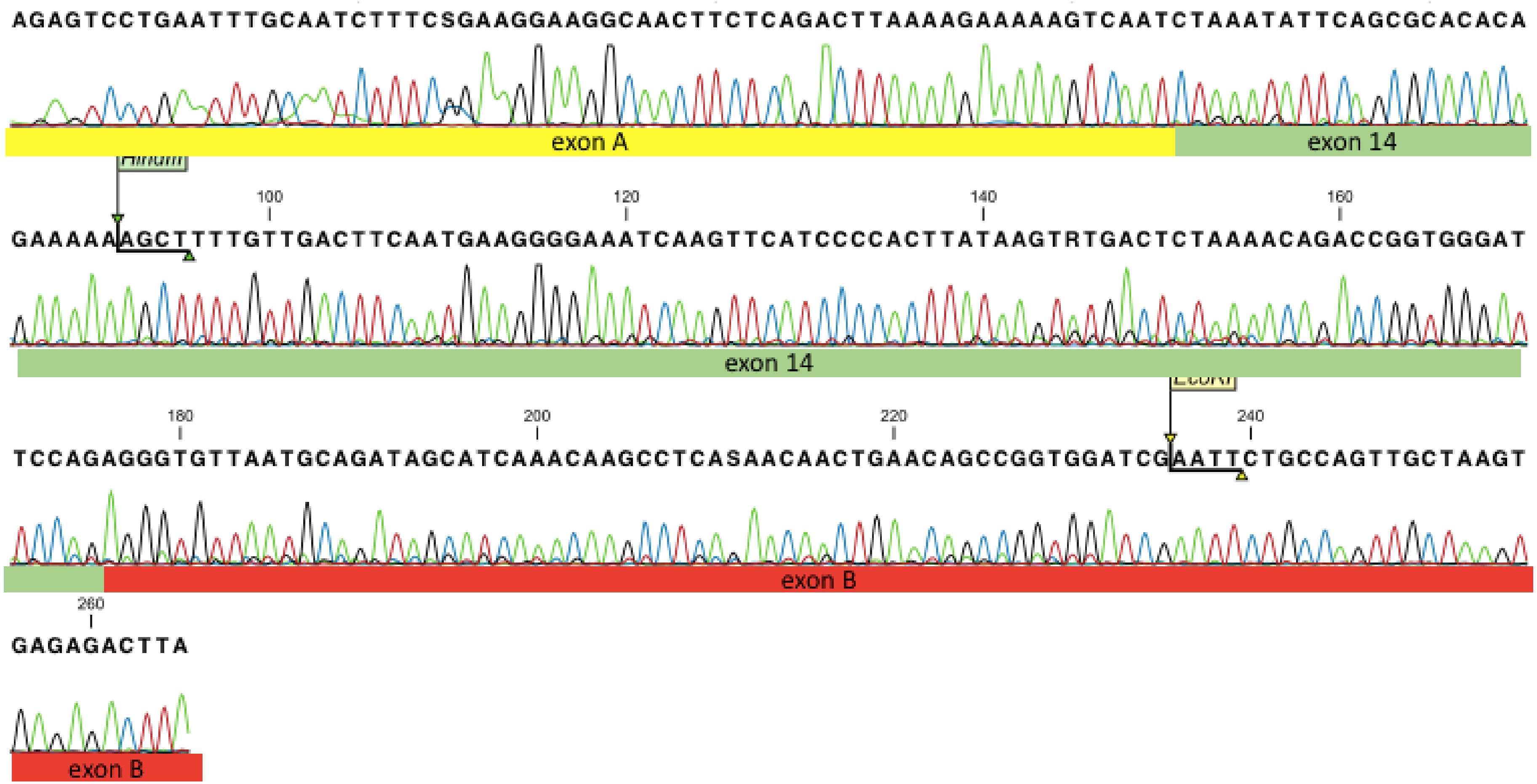

c.1602+1 G > A

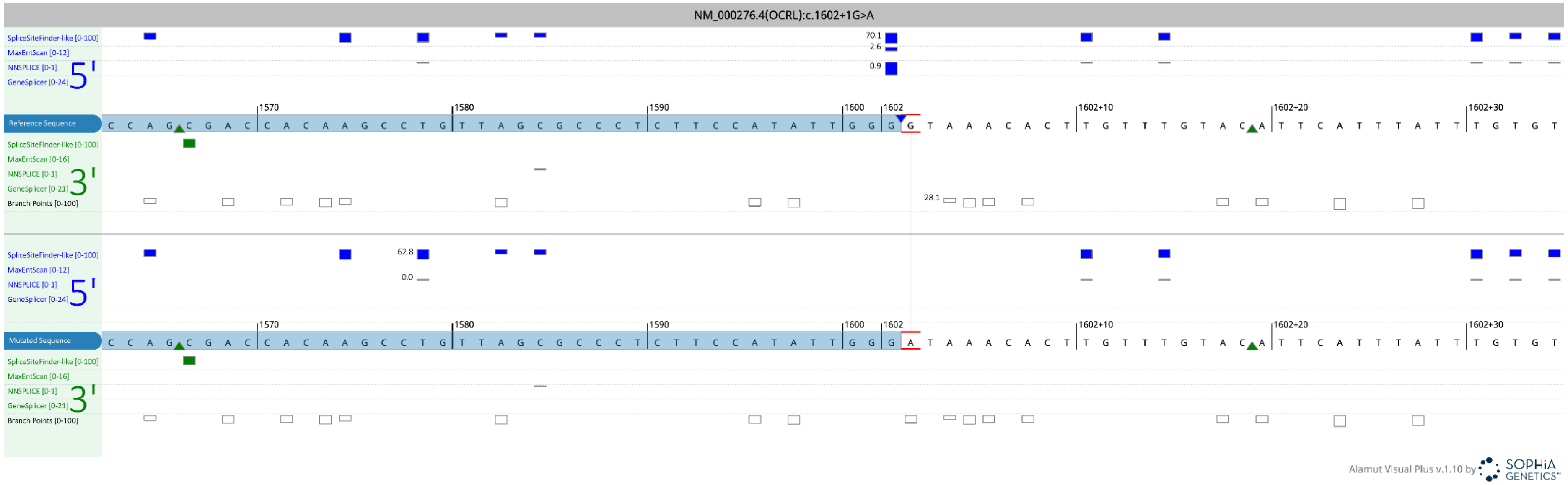

SpliceAI scores:

Variant	Gene (□ = MANE Select transcript, □ = non-coding transcript)	Δ type	Δ score	position	REF score	ALT score
NM_000276.4(OCRL):c.1602+1G>A<br>⇒ *X:129569400 G>A*<br>UCSC, gnomAD	OCRL<br>(ENSG00000122126.18 / ENST00000371113.9 / NM_000276.4)<br>protein coding MANE Select transcript (plus strand)<br>OMIM, GTEx, gnomAD, ClinGen, Ensembl, Decipher, GeneCards	Acceptor Loss	0.09	-136 bp	0.99	0.90
		Donor Loss	0.99	-1 bp	1.00	0.00
		Acceptor Gain	0.00		0.00	0.00
		Donor Gain	0.51	9 bp	0.06	0.57

18. c.1603-2 A > C

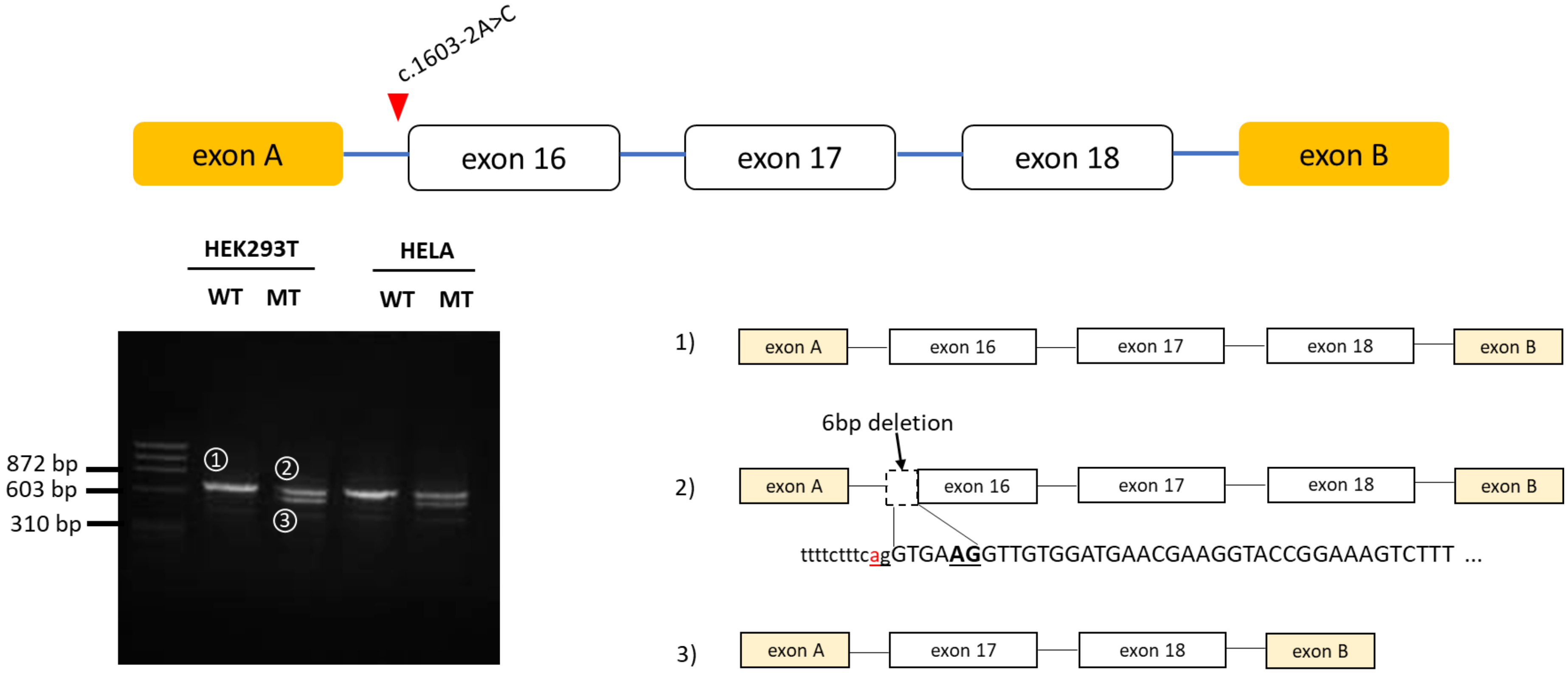

RT-PCR-amplified products of c.1603-2 A>C hybrid minigene transcripts

c.1603-2 A > C band No. 1

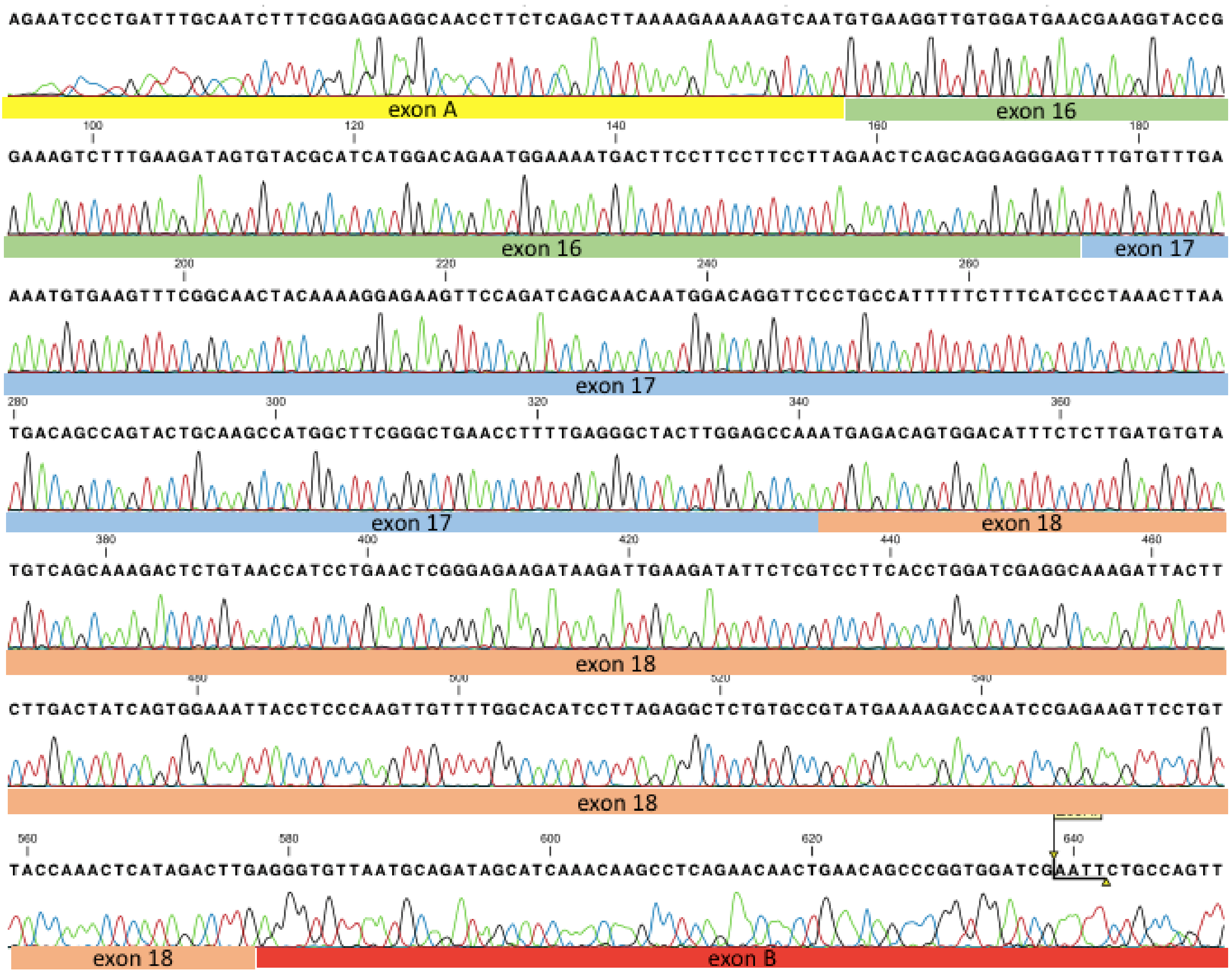

c.1603-2 A > C band No. 2

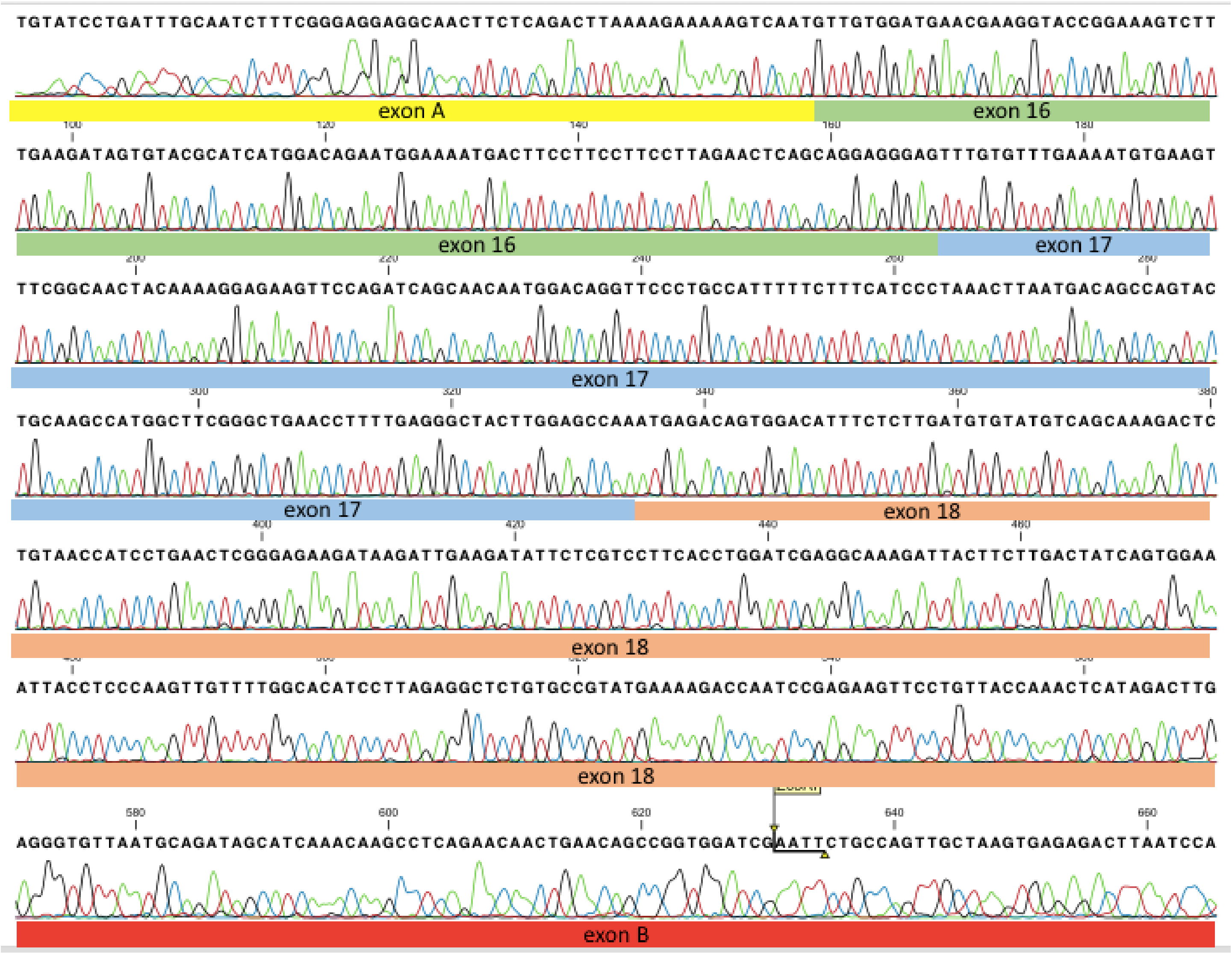

c.1603-2 A > C band No. 3

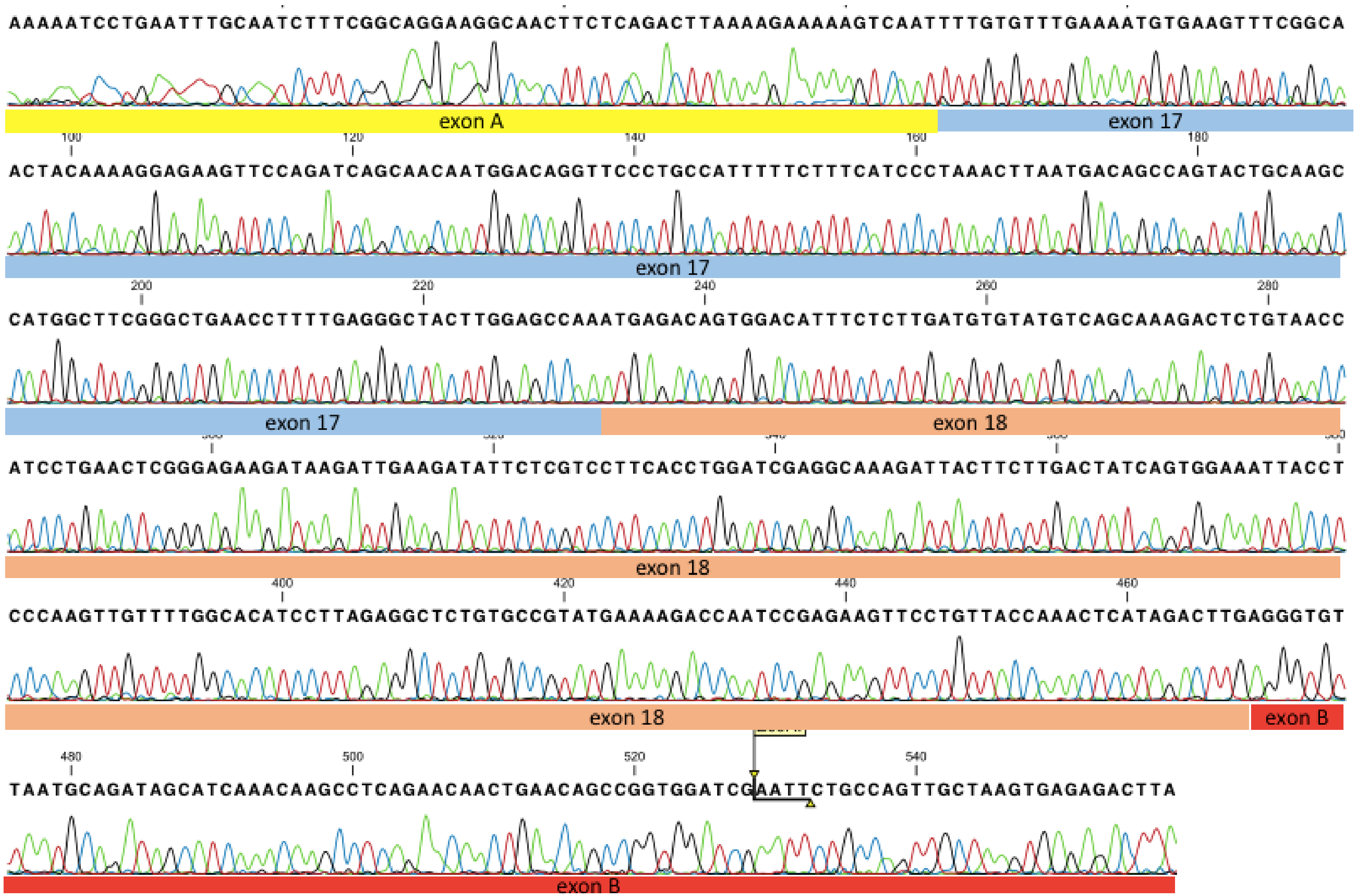

c.1603-2 A > C

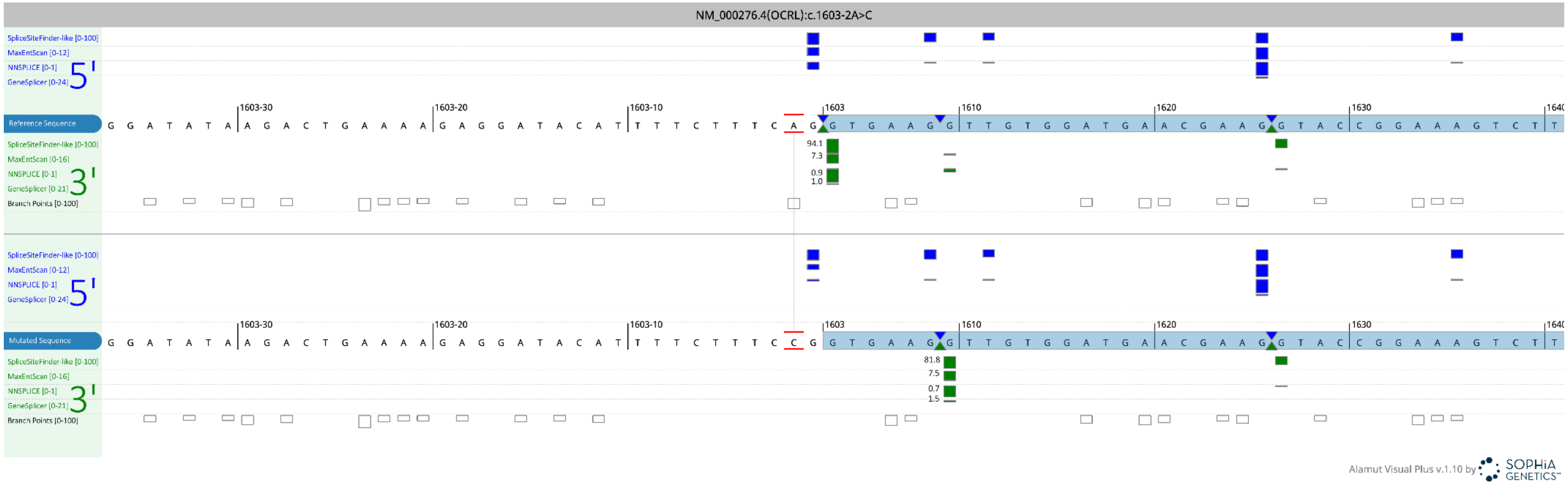

SpliceAI scores:

Variant	Gene (= MANE Select transcript; = non-coding transcript)	Δ type	Δ score	position	REF score	ALT score
NM_000276.4(OCRL):c.1603-2 A>C<br>⇒ *X:129575138 A>C*<br>UCSC, gnomAD	OCRL<br>(ENSG00000122126.18 / ENST00000371113.9 / NM_000276.4)<br>protein coding MANE Select transcript<br>(plus strand)<br>OMIM, GTEx, gnomAD, ClinGen, Ensembl, Decipher, GeneCards	Acceptor Loss	1.00	2 bp	1.00	0.00
		Donor Loss	0.03	-88 bp	0.04	0.01
		Acceptor Gain	0.82	8 bp	0.04	0.86
		Donor Gain	0.01	24 bp	0.01	0.02

19. c.1714-2 A > G

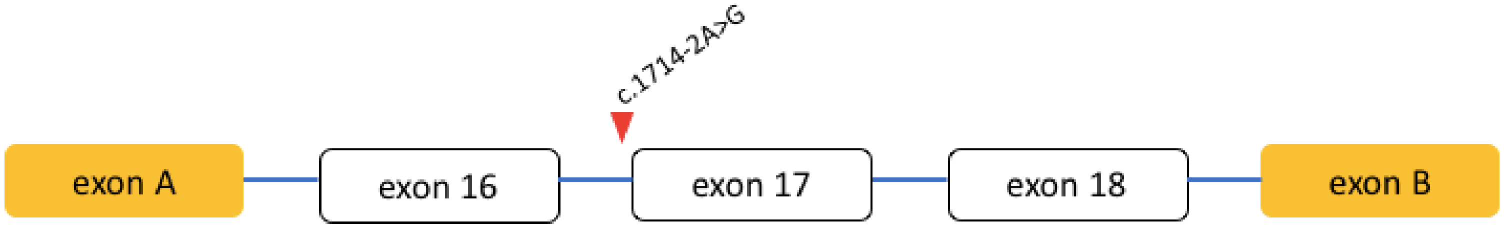

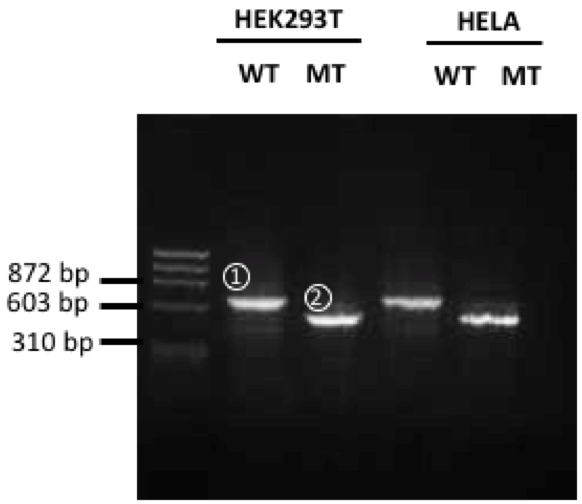

RT-PCR-amplified products of c.1714-2 A>G hybrid minigene transcripts

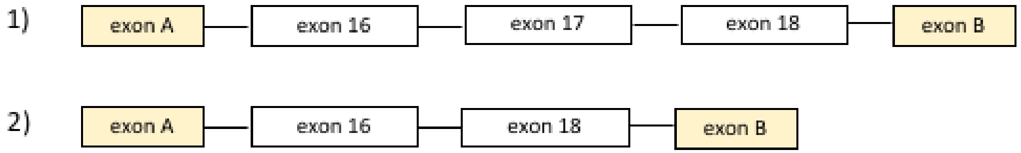

c.1714-2 A > G band No. 1

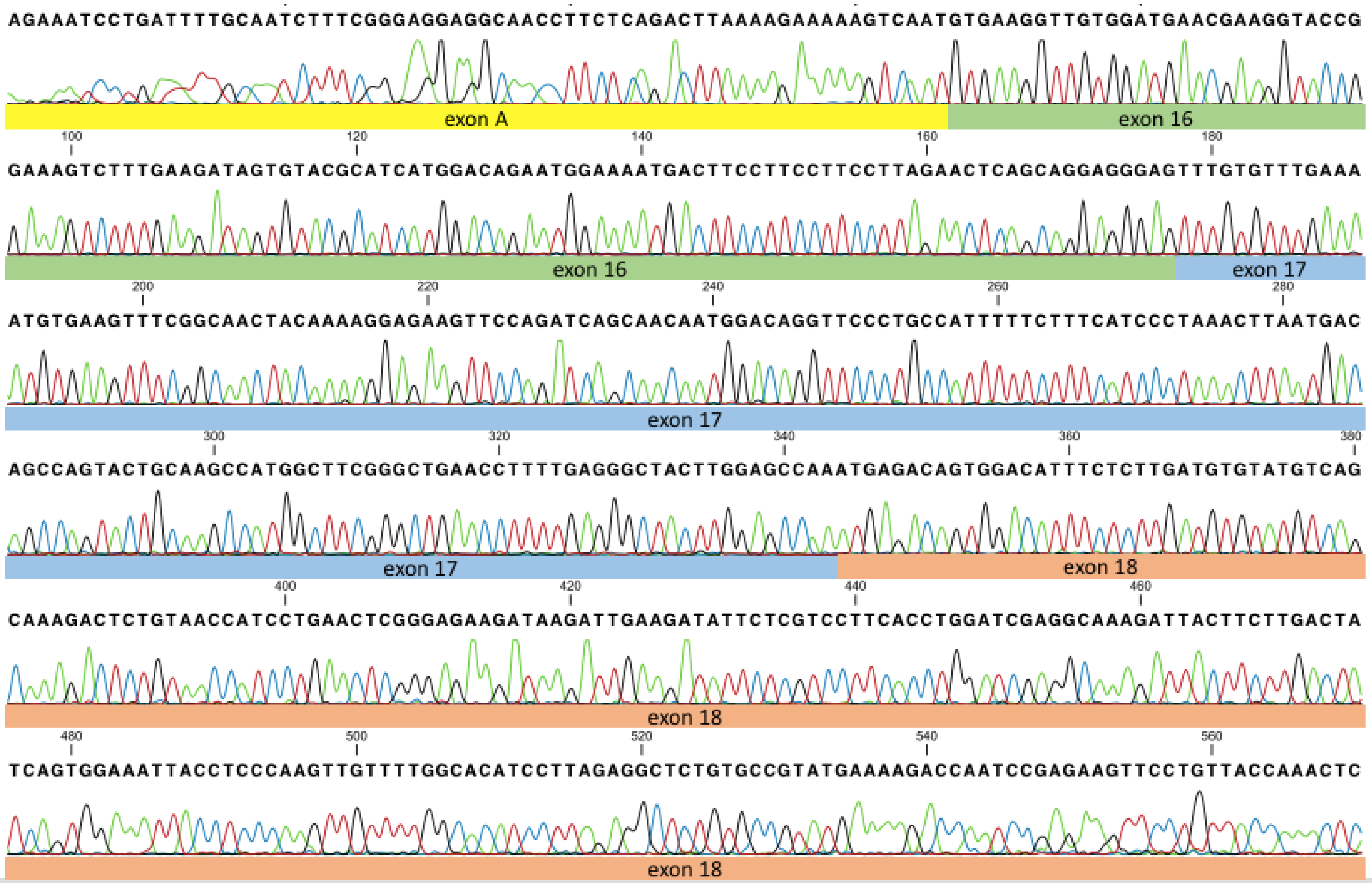

c.1714-2 A > G band No. 2

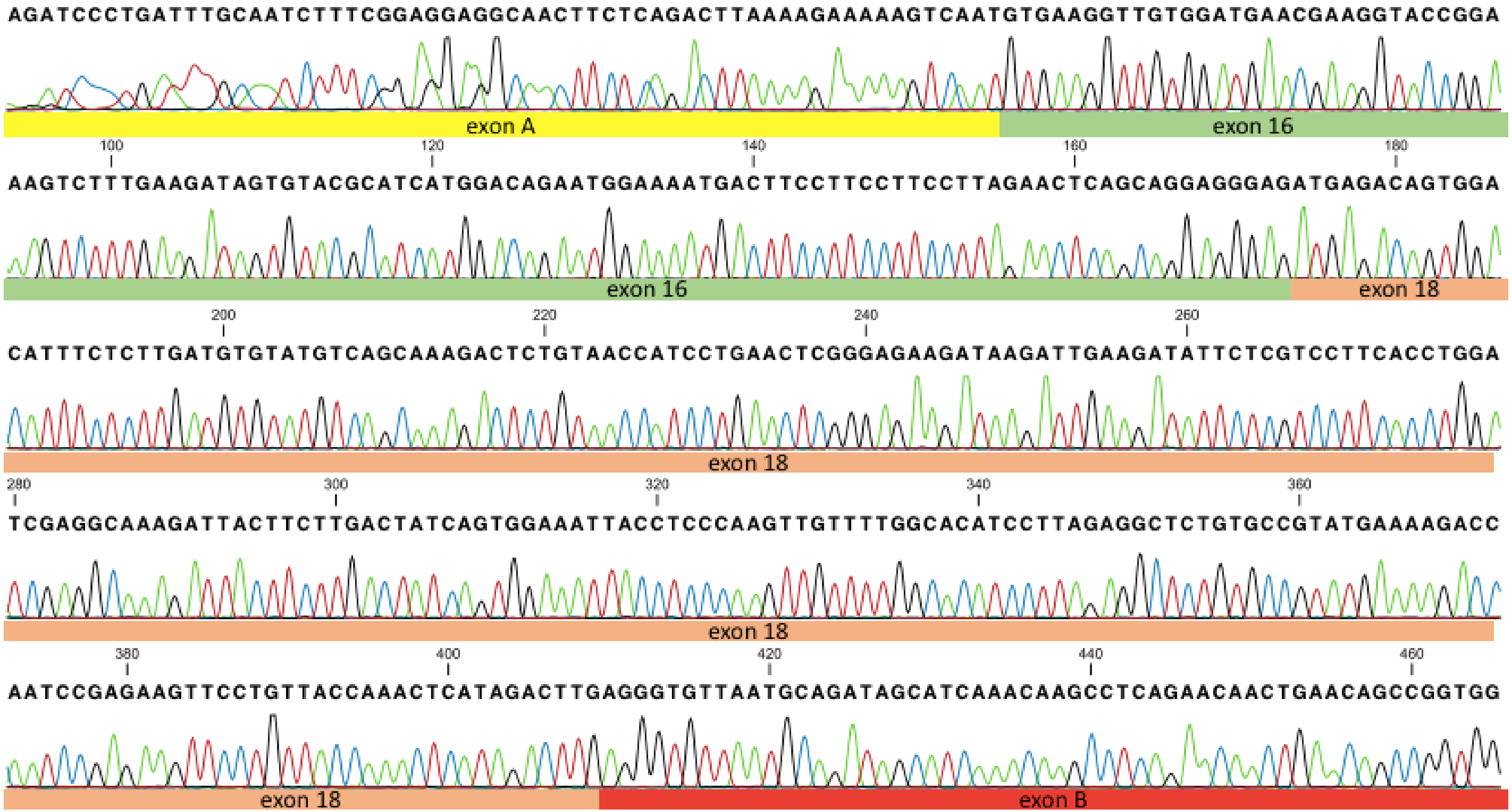

c.1714-2 A > G

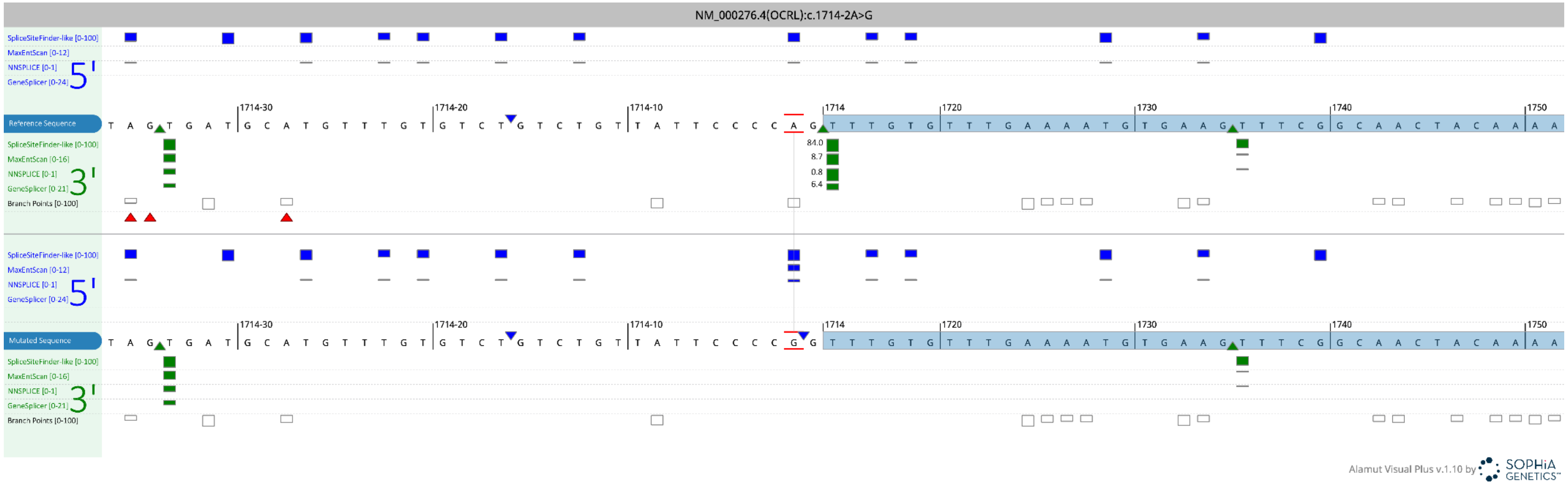

SpliceAI scores:

Variant	Gene (= MANE Select transcript; = non-coding transcript)	Δ type	Δ score	position	REF score	ALT score
NM_000276.4(OCRL):c.1714-2A>G<br>⇒ *X:129575895 A>G*<br>UCSC, gnomAD	OCRL<br>(ENSG00000122126.18 / ENST00000371113.9 / NM_000276.4)<br>protein coding MANE Select transcript (plus strand)<br>OMIM, GTEx, gnomAD, ClinGen, Ensembl, Decipher, GeneCards	Acceptor Loss	1.00	2 bp	1.00	0.00
		Donor Loss	0.12	167 bp	0.99	0.87
		Acceptor Gain	0.92	23 bp	0.00	0.92
		Donor Gain	0.03	-176 bp	0.09	0.12

20. c.1879+5 G > A

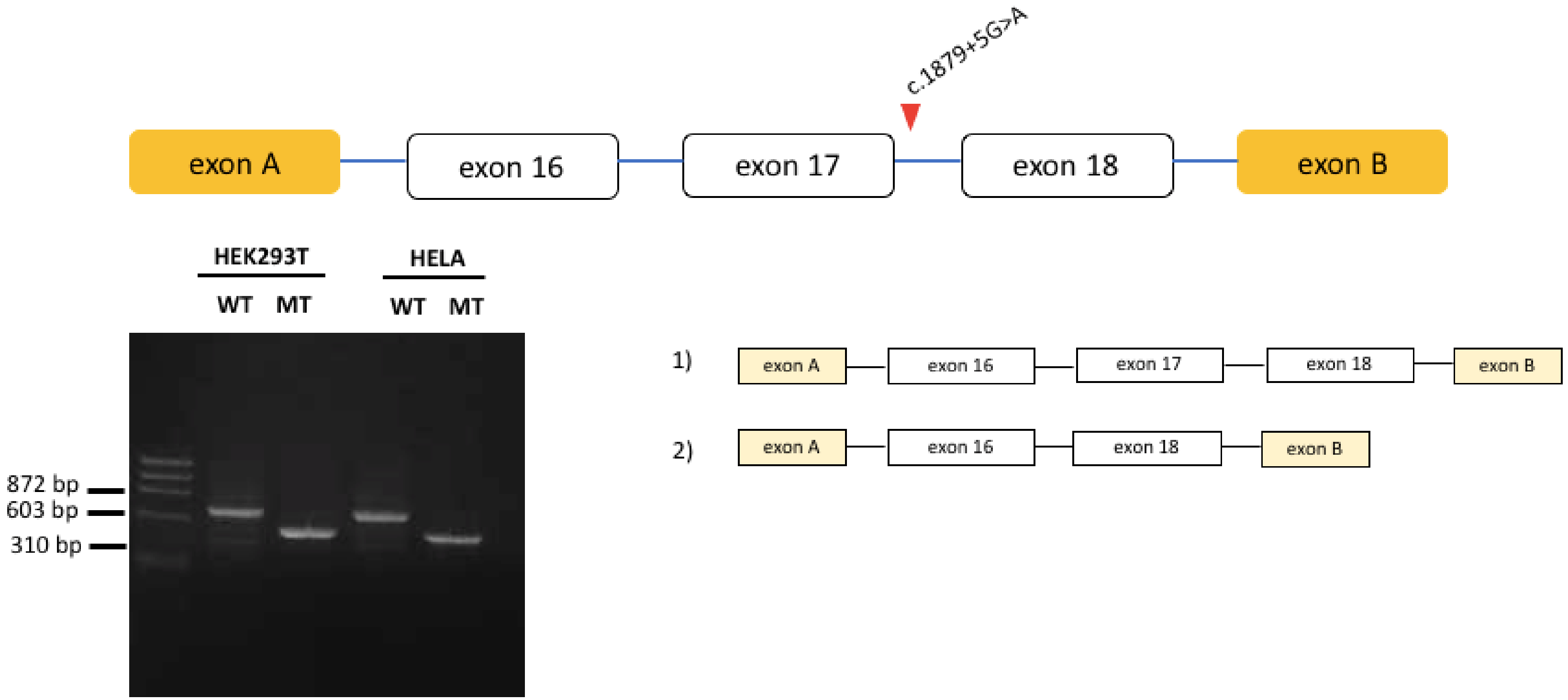

RT-PCR-amplified products of c.1879+5 G>A hybrid minigene transcripts

c.1879+5 G > A band No. 1

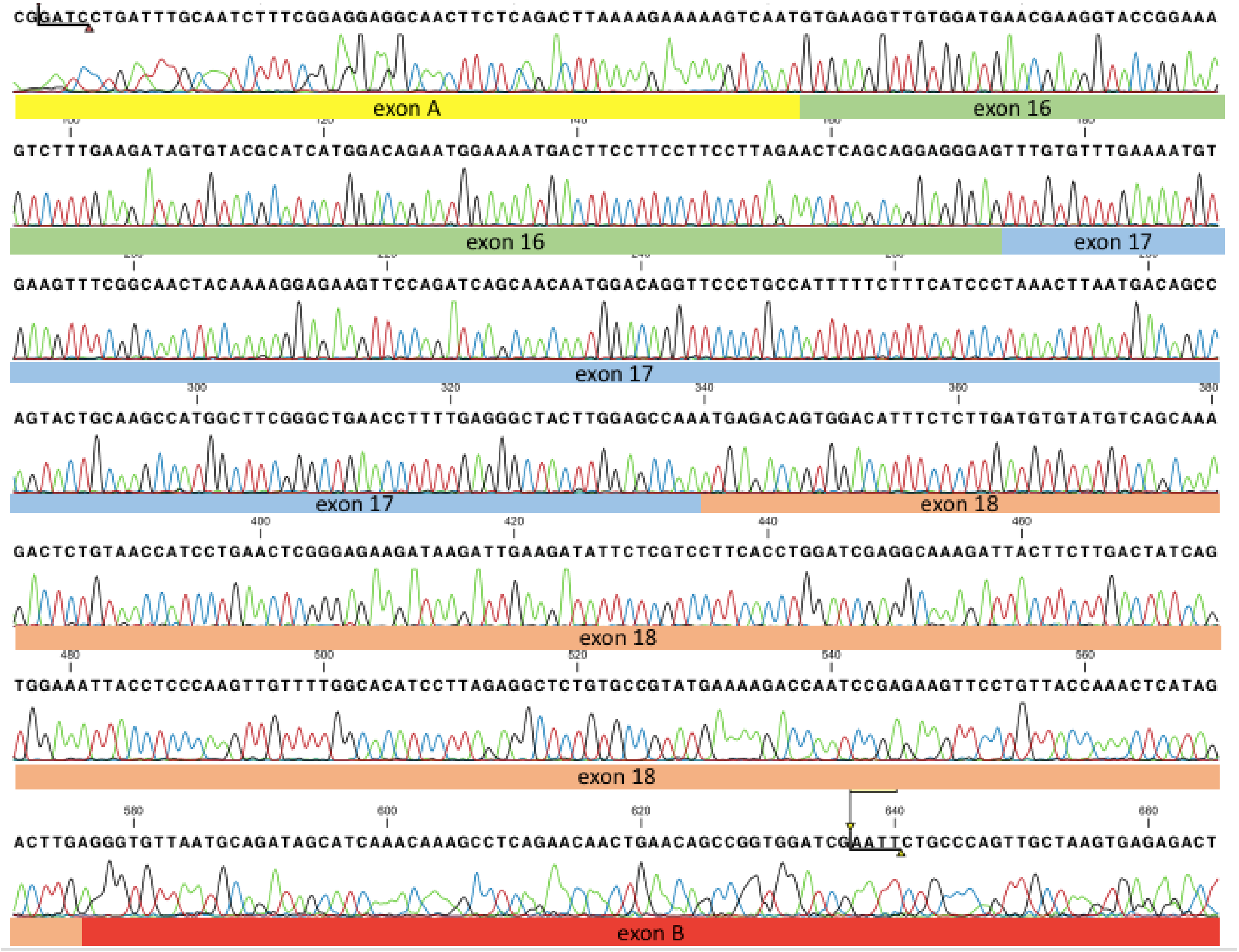

c.1879+5 G > A band No. 2

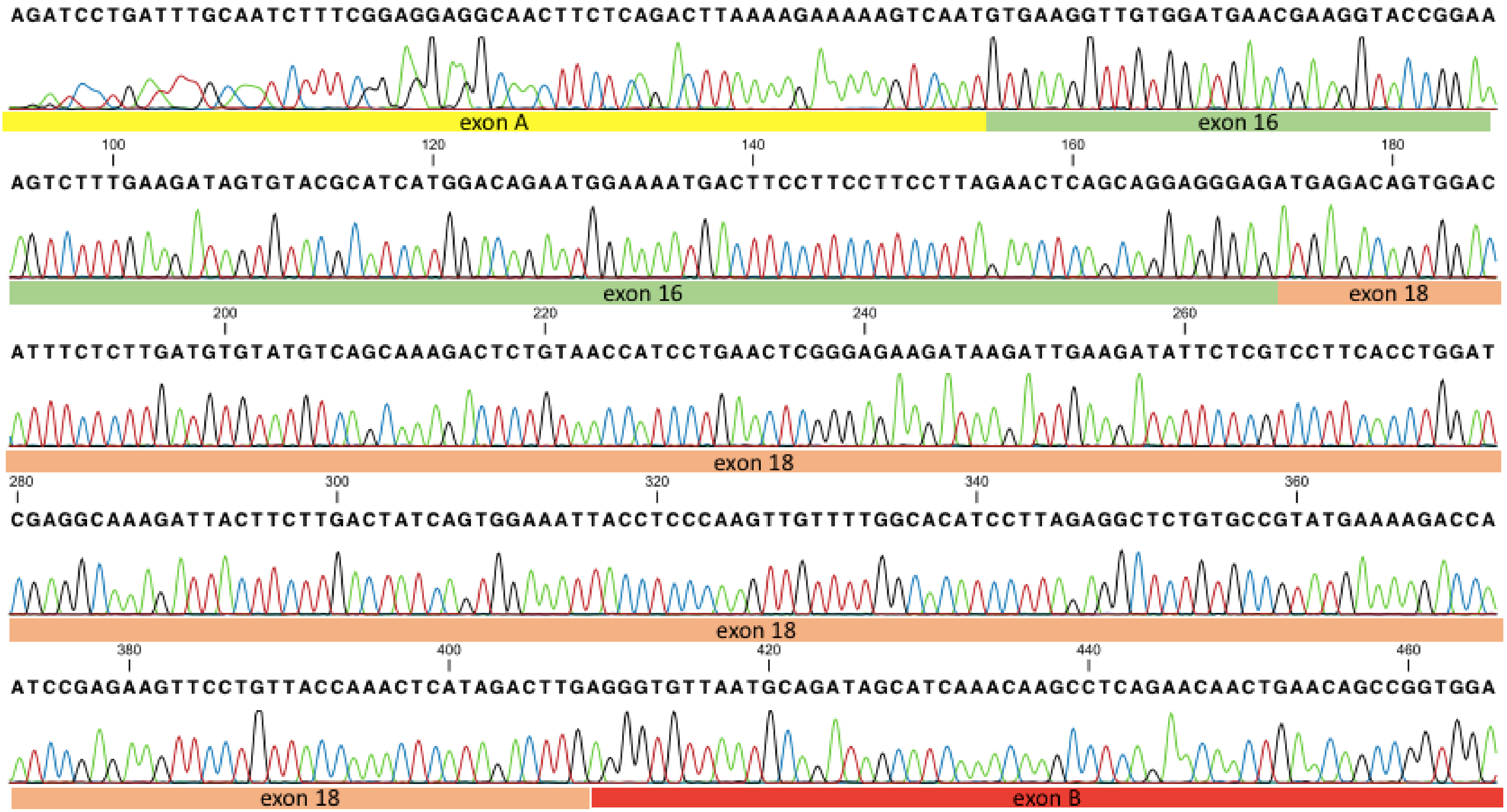

c.1879+5 G > A

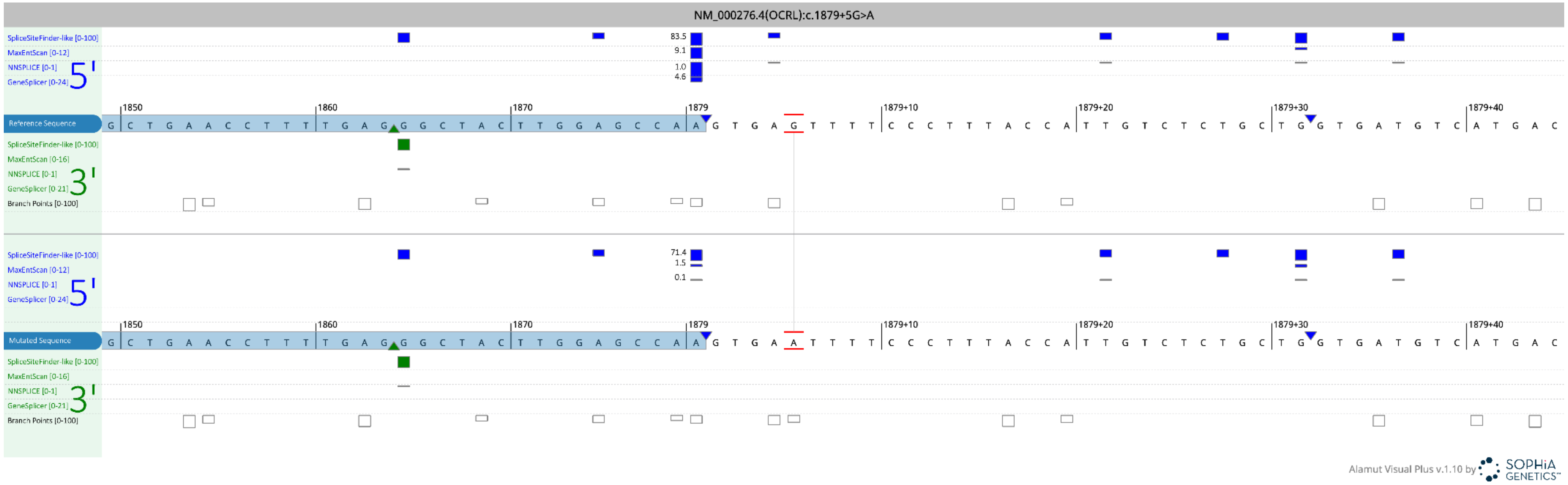

SpliceAI scores:

Variant	Gene ☐ = MANE Select transcript ☐ = non-coding transcript	Δ type	Δ score	position	REF score	ALT score
NM_000276.4(OCRL):c.1879+5 G>A<br>⇒ *X:129576067 G>A*<br>UCSC, gnomAD	OCRL<br>(ENSG00000122126.18 / ENST00000371113.9 / NM_000276.4)<br>protein coding MANE Select transcript (plus strand)<br>OMIM, GTEx, gnomAD, ClinGen, Ensembl, Decipher, GeneCards	Acceptor Loss	0.06	-170 bp	1.00	0.94
		Donor Loss	0.92	-5 bp	0.99	0.07
		Acceptor Gain	0.00	-496 bp	0.01	0.01
		Donor Gain	0.70	-102 bp	0.04	0.75

21. c.2256+1 G > A

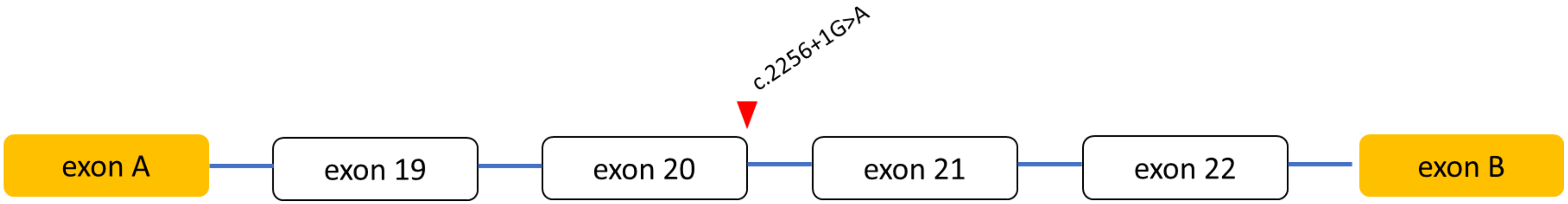

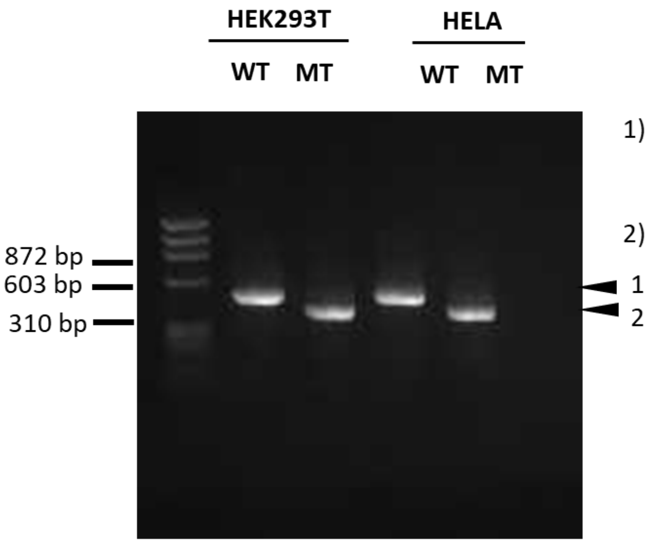

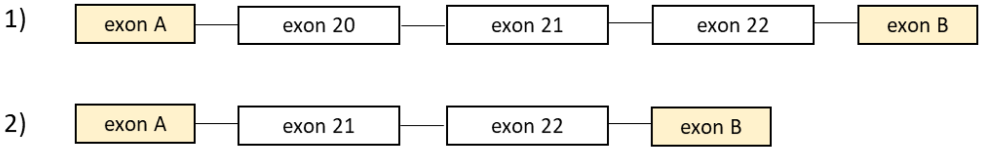

RT-PCR-amplified products of c.2256+1G>A hybrid minigene transcripts

※*OCRL* have been reported to have two splice variants, one containing exon 19 and the other not containing exon 19, which is 24-bp long. Furthermore, the splice variant is tissue-specific, with many organs, including kidney, predominantly expressing a splicing variant that does not contain exon 19. These suggest that exon 19 is likely to be skipped in nature.

c.2256+1 G > A band No. 1

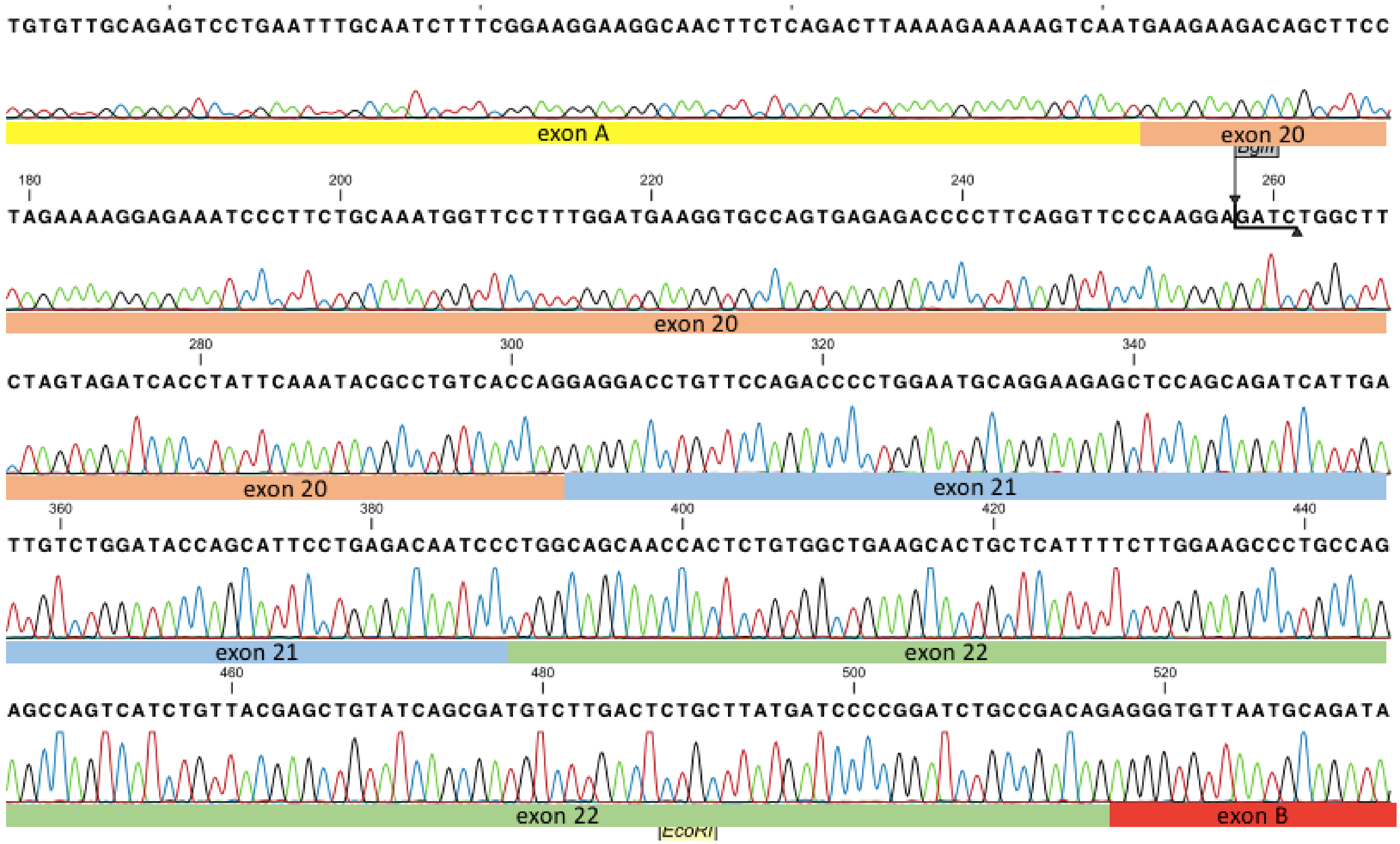

c.2256+1 G > A band No. 2

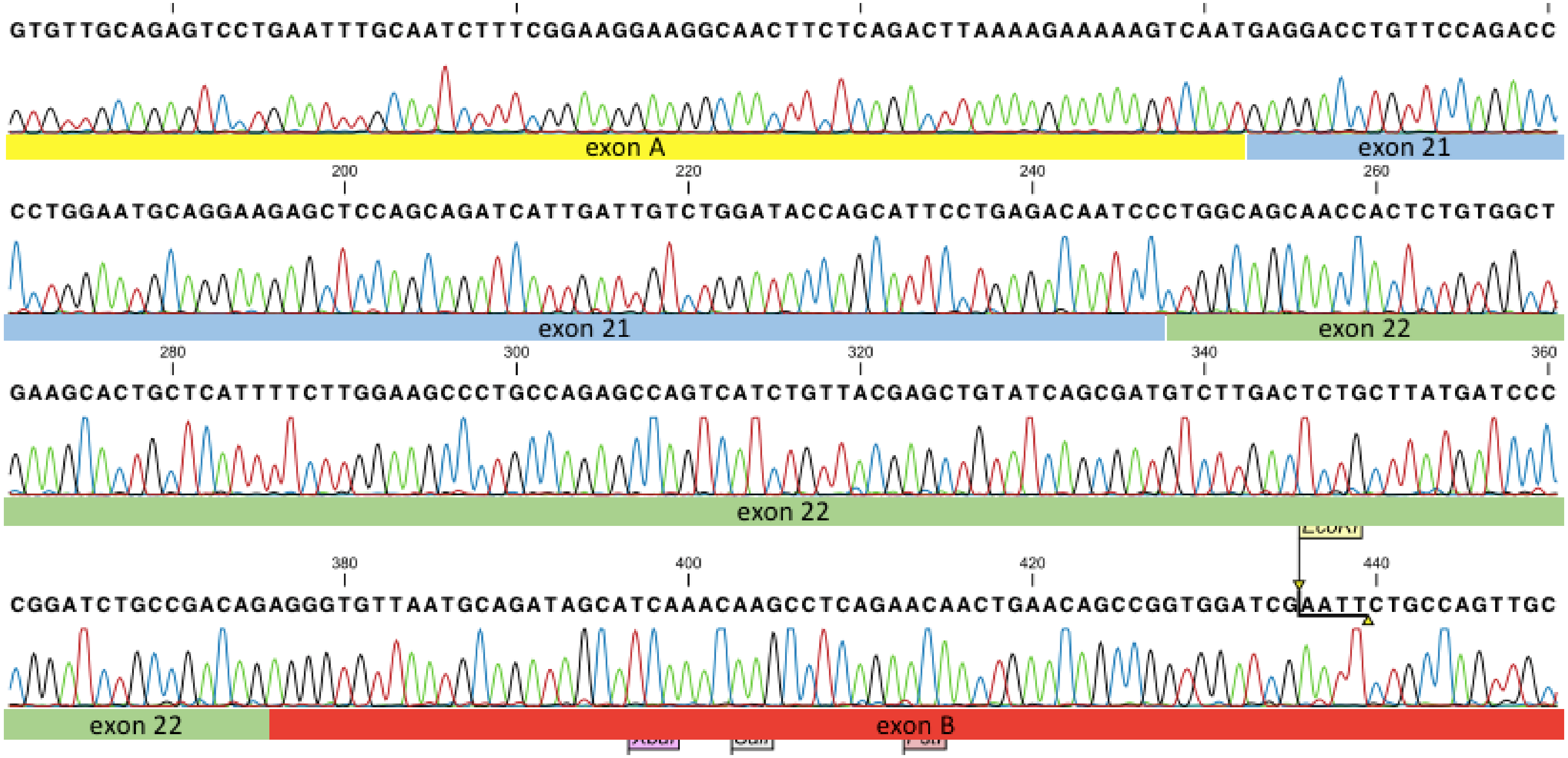

c.2256+1 G >A

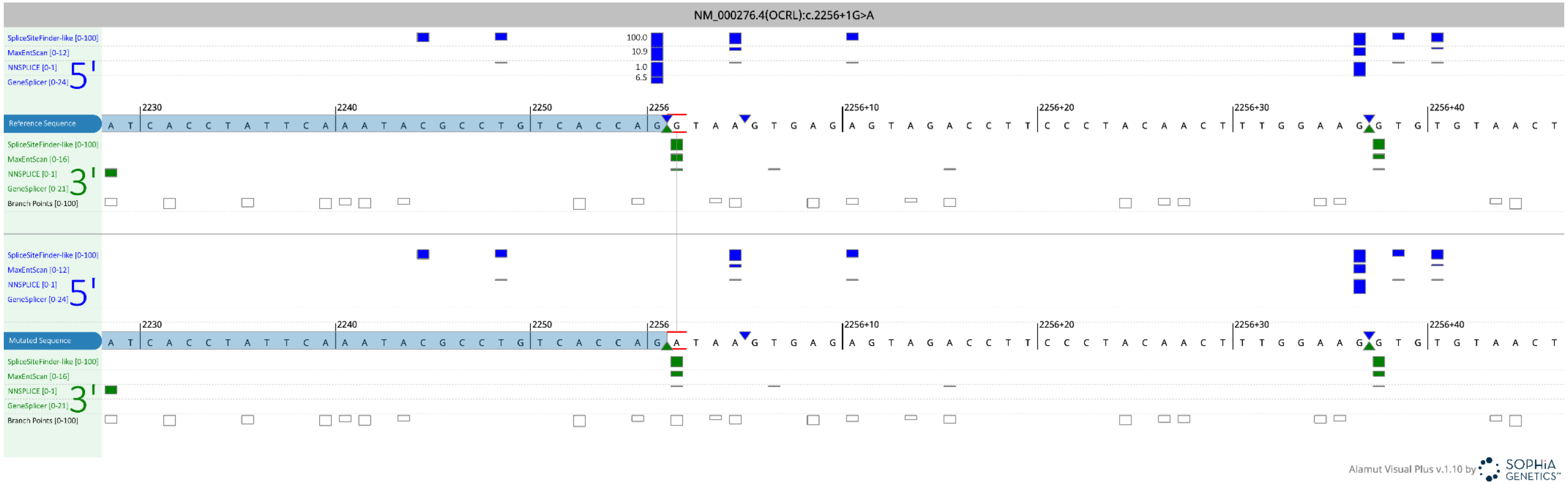

SpliceAI scores:

Variant	Gene (☐ = MANE Select transcript ☐ = non-coding transcript)	Δ type	Δ score	position	REF score	ALT score
NM_000276.4(OCRL):c.2256+1 G>A<br>⇒ *X:129587119 G>A*<br>UCSC, gnomAD	OCRL<br>(ENSG00000122126.18 / ENST00000371113.9 / NM_000276.4)<br>protein coding MANE Select transcript<br>(plus strand)<br>OMIM, GTEx, gnomAD, ClinGen, Ensembl, Decipher, GeneCards	Acceptor Loss	0.58	-117 bp	0.99	0.41
		Donor Loss	1.00	-1 bp	1.00	0.00
		Acceptor Gain	0.01	-470 bp	0.02	0.03
		Donor Gain	0.41	35 bp	0.00	0.41

22. c.2341+1 G > C

23. c.2469+2 T > G

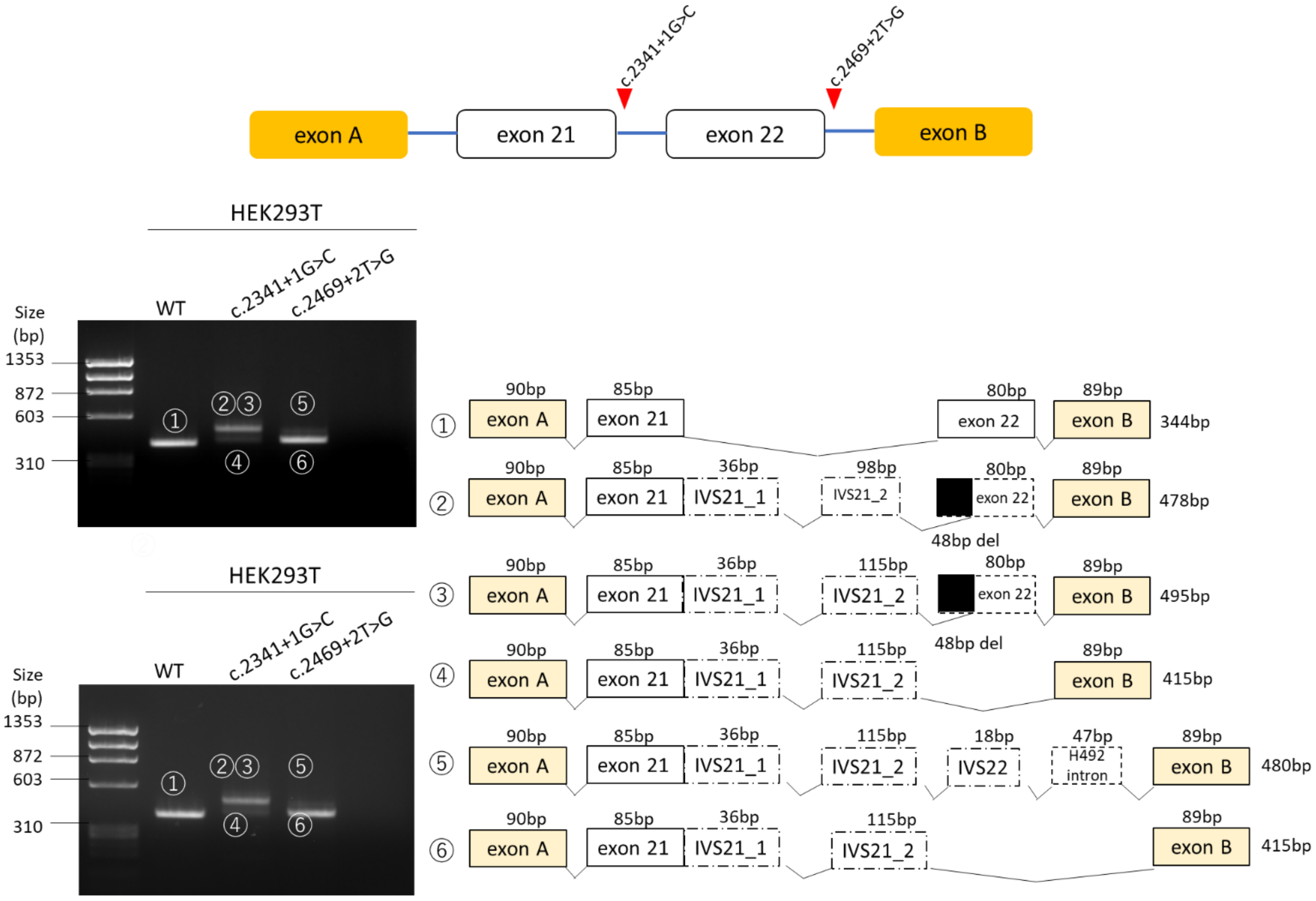

RT-PCR-amplified products of c.2341+1G>C and 2469+2T>G hybrid minigene transcripts

c.2341+1 G > C, c.2469+2 T > G band No. 1

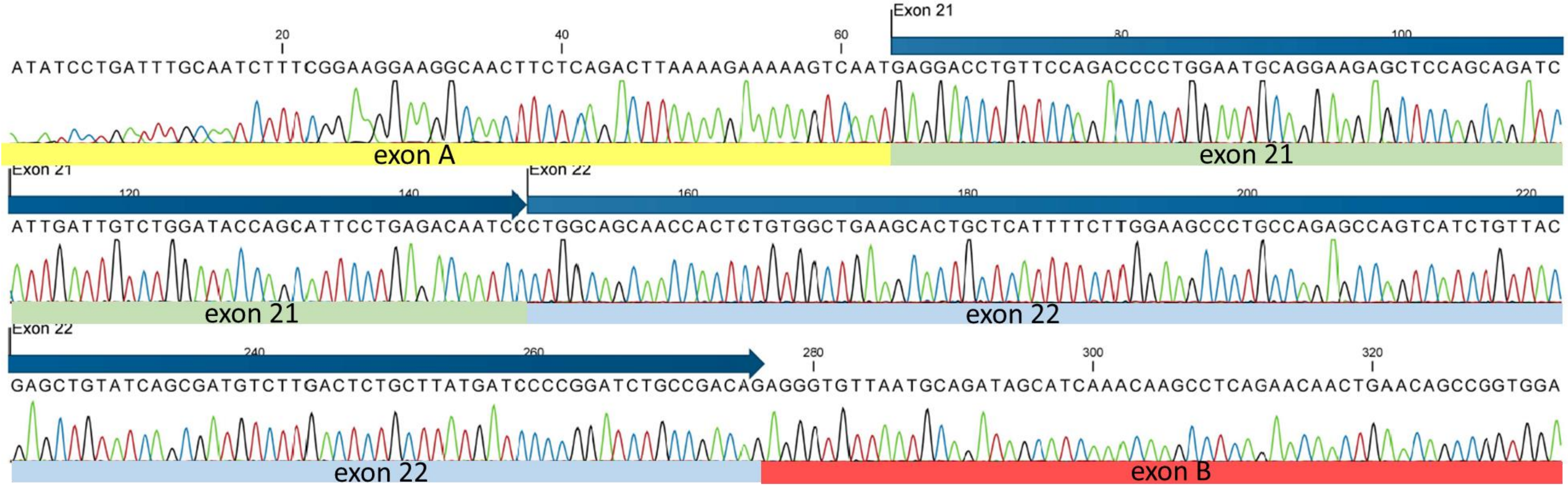

c.2341+1 G > C band No. 2

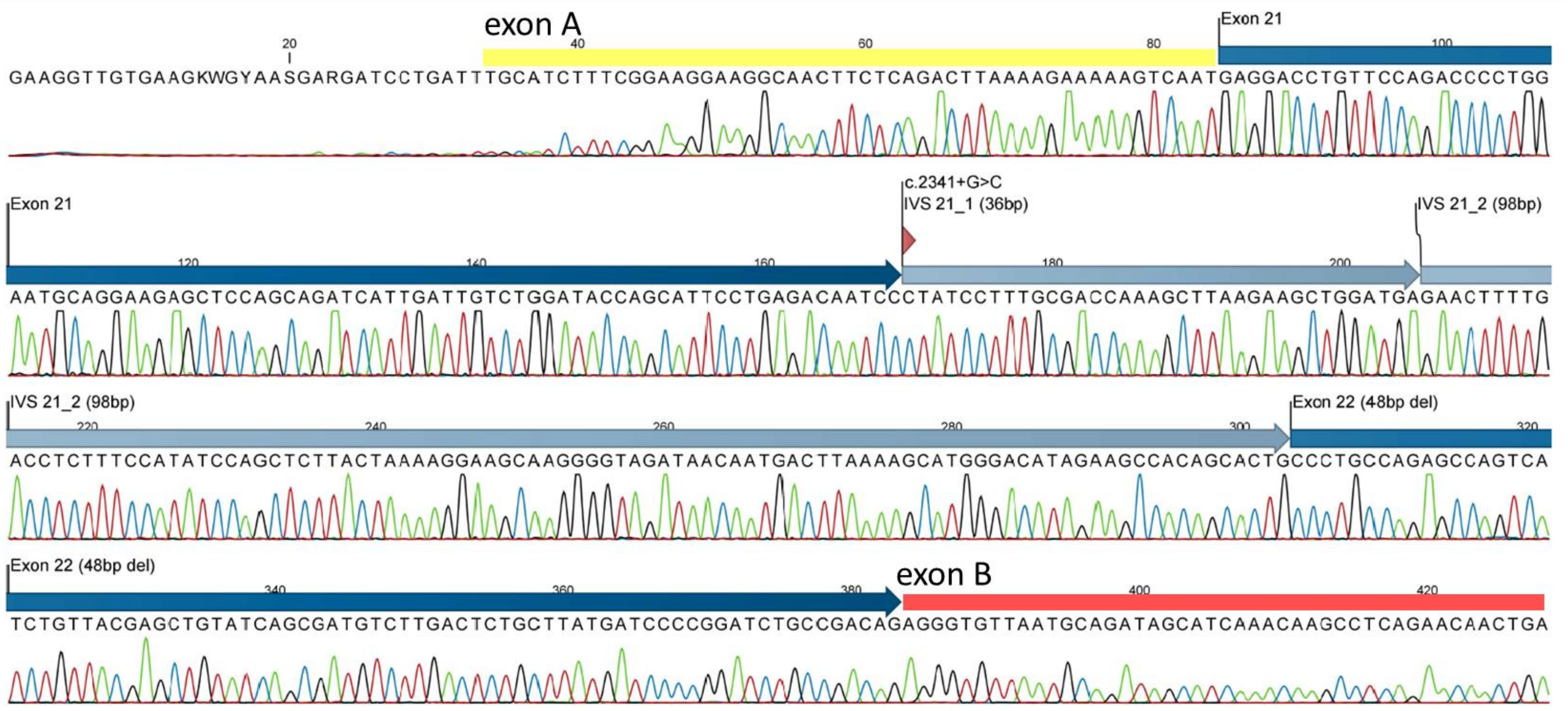

c.2341+1 G > C band No. 3

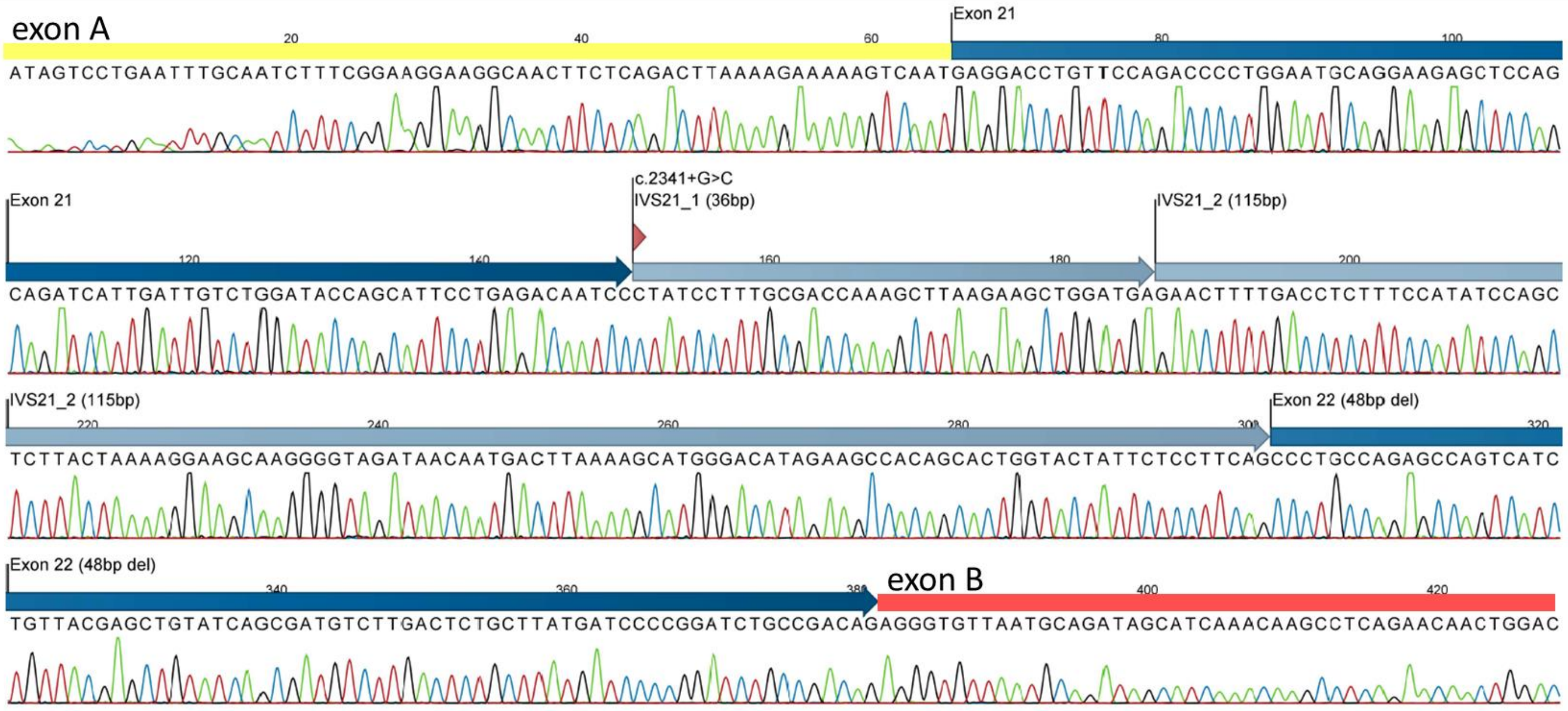

c.2341+1 G > C band No. 4

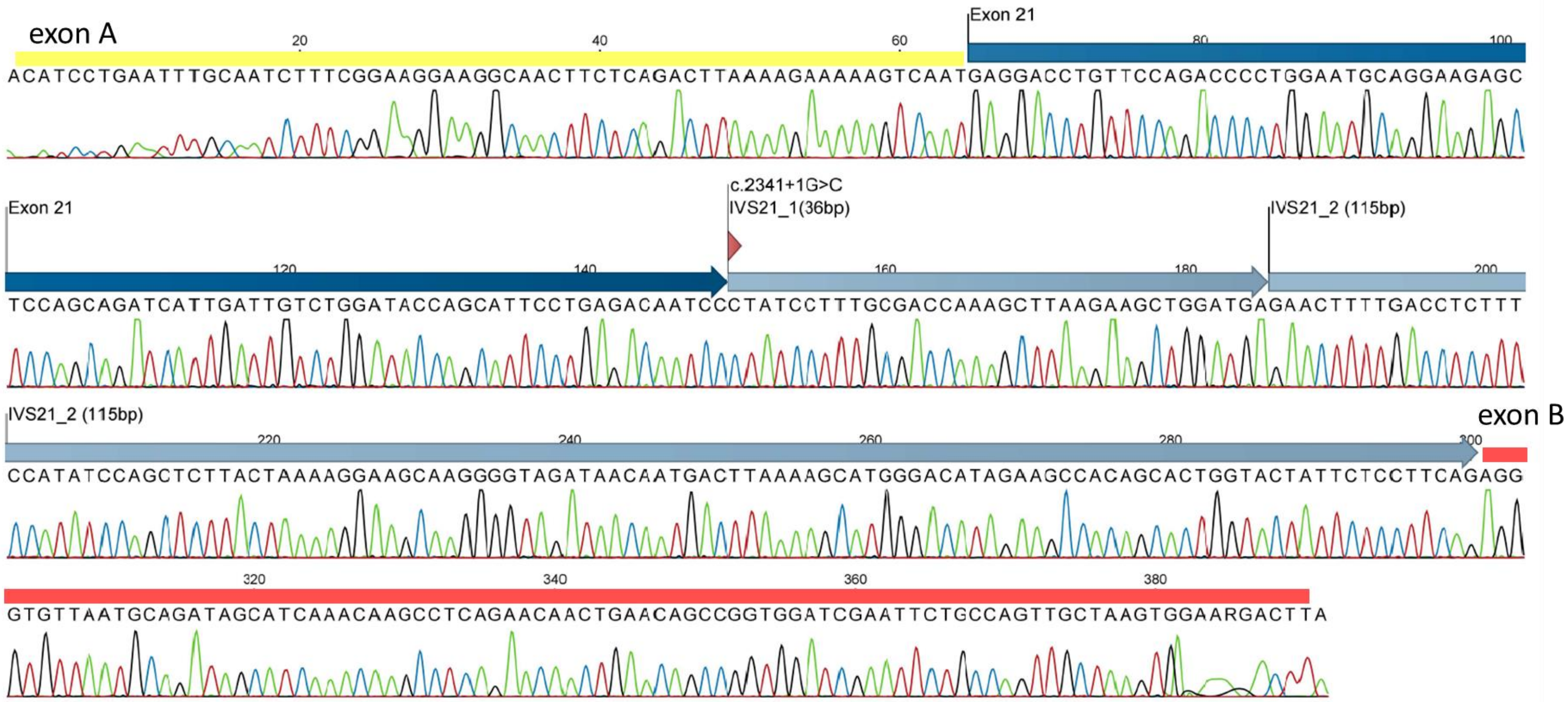

c.2469+2 T > G band No. 5

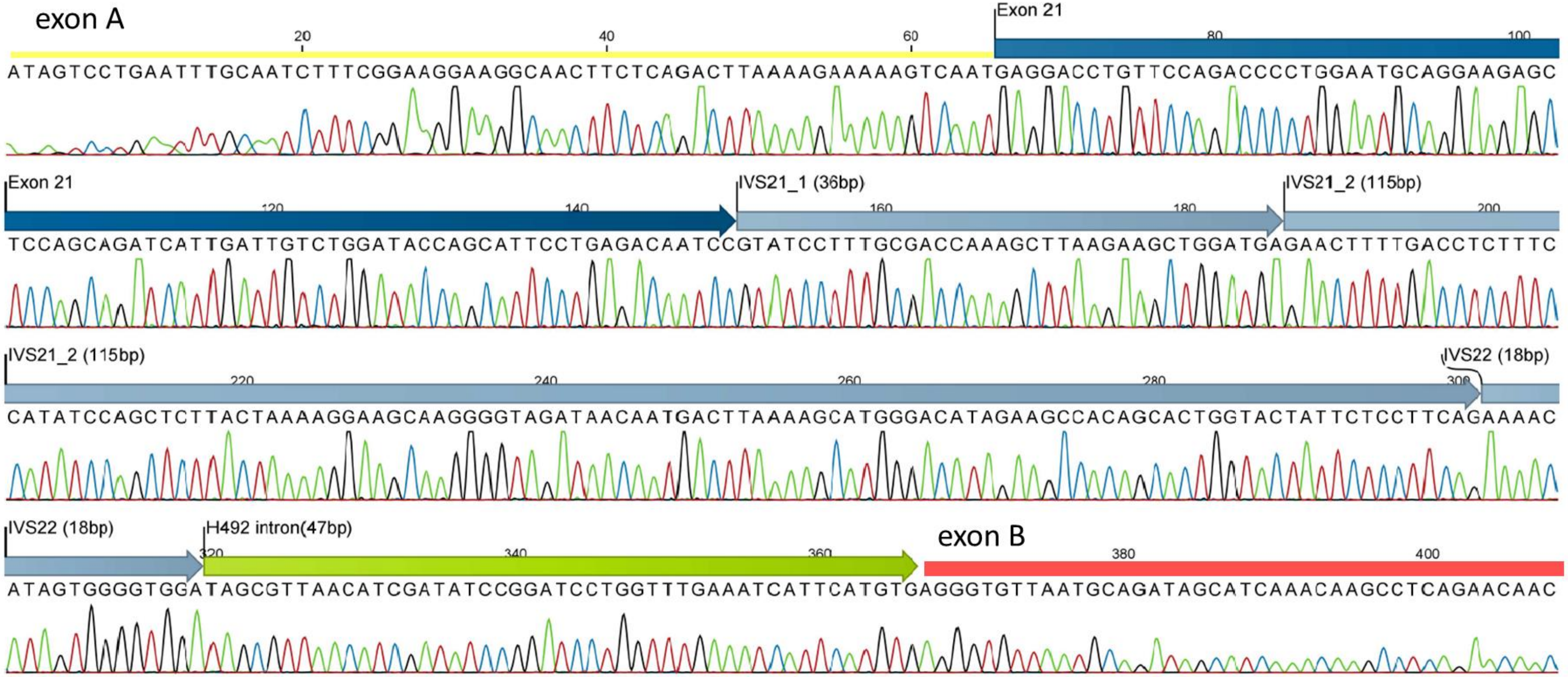

c.2469+2 T > G band No. 6

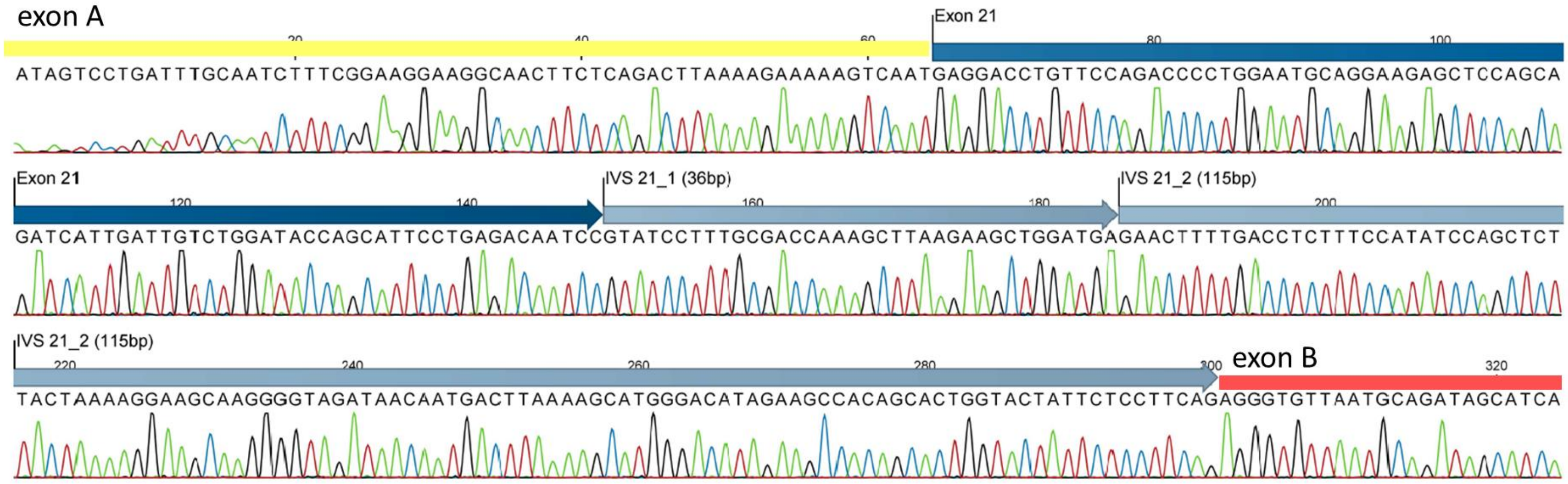

c.2341+1 G > C

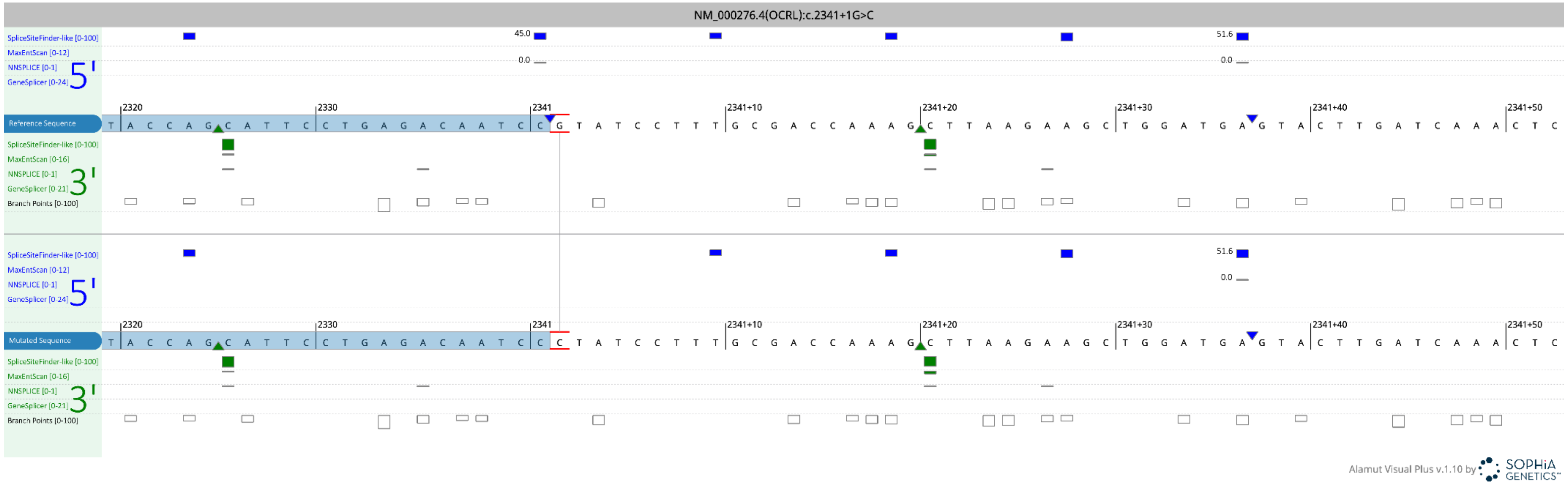

SpliceAI scores: ?

Variant	Gene (☐ = MANE Select transcript ☐ = non-coding transcript)	Δ type	Δ score	position	REF score	ALT score
NM_000276.4(OCRL):c.2341+1 G>C<br>⇒ *X:129588264 G>C*<br>UCSC, gnomAD	OCRL<br>(ENSG00000122126.18 / ENST00000371113.9 / NM_000276.4)<br>protein coding MANE Select transcript (plus strand)<br>OMIM, GTEx, gnomAD, ClinGen, Ensembl, Decipher, GeneCards	Acceptor Loss	0.11	-85 bp	0.96	0.85
		Donor Loss	0.60	-1 bp	0.82	0.22
		Acceptor Gain	0.00		0.00	0.00
		Donor Gain	0.08	35 bp	0.15	0.23

c.2469+2 T > G

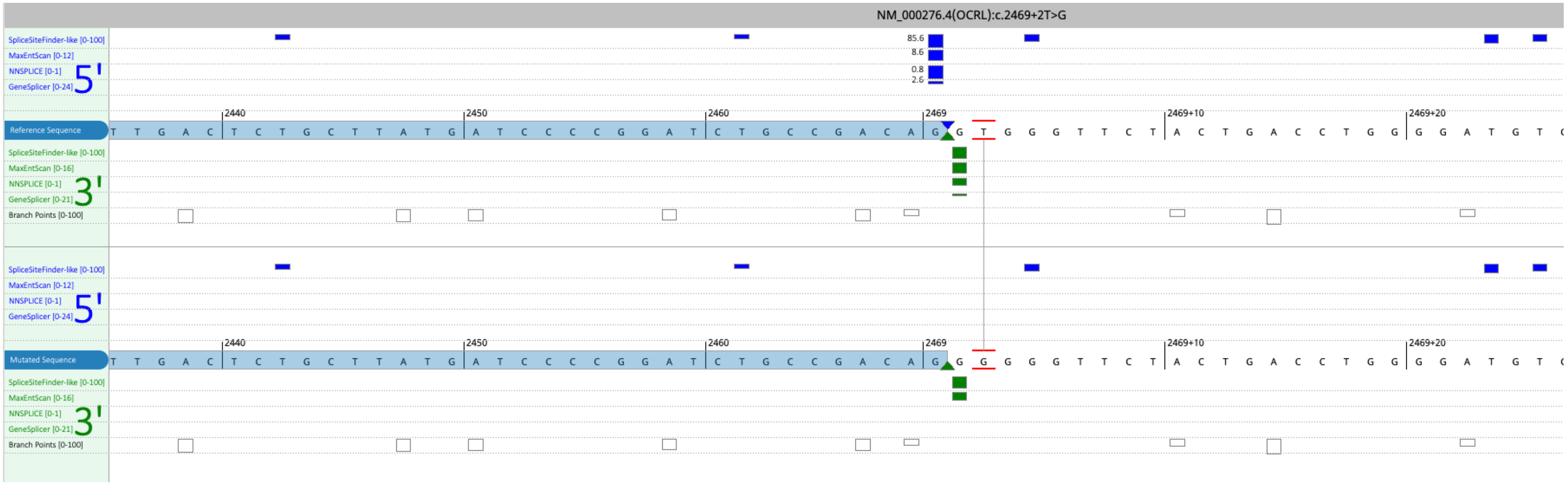

SpliceAI scores:

Variant	Gene (☐ = MANE Select transcript ☐ = non-coding transcript)	Δ type	Δ score	position	REF score	ALT score
NM_000276.4(OCRL):c.2469+2 T>G<br>⇒ *X:129589015 T>G*<br>UCSC, gnomAD	OCRL<br>(ENSG00000122126.18 / ENST00000371113.9 / NM_000276.4)<br>protein coding MANE Select transcript<br>(plus strand)<br>OMIM, GTEx, gnomAD, ClinGen, Ensembl, Decipher, GeneCards	Acceptor Loss	0.60	-81 bp	0.61	0.01
		Donor Loss	0.96	-2 bp	0.96	0.00
		Acceptor Gain	0.00		0.00	0.00
		Donor Gain	0.07	91 bp	0.02	0.08

24. c.2581 G > A

25. c.2581 G > C

26. c.2581+1 G > A

27. c.2581+1 G > C

28. c.2581+4 A > G

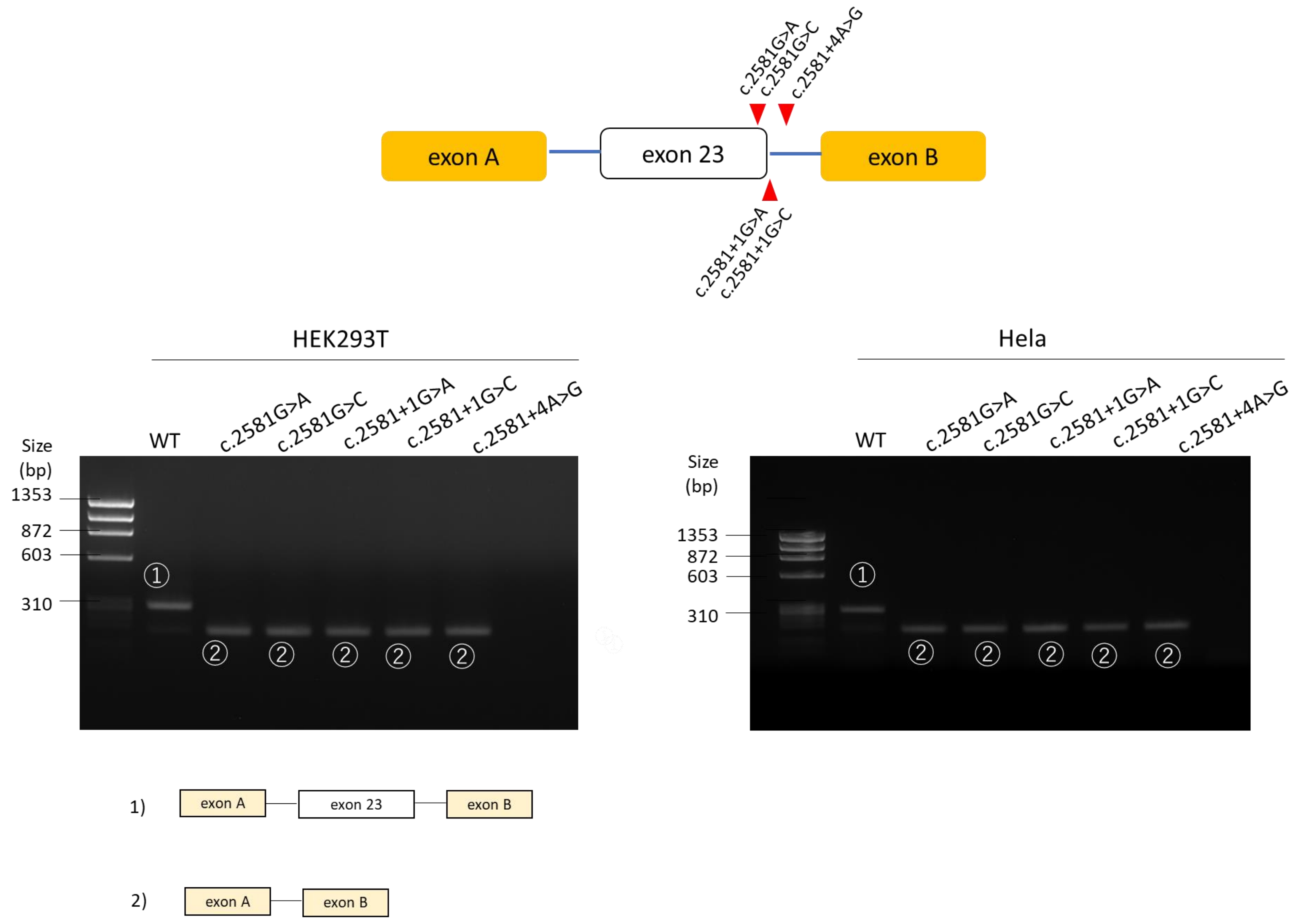

RT-PCR-amplified products of c.2581G>A, c.2581G>C, c.2581+1G>A, c.2581+1G>C and c.2581+4A>G hybrid minigene transcripts

c.2581 G > A, c.2581 G > C, c.2581+1 G > A, c.2581+1 G > C, c.2581+4 A > G band No. 1

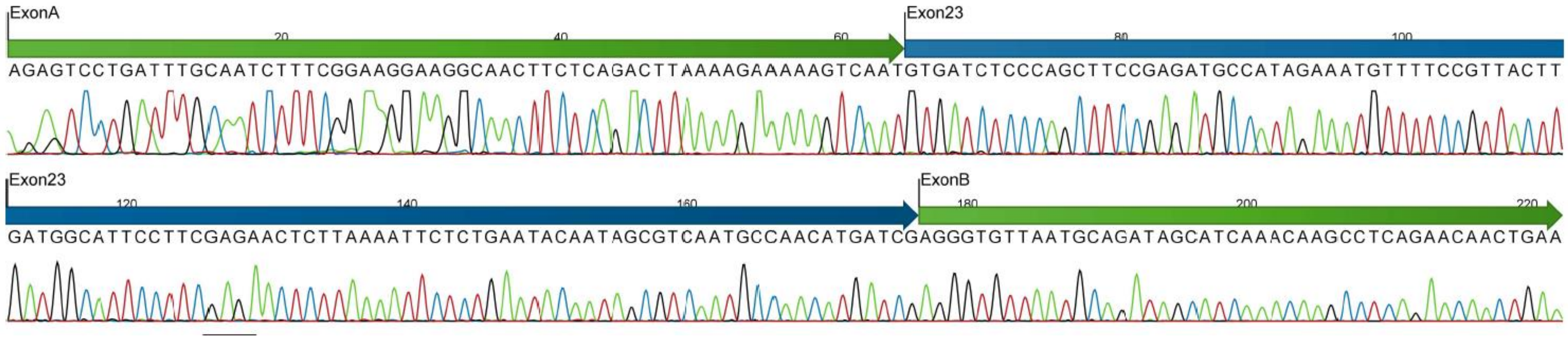

c.2581 G > A band No. 2

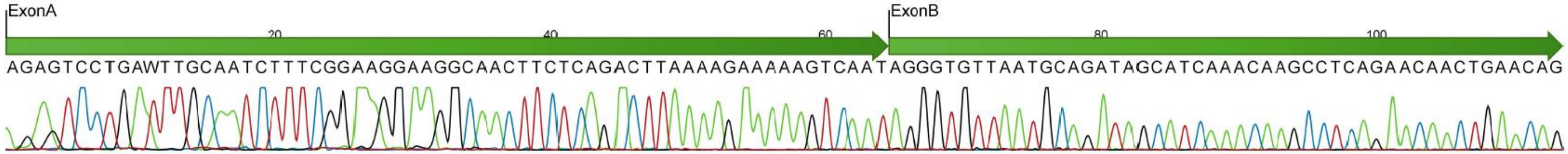

c.2581 G > C band No. 2

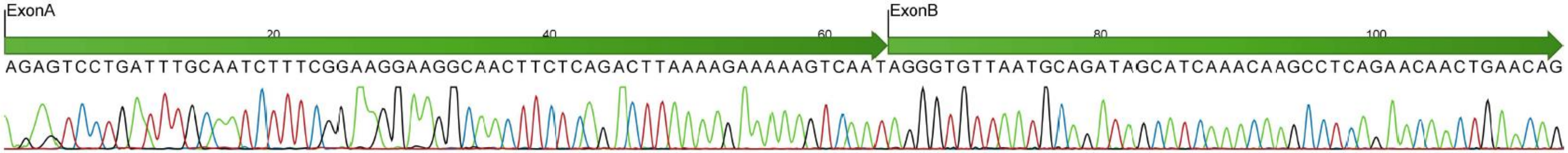

c.2581+1 G > A band No. 2

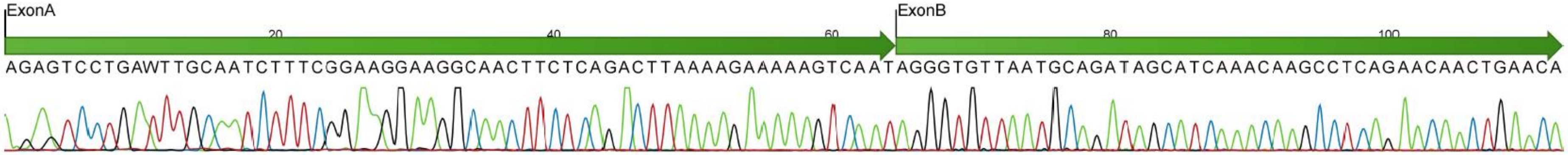

c.2581+1 G > C band No. 2

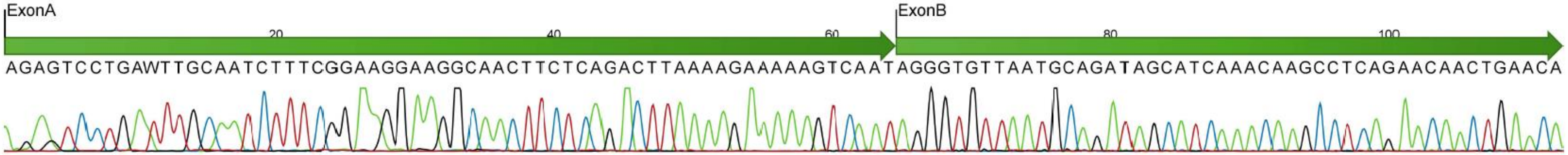

c.2581+4 A > G band No. 2

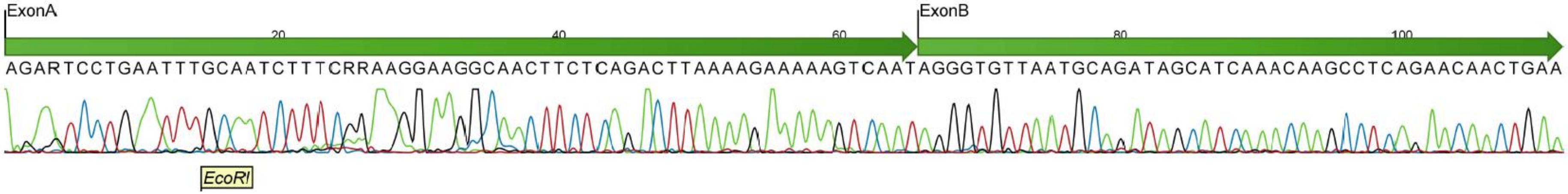

c.2581 G > A

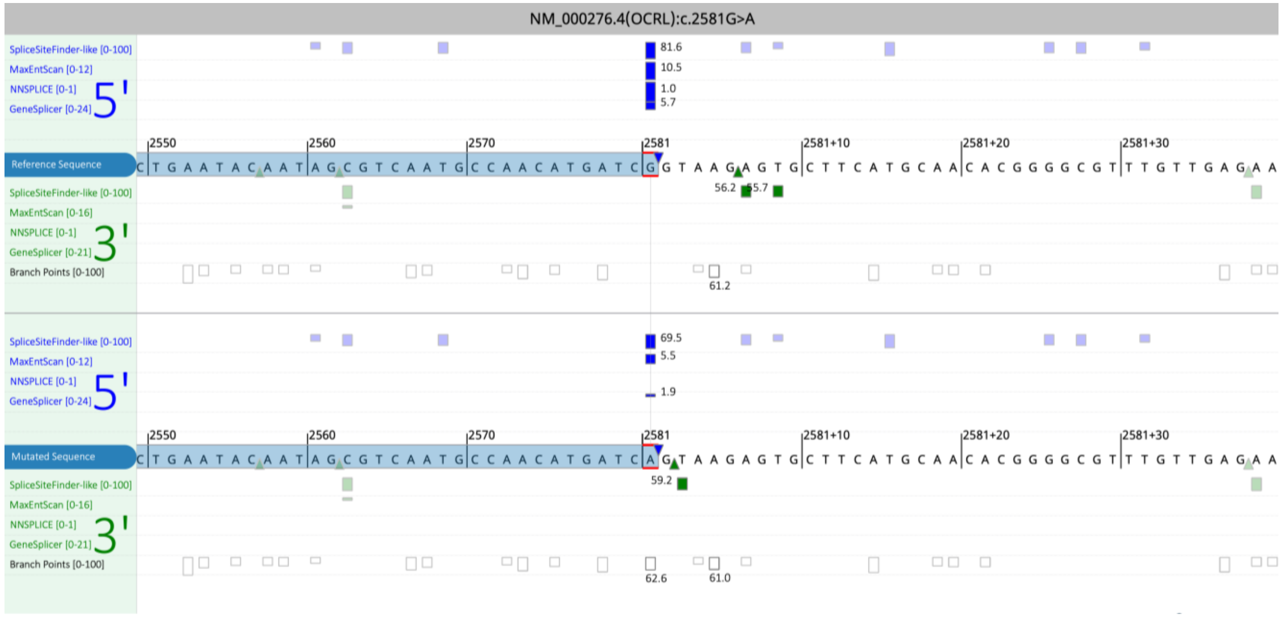

SpliceAI scores:

Variant	Gene (= MANE Select transcript, = non-coding transcript)	Δ type	Δ score	position	REF score	ALT score
NM_000276.4(OCRL):c.2581 G>A<br>⇒ X:129589956 G>A<br>UCSC, gnomAD	OCRL<br>(ENSG00000122126.18 / ENST00000371113.9 / NM_000276.4)<br>protein coding MANE Select transcript<br>(plus strand)<br>OMIM, GTEx, gnomAD, ClinGen, Ensembl, Decipher, GeneCards	Acceptor Loss	0.40	-111 bp	0.99	0.59
		Donor Loss	0.53	0 bp	0.95	0.42
		Acceptor Gain	0.00		0.00	0.00
		Donor Gain	0.00		0.00	0.00

25. c.2581 G > C

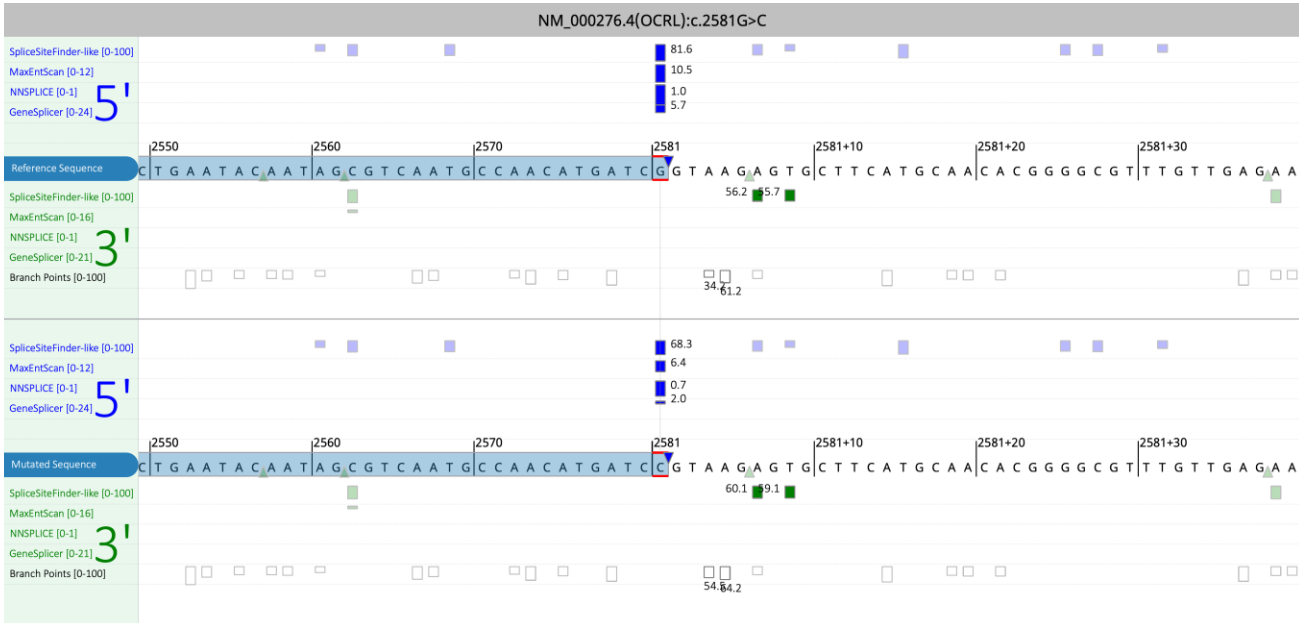

SpliceAI scores:

Variant	Gene ▢ = MANE Select transcript ▢ = non-coding transcript	Δ type	Δ score	position	REF score	ALT score
NM_000276.4(OCRL):c.2581 G>C<br>⇒ X:129589956 G>C<br>UCSC, gnomAD	OCRL<br>(ENSG00000122126.18 / ENST00000371113.9 / NM_000276.4)<br>protein coding MANE Select transcript<br>(plus strand)<br>OMIM, GTEx, gnomAD, ClinGen, Ensembl, Decipher, GeneCards	Acceptor Loss	0.34	-111 bp	0.99	0.65
		Donor Loss	0.57	0 bp	0.95	0.37
		Acceptor Gain	0.00		0.00	0.00
		Donor Gain	0.01	54 bp	0.00	0.01

26. c.2581+1 G > A

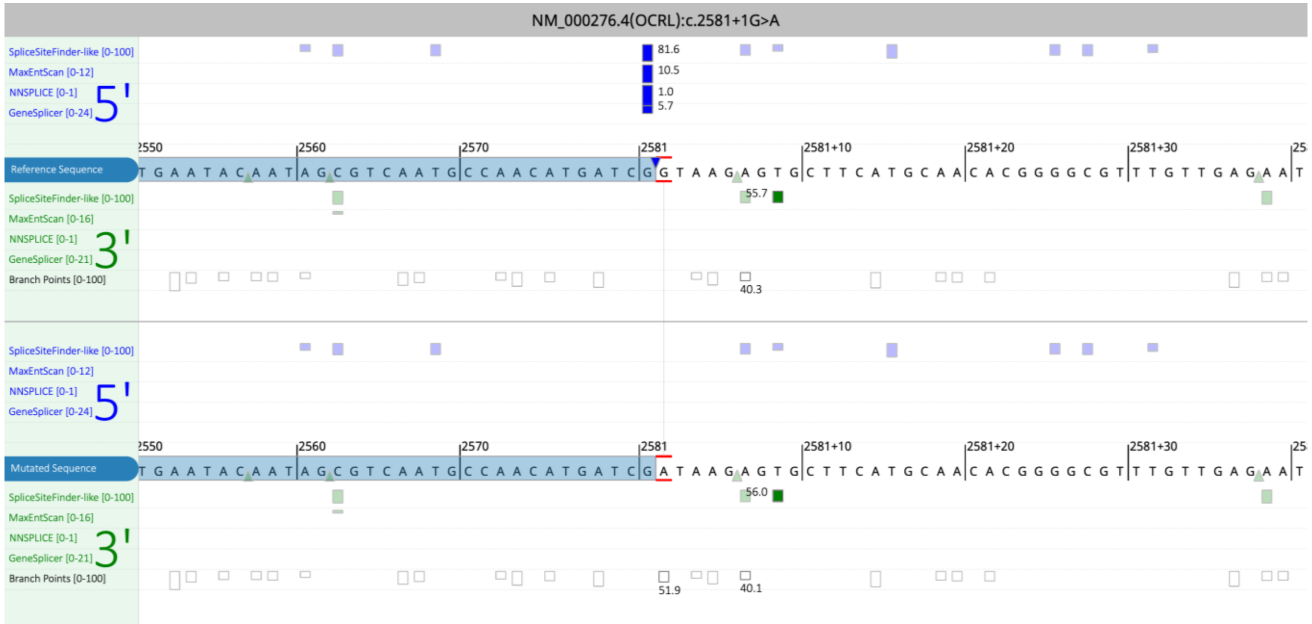

SpliceAI scores: ?

Variant	Gene ☐ = MANE Select transcript ☐ = non-coding transcript	Δ type	Δ score	position	REF score	ALT score
NM_000276.4:c.2581+1 G>A ⇒ X:129589957 G>A UCSC, gnomAD	OCRL (ENSG00000122126.18 / ENST00000371113.9 / NM_000276.4) protein coding MANE Select transcript (plus strand) OMIM, GTEx, gnomAD, ClinGen, Ensembl, Decipher, GeneCards	Acceptor Loss	0.58	-112 bp	0.99	0.42
		Donor Loss	0.95	-1 bp	0.95	0.00
		Acceptor Gain	0.00		0.00	0.00
		Donor Gain	0.02	53 bp	0.00	0.02

27. c.2581+1 G > C

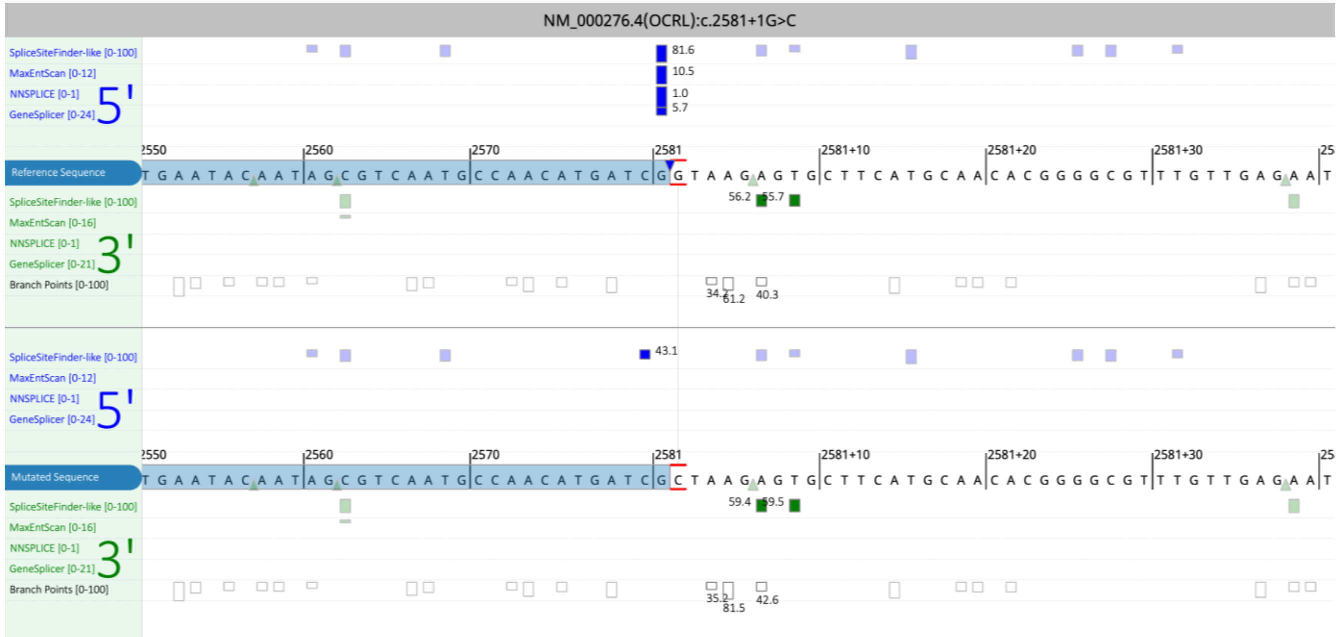

SpliceAI scores:

Variant	Gene (= MANE Select transcript; = non-coding transcript)	Δ type	Δ score	position	REF score	ALT score
NM_000276.4:c.2581+1 G>C<br>⇒ *X:129589957 G>C*<br>UCSC, gnomAD	OCRL<br>(ENSG00000122126.18 / ENST00000371113.9 / NM_000276.4)<br>protein coding MANE Select transcript<br>(plus strand)<br>OMIM, GTEx, gnomAD, ClinGen, Ensembl, Decipher, GeneCards	Acceptor Loss	0.65	-112 bp	0.99	0.34
		Donor Loss	0.95	-1 bp	0.95	0.00
		Acceptor Gain	0.00		0.00	0.00
		Donor Gain	0.02	53 bp	0.00	0.02

28. c.2581+4 A > G

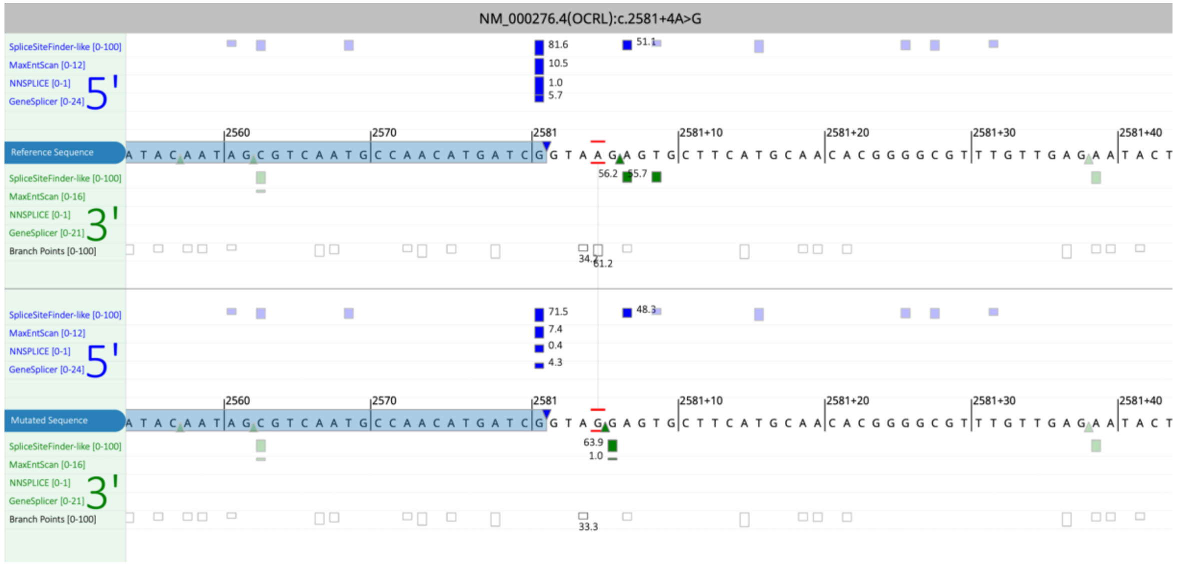

SpliceAI scores:

Variant	Gene (= MANE Select transcript; = non-coding transcript)	Δ type	Δ score	position	REF score	ALT score
NM_000276.4:c.2581+4A>G<br>⇒ *X:129589960 A>G*<br>UCSC, gnomAD	OCRL<br>(ENSG00000122126.18 / ENST00000371113.9 / NM_000276.4)<br>protein coding MANE Select transcript<br>(plus strand)<br>OMIM, GTEx, gnomAD, ClinGen, Ensembl, Decipher, GeneCards	Acceptor Loss	0.20	-115 bp	0.99	0.79
		Donor Loss	0.47	-4 bp	0.95	0.48
		Acceptor Gain	0.00		0.00	0.00
		Donor Gain	0.00		0.00	0.00

Supplementary Figure S1. mRNA analysis using the minigene system. The transcriptional result, base sequences, and *in silico* analysis of the *OCRL* splicing variant.

The aberrant splicing was observed in all variants, excluding c.40-14. There were various splicing patterns, including exon skipping, inclusion of cryptic exons, loss of an exon fragment, and inclusion of an intron fragment.

The upper panels show the transcriptional analysis results of the minigene system for the *OCRL* splicing variants. The upper part of the figure shows each inserted fragment with individual exons and flanking introns. The lower left part of the figure shows agarose gel electrophoresis of the RT-PCR product of the minigene system in HEK293T and HeLa cells. The lower right part of the figure is a schematic representation of the splicing outcome.

The middle panels show the sequences of RT-PCR products generated from the minigene system.

The lower panels show the *in silico* analysis result of *OCRL* splicing variant assessed using Alamut and SpliceAI-lookup. All splice site variants were predicted to produce aberrant splicing through the disruption of donor, acceptor, and branch point sites. The threshold is defined at 65 for SpliceSiteFinder-like and 3 for MaxEntScan. This indicates that every signal with a score above the threshold is considered a splice site (donor or acceptor). For NNSplice, the output score is 0–1, and a higher score implies a greater potential for splice site. When a mutation occurs, it is considered to break the splice site if the wild type (WT) score is above the threshold and the score variation (between WT and mutant) is < -10% for SpliceSiteFinder-like (< -30 % for MaxEntScan). In contrast, if the WT score is below the threshold and the score variation is > +10% for SpliceSiteFinder-like (> +30 % for MaxEntScan), it is considered to create a novel splice site. The score variation of certain variants cannot be determined because of low threshold value (Human Splicing Finder, Help & Tutorials; Jian, Boerwinkle, Liu. *Genet in Med*. 2014).

Additionally, we assessed the splicing variants using SpliceAI-lookup with a threshold distance of 500 bp. The authors of SpliceAI recommend a value of 0.2 as a threshold of four Δ scores to distinguish between variants that can alter splicing and those that do not.
